# The Proceedings of the 19th International Cancer Imaging Society Meeting and Annual Teaching Course: Verona, Italy. 7 - 9 October 2019

**DOI:** 10.1186/s40644-019-0244-2

**Published:** 2019-09-12

## Monday 7^th^ October - Morning Session: 9:00 – 10.30 Clinical Needs In Pancreatic Imaging

### A1 Ductal adenocarcinoma: detection and staging

#### Mirko D’Onofrio

##### School of Medicine and Surgery, University of Verona, Italy

Computed Tomography (CT) diagnoses ductal adenocarcinoma with sensitivity and specificity from 70% to 100% and is therefore always indicated (1). However, 27% of pancreatic adenocarcinomas with smaller than 2 cm are isodense at TC (2). The secondary signs are present in a statistically different percentage in relation to the dimensions, if tumour size is lower or greater than 2 cm Magnetic Resonance Imaging (MR) is superior compared to CT in the small pancreatic tumour detection (3). Choi TW et al report how the sensitivity of MRI in direct identification of small adenocarcinoma is statistically superior compared to CT (4). Magnetic resonance imaging is also superior to CT for the identification of hepatic metastases (5). In patients with potentially resectable pancreatic adenocarcinoma, Magnetic Resonance with diffusion weighted sequences (DWI) significantly improves the diagnostic performance in the characterisation of focal liver lesions, especially if small (≤ 1 cm), identified as CT, or indeterminate, improving hepatic staging (5). Regarding local staging, CT has a positive predictive value of nonresectability ranging from 89% to 100% while the positive predictive value of resectability is lower(6).

Metanalysis on EUS demonstrates a better definition of vascular invasion (7, 8). EUS has an important diagnostic accuracy regarding the description of a possible vascular invasion, and therefore for the definition of resectability (9).

Evaluation of pancreatic ductal adenocarcinoma (PDAC) after chemoradiotherapy downstaging is challenging due to computed tomography (CT) overestimation of tumour extension and residual vascular involvement. Imaging methods tend to underestimate PDAC resectability after neoadjuvant therapy. Surgery should be considered for downstaged borderline resectable PDACs, independently from perivascular cuff presence, especially for tumours smaller than 25 mm (10).

References

1. Schima W, Ba-Ssalamah A, Kolblinger C et al (2007) Pancreatic adenocarcinoma. Eur Radiol 17(3):638-49

2. Yoon SH, Lee JM, Cho JY, Lee KB, Kim JE, Moon SK, Kim SJ, Baek JH, Kim SH, Kim SH, Lee JY, Han JK, Choi BI. Small (≤ 20 mm) pancreatic adenocarcinomas: analysis of enhancement patterns and secondary signs with multiphasic multidetector CT. Radiology. 2011 May;259(2):442-52.

3. Park HS, Lee JM, Choi HK, Hong SH, Han JK, Choi BI. Preoperative evaluation of pancreatic cancer: comparison of gadolinium-enhanced dynamic MRI with MR cholangiopancreatography versus MDCT. J Magn Reson Imaging. 2009 Sep;30(3):586-95

4. Choi TW, Lee JM, Kim JH, Yu MH, Han JK, Choi BI. Comparison of Multidetector CT and Gadobutrol-Enhanced MR Imaging for Evaluation of Small, Solid Pancreatic Lesions. Korean J Radiol. 2016 Jul-Aug;17(4):509-21.

5. Jeon SK, Lee JM, Joo I, Lee DH, Ahn SJ, Woo H, Lee MS, Jang JY, Han JK. Magnetic resonance with diffusion-weighted imaging improves assessment of focal liver lesions in patients with potentially resectable pancreatic cancer on CT. Eur Radiol. 2018 Jan 19.

6. Al-Hawary MM, Francis IR, Chari ST, Fishman EK, Hough DM, Lu DS, Macari M, Megibow AJ, Miller FH, Mortele KJ, Merchant NB, Minter RM, Tamm EP, Sahani DV, Simeone DM. Pancreatic ductal adenocarcinoma radiology reporting template: consensus statement of the society of abdominal radiology and the American pancreatic association. Gastroenterology. 2014 Jan;146(1):291-304.

7. Treadwell JR1, Zafar HM, Mitchell MD, et al. Imaging Tests for the Diagnosis and Staging of Pancreatic Adenocarcinoma: A Meta-Analysis. Pancreas. 2016 Jul;45(6):789-95.

8. Yang R, Lu M, Qian X, et al. Diagnostic accuracy of EUS and CT of vascular invasion in pancreatic cancer: a systematic review. J Cancer Res ClinOncol. 2014 Dec;140(12):2077-86.

9. Li JH, He R, Li YM et al. Endoscopic ultrasonography for tumour node staging and vascular invasion in pancreatic cancer: a meta-analysis.Dig Surg. 2014;31(4-5):297-305.

10. Beleù A, Calabrese A, Rizzo G, Capelli P, Bellini N, Caloggero S, Calbi R, Tinazzi Martini P, De Robertis R, Carbognin G, Marchegiani G, Scarpa A, Salvia R, Bassi C, D'Onofrio M. Preoperative Imaging Evaluation after Downstaging of Pancreatic Ductal Adenocarcinoma: A Multi-Center Study. Cancers (Basel). 2019 Feb 25;11(2). pii: E267. doi: 10.3390/cancers11020267. PubMed PMID: 30823544; PubMed Central PMCID: PMC6406608.

### A2 Pancreatic ductal adenocarcinoma versus mass forming pancreatitis

#### W. Schima

##### Göttlicher Heiland Krankenhaus, Barmherzige Schwestern Krankenhaus, Sankt Josef Krankenhaus, Vinzenzgruppe, Vienna, Austria

Differentiation between pancreatic ductal adenocarcinoma (PDAC) and mass-forming pancreatitis is of utmost importance, as the two entities require completely different treatment strategies and have a different prognosis. However, there is overlap in imaging features, which makes preoperative diagnosis challenging.

Mass-forming pancreatitis can be seen in different clinical scenarios: it may occur in up to 20% of patients with chronic pancreatitis (CP), predominantly in the pancreatic head. To make things even more complicated, the risk of developing ductal adenocarcinoma is markedly increased in patients with CP. PDAC and mass-forming CP present as a hypovascular mass, often with abutment of peripancreatic vessels. The duct-penetrating sign at MRCP is a helpful finding: the vast majority of mass-forming CP shows non-obstruction of the main panc. duct, whereas more than 90% of PDAC (which is more densely fibrotic) demonstrate complete obstruction of the duct (1). In a recent study, perfusion CT was used to differentiate between PDAC and mass-forming CP, which showed that blood volume (BV), blood flow (BF) were lower and mean transit time (MTT) of contrast was longer in PDAC (2).

Paraduodenal pancreatitis (formerly called groove pancreatitis) is a common mimicker of PDAC of the head. In contrast to the pure form of paraduodenal pancreatitis involving the groove between pancreatic head and duodenum only, does the segmental form affect also the pancreatic head, which may be misdiagnosed as neoplasm. In case of cystic components of the mass, suspicion of paraduodenal pancreatitis should be raised. In the solid-tumoural segmental form, a correct imaging diagnosis is unlikely, although parenchymal atrophy is much less often seen than in PDAC of the head (3).

Focal autoimmune pancreatitis (AIP) is a classic pitfall leading to unnecessary pancreatic surgery. Imaging features helpful for making the diagnosis is delayed enhancement on multi-phasic CT (in 100%), longer strictures of the pancreatic duct than those seen in PDAC (mean, 56 mm vs. 16 mm), and considerably less upstream dilation of the duct (max. duct diameter 5 mm in 89%) (4). Muhi et al. (5) confirmed these findings and added the value of ADC maps to differentiate between focal AIP and PDAC. At 1.5T an ADC < 0.88x10^-3^ mm^2^/s is very specific for making the diagnosis of focal AIP rather than PDAC.

In conclusion, differentiation of between PDAC and mass-forming pancreatitis, either in CP, paraduodenal or autoimmune pancreatitis, remains challenging. Multimodality imaging may help make the correct diagnosis in order to avoid unnecessary surgery.

References

1. Ichikawa T, Sou H, Araki T, et al. Duct-penetrating sign at MRCP: usefulness for differentiating inflammatory pancreatic mass from pancreatic carcinomas. Radiology. 2001;221:107-16.

2. Aslan S, Nural MS, Camlidag I, Danaci M. Efficacy of perfusion CT in differentiating of pancreatic ductal adenocarcinoma from mass-forming chronic pancreatitis and characterisation of isoattenuating pancreatic lesions. Abdom Radiol 2019;44:593-603.

3. Muraki T, Kim GE, Reid MD, et al. Paraduodenal Pancreatitis: Imaging and pathologic correlation of 47 cases elucidates distinct subtypes and the factors involved in its etiopathogenesis. Am J Surg Pathol 2017;41:1347-1363.

4. Naitoh I, Nakazawa T, Hayashi K, et al. Clinical differences between mass-forming autoimmune pancreatitis and pancreatic cancer. Scand J Gastroenterol 2012;47:607-13.

5. Muhi A, Ichikawa T, Motosugi U, et al. Mass-forming autoimmune pancreatitis and pancreatic carcinoma: differential diagnosis on the basis of computed tomography and magnetic resonance cholangiopancreatography, and diffusion-weighted imaging findings. J Magn Reson Imaging 2012;35:827-36.

### A3 Pancreatic cysts: where are we now?

#### G. Morana, A. Faccinetto, S. Venturini

##### Radiological Department, General Hospital Ca’ Foncello, Treviso, Italy

###### **Correspondence:** G. Morana

With advancements in diagnostic imaging, cystic lesions of the pancreas (PCLs) are being detected with increased frequency. As cystic tumours require a different treatment according to their histological type and differentiation, a correct diagnosis is important; however as specific clinical and laboratoristic signs usually are not present, the overlap of imaging findings between different cystic tumours makes the management of these lesions complex. PCLs may be simply classified into two main group, non-neoplastic and neoplastic cysts, more commonly defined as pancreatic cystic neoplasms (PCNs). Neoplastic cysts can be divided in non-mucinous and mucinous, because the latter are considered premalignant lesions. Imaging features of CPLs have been extensively described, especially for serous cystic adenoma (1), Mucinous cystic neoplasms (2) and Intraductal papillary mucinous neoplasms (IPMNs) (3).

Several questions remain open: as the primary goal of the management of patients with premalignant pancreatic cysts is prevention of malignancy, while avoiding unnecessary surgery, it is not still clear which guideline better manage patients with IPMN: ia recent comparison of pathologically analysed resected cysts with the three main guidelines (International Association of Pancreatology – IAP-, the European Study Group on Cystic tumours of the Pancreas – ESG - and the American Gastroenterological Association – AGA -), found that although fewer patients should undergo unnecessary surgery based on the AGA guideline compared with the IAP and European guidelines, however, advanced neoplasia (HGD and adenocarcinoma) would have been missed in 12% of patients when the AGA guidelines were applied, in contrast to no misses with the IAP or European guidelines (4). In another paper the AGA guidelines missed 45% of IPMN patients with HGD or adenocarcinoma (5). Further studies are needed to decrease the rate of overtreatment that appears to be inevitable when applying the current guidelines, without missing malignancies.

Moreover, all guidelines are focused on the imaging features of pancreatic cysts, missing other comorbidities which may affect the medium-long term survival of patients with PCL. In a recent paper an analysis was conducted on of the survival of 1800 patients with CPLs, after classification of patients as low- (LP) or high-risk (HP) for comorbidities and of CPLs in low- (LC) or high-risk (HC) according to main pancreatic duct dilation (>5 mm) and cyst size >3cm, thus creating four groups: low-risk patient with low-risk cyst (LPLC), low-risk patients with high risk cyst (LPHC), high risk patient with low-risk cyst (HPLC) and high-risk patient with high-risk cyst (HPHC). Mortality for pancreatic cancer (PC) and for comorbidities (CM) were: LPLC (0.1% PC vs 8.1% CM), LPHC (7.2% PC vs 8.1% CM), HPLC (0.3% PC vs 38.8% CM) and HPHC (6.5% PC vs 46% CM) (6). These data suggest that a correct management of patients with CPL should take in count not only the imaging features of the lesions but also of the determining patient comorbidity to estimate potential morbidity and mortality from surgery (7).

Several validated comorbidity tools such as the Charlson Age Adjusted Comorbidity index (CACI) or an ACE-27 score are accurate at assessing life expectancy in a given patient population (8). In one study including a surveillance cohort of patients with presumed BD-IPMN, 10% of the patients had a high comorbidity CACI score of 7 or more, of which the median survival was 43 months. Of these patients 94% of their mortality was not related to IPMN, with a similar survival amongst patients who underwent surgery compared with those who were observed, concluding that CACI can be used to identify patients with a high risk of death from factors other than IPMNs within a few years after diagnosis, who are therefore not likely to benefit from further IPMN observation or pancreatic resection (9).

The duration of follow-up is another topic to be discussed, as there is not any agreement on the stopping of surveillance in low risk CPLs: while AGA guidelines suggest stopping surveillance after 5 years of cyst stability (10), another paper show a 18% patients under surveillance developed either WF or HRS during surveillance beyond 5 years (11).

Thus a combination of several features, either patient related (age, comorbidity state, preference) and lesion related (histology subtype, presence or absence of WF/HRS, duration of surveillance), are necessary to manage these patients, especially when stopping pancreatic cyst surveillance should be decided.

References

1. Choi J-Y, Kim M-J, Lee JY, Lim JS, Chung JJ, Kim KW, et al. Typical and atypical manifestations of serous cystadenoma of the pancreas: imaging findings with pathologic correlation. AJR Am J Roentgenol. 2009 Jul;193(1):136–42.

2. Buetow PC, Rao P, Thompson LD. From the Archives of the AFIP. Mucinous cystic neoplasms of the pancreas: radiologic-pathologic correlation. Radiogr Rev Publ Radiol Soc N Am Inc. 1998 Apr;18(2):433–49.

3. Tanaka M, Fernández-del Castillo C, Adsay V, Chari S, Falconi M, Jang J-Y, et al. International consensus guidelines 2012 for the management of IPMN and MCN of the pancreas. Pancreatol Off J Int Assoc Pancreatol IAP Al. 2012 Jun;12(3):183–97.

4. Lekkerkerker SJ, Besselink MG, Busch OR, Verheij J, Engelbrecht MR, Rauws EA, et al. Comparing 3 guidelines on the management of surgically removed pancreatic cysts with regard to pathological outcome. Gastrointest Endosc. 2017 May;85(5):1025–31.

5. Singhi AD, Zeh HJ, Brand RE, Nikiforova MN, Chennat JS, Fasanella KE, et al. American Gastroenterological Association guidelines are inaccurate in detecting pancreatic cysts with advanced neoplasia: a clinicopathologic study of 225 patients with supporting molecular data. Gastrointest Endosc. 2016 Jun;83(6):1107–1117.e2.

6. Kwok K, Chang J, Duan L, Huang BZ, Wu BU. Competing Risks for Mortality in Patients With Asymptomatic Pancreatic Cystic Neoplasms: Implications for Clinical Management. Am J Gastroenterol. 2017 Aug;112(8):1330–6.

7. Wilcox CM. Editorial: Management of the Small Asymptomatic Pancreatic Cyst: Somehow Along the Way We Forgot About the Patient. Am J Gastroenterol. 2017;112(8):1337–9.

8. Farrell JJ. Editorial: Stopping Pancreatic Cyst Surveillance? Am J Gastroenterol. 2017;112(7):1162–4.

9. Sahora K, Ferrone CR, Brugge WR, Morales-Oyarvide V, Warshaw AL, Lillemoe KD, et al. Effects of Comorbidities on Outcomes of Patients With Intraductal Papillary Mucinous Neoplasms. Clin Gastroenterol Hepatol Off Clin Pract J Am Gastroenterol Assoc. 2015 Oct;13(10):1816–23.

10. Vege SS, Ziring B, Jain R, Moayyedi P, Clinical Guidelines Committee, American Gastroenterology Association. American gastroenterological association institute guideline on the diagnosis and management of asymptomatic neoplastic pancreatic cysts. Gastroenterology. 2015 Apr;148(4):819-822; quize12-13.

11. Crippa S, Bassi C, Salvia R, Malleo G, Marchegiani G, Rebours V, et al. Low progression of intraductal papillary mucinous neoplasms with worrisome features and high-risk stigmata undergoing non-operative management: a mid-term follow-up analysis. Gut. 2017;66(3):495–506.

## 11:00 – 12:30 Screenings

### A4 Does screening have a real impact on mortality?

#### Manuel Zorzi

##### Veneto Tumour Registry, Azienda Zero, Padova, Italy

Many randomised controlled trials (RTC) were performed in the last forty years in order to define if breast-cancer specific mortality is significantly decreased in women exposed to mammographic screening, as compared to controls.

According to the WHO position paper published in 2014 [1], most mortality estimates from the eight available RTCs converge around a 20% relative risk reduction with mammography at 11 years of follow-up. However, the WHO Guideline Development Group (GDG) expressed serious concerns about indirectness of such evidence, as “trials had short follow-up, low participation rate and practice had probably significantly changed”. All these factors would underestimate the expected impact of ongoing population based screening programs. In fact, longer follow-up has demonstrated larger magnitudes of risk reduction, suggesting that the full impact of screening may require more than 20 years [2]. Further, a pooled analysis of the results of several observational studies (without any serious concern for indirectness) reported a risk reduction of 38% [1].

The WHO GDG emphasised the importance of evaluating mammography screening within organised population-based cancer screening programs. Several studies applied different designs in order to evaluate the impact of service screening programs on mortality: analysis of the incidence-based mortality (instead of the overall mortality); comparison of mortality before and after the introduction of screening; comparison of mortality trends in areas with and without an organised screening program, in areas with a different timing of introduction of screening, or in areas applying different screening policies. Most studies found an association between the introduction of screening and a mortality reduction ranging from 15% to 30% [3-6], with some exceptions (not-significant reduction by 11% in Norway [7]; limited impact of screening on mortality in Sweden [8]).

However, such observational studies are affected by several limitations that mainly produce a diluting effect of screening, such as the inclusion of deaths from cancers diagnosed before screening started or before women reached screening age, the phased build-up of screening, or the presence of opportunistic screening that took place before organised screening started. The latter seems particularly widespread in developed Countries, ranging from 40% in Norway [7] to 67% in Finland [9] and Italy [3].

In the presence of a decrease in mortality rates, better treatment is the obvious alternative candidate. However, efforts to disentangle the effects of organised screening, early diagnosis, and treatment are unlikely to be a reliable exercise, given the synergistic effect between them. Not only is mammography screening likely to detect breast cancers at earlier stages, allowing the adoption of less harmful and more effective treatments, but also the presence of an organised screening program may promote the provision of more effective care by monitoring the treatment quality of screen-detected cancers and by favoring the creation of multidisciplinary units of breast cancer specialists [10].

In the current scenario, priorities should be to increase the accuracy of breast cancer screening and the appropriateness and availability of treatment, to improve communication, and to advance research.

This abstract has been previously published.

References

1. WHO position paper on mammography screening. https://www.who.int/cancer/publications/mammography_screening/en/

2. Tabar L, Vitak B, Chen TH, et al. Swedish two-county trial: impact of mammographic screening on breast cancer mortality during 3 decades. Radiology. Sep 2011;260(3):658-663.

3. Capodaglio G, Zorzi M, Tognazzo S, et al. Impact of breast cancer screening in a population with high spontaneous coverage with mammography. Tumori 2018;104(4): 258-265.

4. Parvinen I, Heinävaara S, Anttila A, et al. Mammography screening in three Finnish residential areas: comprehensive population-based study of breast cancer incidence and incidence-based mortality 1976-2009. Br J Cancer 2015;112(5): 918-24.

5. Sankatsing VDV, van Ravesteyn NT, Heijnsdijk EAM, et al. The effect of population-based mammography screening in Dutch municipalities on breast cancer mortality: 20 years of follow-up. Int J Cancer 2017;141(4): 671-677.

6. Taylor R, Gregory M, Sexton K, et al. Breast cancer mortality and screening mammography in New Zealand: Incidence-based and aggregate analyses. J Med Screen 2019;26(1): 35-43.

7. Olsen AH, Lynge E, Njor SH, et al. Breast cancer mortality in Norway after the introduction of mammography screening. Int J Cancer 2013;132(1): 208-14.

8. Autier P, Koechlin A, Smans M, et al. Mammography screening and breast cancer mortality in Sweden. J Natl Cancer Inst 2012;104(14): 1080-93.

9. Heikkinen S, Miettinen J, Koskenvuo M, et al. Proportion of women with self-reported opportunistic mammography before organised screening. Acta Oncol 2016;55(7): 865-9.

10. Selby P, Gillis C, Howard R. Benefits from specialised cancer care. Lancet 1996;348(9023): 313–318.

### A5 High risk patients: genetic conditions for cancer: which surveillance?

#### Richard M. Gore, Daniel R. Wenzke, Kiran H. Thakrar, Silvers RI

##### Department of Radiology, North Shore University Health System-University of Chicago, Evanston, IL, USA

###### **Correspondence:** Richard M. Gore (rgore@uchicago.edu)

There is a broad spectrum of hereditary cancer syndromes which generally account for 5%–10% of malignancies. They are characterised by germline mutations of genes that lead to the early onset of distinctive tumour subtypes in specific organs. While these syndromes are rare, affected patients carry significantly elevated risks of developing cancer, as do their at-risk relatives. Identification of these patients is critical to ensure timely and appropriate genetic testing relevant to cancer patients and their relatives. Several guidelines and tools are available to assist clinicians

In this presentation, the cross-sectional imaging features of the major hereditary cancer syndromes including Lynch Syndrome, Familial Adenomatous Polyposis, Hereditary Diffuse Gastric Cancer Syndrome, MEN 1 and 2, Cowden Syndrome, Birt-Hogg-Dube, Tuberous Sclerosis, Li-Fraumeni Syndrome, von Hippel Lindau Syndrome, Hereditary Breast and Ovarian Cancer Syndrome are presented as well as the screening and surveillance recommendations for each of these disorders.

**References**

1. Tiwari R, Singh AK, Somwaru AS, Menias CO, Prasad SR, Katabathina VS: Radiologist's Primer on Imaging of Common Hereditary Cancer Syndromes. Radiographics. 2019; 39: 759-778.

2. Katabathina VS, Menias CO, Prasad SR. Imaging and Screening of Hereditary Cancer Syndromes. Radiol Clin North Am. 2017;55(6):1293-1309.

3. Ring KL, Modesitt SC: Hereditary Cancers in Gynecology: What Physicians Should Know About Genetic Testing, Screening, and Risk Reduction. Obstet Gynecol Clin North Am. 2018; 45:155-173.

4. Czarniecki M, Gautam R, Choyke PL, Turkbey B. Imaging findings of hereditary renal tumours, a review of what the radiologist should know. Eur J Radiol. 2018; 101:8-16.

5. Stoffel EM. Heritable Gastrointestinal Cancer Syndromes. Gastroenterol Clin North Am. 2016; 45:509-27.

6. Barnes CA, Krzywda E, Lahiff S, McDowell D, Christians KK, Knechtges P, Tolat P, Hohenwalter M, Dua K, Khan AH, Evans DB, Geurts J, Tsai S. Fam Cancer. 2018; 17:101-111.

### A6 Lung cancer screening after the results of the Nelson trial

#### R Vliegenthart

##### Dept of Radiology, University Medical Center Groningen, The Netherlands

Lung cancer is the leading cause of cancer-related mortality worldwide.(1) Despite improvements in lung cancer treatment, the survival of lung cancer patients is still poor. The survival primarily depends on the stage at the time of lung cancer diagnosis. Early detection based on low-dose computed tomography (CT), combined with early treatment, may reduce morbidity and mortality. The Early Lung Cancer Action Program (ELCAP) and National Lung Screening Trial (NLST), as well as the European lung cancer screening studies, have shown that most lung cancers detected in CT lung cancer screenees are stage I cancers,(3-10) in contrast to the situation in clinical detection when patients present with symptoms. This already gave an indication that low-dose chest CT could potentially improve survival. In the American NLST trial, annual low-dose chest CT in long-term smokers reduced lung cancer mortality by 20% compared to annual chest radiography.(2) This has led to recommendations for CT screening, in particular by US organisations. According to the US Preventive Services Task Force, individuals from 55 to 80 years who smoked for 30 pack-years and currently smoke or quit within 15 years are eligible for (opportunistic) lung cancer screening.(11) In different European countries, lung cancer screening trials have been performed or are ongoing, including in Italy, Germany, Belgium/Netherlands, Denmark and the UK (4-10). Until recently, none of the European studies with final mortality results showed a significant mortality benefit of CT screening. Potentially, the individual studies lacked sufficient statistical power to show mortality reduction. The Dutch-Belgian NELSON trial is the largest European study with nearly 16,000 lung cancer screening participants, randomised into low-dose CT screening or no screening. Recently, the 10-year mortality results of the NELSON trial were presented. The results strongly support the survival benefit of screening, with 26% lower lung cancer mortality in the male CT screening group, and a tendency towards an even larger mortality reduction in women.

In long-term smokers, lung nodules are present in about 50% of CT scans, the large majority being benign.(2,12) The challenge is to accurately and sensitively identify an early lung cancer without unnecessarily increasing anxiety, costs, and work-up. With regards to the nodule management protocol, there was an important difference between the NELSON trial and the NLST. In the NELSON trial, solid nodule evaluation was based on semi-automated volumetry at first detection, and volume-doubling time (VDT) on short-term follow-up CT in case of an indeterminate nodule.(4) In contrast, a cut-off based on manually measured nodule diameter, or growth in diameter, was used in the NLST trial. The NELSON approach led to much lower false positive rate for referral of screenees to the pulmonologist. Original volume/VDT criteria were optimised based on screening outcomes.(12) For new lung nodules on incident screening rounds, it was found that the nodule volume criteria for follow-up and referral have to be more stringent, in view of higher lung cancer probability of small incident lung nodules.(13) A recent consensus document has included these newer criteria in the recommendations.(14) In the presentation, an update will be provided on the current status of CT lung cancer screening.

References

1. Lozano R, Naghavi M, Foreman K, et al. Global and regional mortality from 235 causes of death for 20 age groups in 1990 and 2010: a systematic analysis for the Global Burden of Disease Study 2010. Lancet. 2012;380:2095-2128.

2. National Lung Screening Trial Research Team, Aberle DR, Adams AM, Berg CD, et al. Reduced lung-cancer mortality with low-dose computed tomographic screening. N Engl J Med. 2011;365:395-409.

3. International Early Lung Cancer Action Program Investigators, Henschke CI, Yankelevitz DF, et al. Survival of patients with stage I lung cancer detected on CT screening. N Engl J Med. 2006;355:1763-1771.

4. van Klaveren RJ, Oudkerk M, Prokop M, et al. Management of lung nodules detected by volume CT scanning. N Engl J Med. 2009;361:2221-2229.

5. Pastorino U, Rossi M, Rosato V, et al. Annual or biennial CT screening versus observation in heavy smokers: 5-year results of the MILD trial. Eur J Cancer Prev. 2012;21:308-315.

6. Infante M, Cavuto S, Lutman FR, et al. Long-term follow-up results of the DANTE trial, a randomized study of Lung cancer screening with Spiral computed tomography. Am J Respir Crit Care Med. 2015;191:1166-1175.

7. Becker N, Motsch E, Gross M-L, et al. Randomised Study on early detection of lung cancer with MSCT in Germany: results of the first 3 years of follow-up after randomisation. J Thorac Oncol. 2015;10:890-896.

8. Wille MMW, Dirksen A, Ashraf H, et al. Results of the randomised Danish lung cancer screening trial with focus on high-risk profiling. Am J Respir Crit Care Med. 2016;193:542-551.

9. Field JK, Duffy SW, Baldwin DR, et al. UK Lung Cancer RCT Pilot Screening Trial: baseline findings from the screening arm provide evidence for the potential implementation of lung cancer screening. Thorax. 2016;71:161-170.

10. Paci E, Puliti D, Lopes Pegna A, et al. Mortality, survival and incidence rates in the ITALUNG randomised lung cancer screening trial. Thorax. 2017;72:825-831.

11. Moyer VA. Screening for lung cancer: US Preventive Services Task Force Recommendation Statement. Ann Intern Med. 2014;160:330-338.

12. Horeweg N, van Rosmalen J, Heuvelmans MA, et al. Lung cancer probability in patients with CT-detected pulmonary nodules: a prespecified analysis of data from the NELSON trial of low-dose CT screening. Lancet Oncol. 2014;15:1332-1341.

13. Walter JE, Heuvelmans MA, de Jong PA, et al. Occurrence and lung cancer probability of new solid nodules at incidence screening with low-dose CT: analysis of data from the randomised, controlled NELSON trial. Lancet Oncol. 2016;17:907-916.

14. Oudkerk M, Devaraj A, Vliegenthart R, et al. European position statement on lung cancer screening. Lancet Oncol. 2017;18:e754-e766.

### A7 HCC screening and surveillance

#### Bachir Taouli

##### Icahn School of Medicine at Mount Sinai, New York, NY, USA

Hepatocellular carcinoma (HCC) is the 2^nd^ leading cause of cancer-related death worldwide, and the fastest growing cause of cancer death in the USA. The most important risk factor for HCC is cirrhosis. Recent practice guidelines recommend semi-annual HCC surveillance using ultrasound (US) with or without serum alpha-fetoprotein (AFP) for cirrhotic and other high-risk patients to permit detection of HCC at an early stage, enabling effective treatment, and potentially improving survival (1-9). The recommendation to use US with/without AFP for HCC screening and surveillance is based on a Chinese prospective randomised clinical trial, which found that semi-annual US+AFP screening reduced HCC mortality by 37% compared to no screening (10). Basing American guidelines on a Chinese study performed 20 years ago is problematic because the diagnostic performance of US screening may not translate to a Western at-risk population (1). Since the most important risk factor for HCC in China is HBV infection, not cirrhosis, the fraction of patients in the Chinese study with cirrhosis is unknown. By comparison, the livers of Americans with cirrhosis tend to be heterogeneous, which can obscure small masses (1). Also, Americans now are about five times more likely to be overweight or obese than Chinese in the 90s (11, 12). Due in part to the challenges imparted by cirrhosis and obesity, US sensitivity for early-stage, potentially curative disease is limited (as low as 47%) (1-9). Because of the low sensitivity of US for detecting HCC in cirrhotic patients, many centers instead perform multiphasic CE-CT or CE-MRI, which have reported per-patient HCC detection sensitivities ranging from 68%-81% (13). While they provide higher sensitivity than US. These methods are not optimal for screening (13, 14) because of higher cost, radiation exposure for CE-CT (13) and long exam times for CE-MRI (at least 30 min). As a result, American practice guidelines do not advocate multiphasic CE-CT or CE-MRI for HCC screening/surveillance. Novel abbreviated magnetic resonance imaging (AMRI) protocols including T1-weighted imaging (T1-wi) and T2wi at the hepatobiliary phase post gadoxetic acid injection (HBP-AMRI) (a liver specific contrast agent that permits early detection of HCC based on underexpression of organic anion transporting polypeptides) have been recently developed (15). These AMRI protocols are designed to detect HCC in cirrhotic patients with improved accuracy, in less than 10 min acquisition time. Recent data suggest that HBP-AMRI provides >80% sensitivity for HCC detection. In this presentation, we will discuss the performance of US and AFP for HCC screening and surveillance and we will review recent developments in the use of abbreviated MRI protocols for HCC screening and surveillance.

**References**

1. Kono Y, Joshi K, K R, M OB. Ultrasound In HCC Surveillance: What is the Quality of Ultrasound and What Factors Affect Quality? Annual Meeting of the American Institute for Ultrasound in Medicine (AIUM); Orlando, Florida2015.

2. Singal A, Volk ML, Waljee A, Salgia R, Higgins P, Rogers MA, Marrero JA. Meta-analysis: surveillance with ultrasound for early-stage hepatocellular carcinoma in patients with cirrhosis. Aliment Pharmacol Ther. 2009;30(1):37-47. Epub 2009/04/28. doi: 10.1111/j.1365-2036.2009.04014.x. APT4014 [pii]. PubMed PMID: 19392863.

3. Bennett GL, Krinsky GA, Abitbol RJ, Kim SY, Theise ND, Teperman LW. Sonographic detection of hepatocellular carcinoma and dysplastic nodules in cirrhosis: correlation of pretransplantation sonography and liver explant pathology in 200 patients. AJR Am J Roentgenol. 2002;179(1):75-80. PubMed PMID: 12076908.

4. Kim CK, Lim JH, Lee WJ. Detection of hepatocellular carcinomas and dysplastic nodules in cirrhotic liver: accuracy of ultrasonography in transplant patients. J Ultrasound Med. 2001;20(2):99-104. PubMed PMID: 11211142.

5. Libbrecht L, Bielen D, Verslype C, Vanbeckevoort D, Pirenne J, Nevens F, Desmet V, Roskams T. Focal lesions in cirrhotic explant livers: pathological evaluation and accuracy of pretransplantation imaging examinations. Liver Transpl. 2002;8(9):749-61. PubMed PMID: 12200773.

6. Pateron D, Ganne N, Trinchet JC, Aurousseau MH, Mal F, Meicler C, Coderc E, Reboullet P, Beaugrand M. Prospective study of screening for hepatocellular carcinoma in Caucasian patients with cirrhosis. Journal of hepatology. 1994;20(1):65-71.

7. Saada J, Bhattacharya S, Dhillon AP, Dick R, Burroughs AK, Rolles K, Davidson BR. Detection of small hepatocellular carcinomas in cirrhotic livers using iodised oil computed tomography. Gut. 1997;41(3):404-7. PubMed PMID: 9378400; PMCID: PMC1891483.

8. Santagostino E, Colombo M, Rivi M, Rumi MG, Rocino A, Linari S, Mannucci PM, Centers SGotAoIH. A 6-month versus a 12-month surveillance for hepatocellular carcinoma in 559 hemophiliacs infected with the hepatitis C virus. Blood. 2003;102(1):78-82. doi: 10.1182/blood-2002-10-3310. PubMed PMID: 12649165.

9. Tradati F, Colombo M, Mannucci PM, Rumi MG, De Fazio C, Gamba G, Ciavarella N, Rocino A, Morfini M, Scaraggi A, Taioli E. A prospective multicenter study of hepatocellular carcinoma in italian hemophiliacs with chronic hepatitis C. The Study Group of the Association of Italian Hemophilia Centers. Blood. 1998;91(4):1173-7. PubMed PMID: 9454746.

10. Zhang BH, Yang BH, Tang ZY. Randomised controlled trial of screening for hepatocellular carcinoma. J Cancer Res Clin Oncol. 2004;130(7):417-22. Epub 2004/03/26. doi: 10.1007/s00432-004-0552-0. PubMed PMID: 15042359.

11. Wang Y, Mi J, Shan XY, Wang QJ, Ge KY. Is China facing an obesity epidemic and the consequences? The trends in obesity and chronic disease in China. Int J Obes (Lond). 2007;31(1):177-88. doi: 10.1038/sj.ijo.0803354. PubMed PMID: 16652128.

12. Ogden CL, Carroll MD, Kit BK, Flegal KM. Prevalence of Childhood and Adult Obesity in the United States, 2011-2012. JAMA. 2014;311(8):806-14.

13. Colli A, Fraquelli M, Casazza G, Massironi S, Colucci A, Conte D, Duca P. Accuracy of ultrasonography, spiral CT, magnetic resonance, and alpha-fetoprotein in diagnosing hepatocellular carcinoma: a systematic review. Am J Gastroenterol. 2006;101(3):513-23. PubMed PMID: 16542288.

14. Arguedas MR, Chen VK, Eloubeidi MA, Fallon MB. Screening for hepatocellular carcinoma in patients with hepatitis C cirrhosis: a cost-utility analysis. Am J Gastroenterol. 2003;98(3):679-90. PubMed PMID: 12650806.

15. Ueno A, Masugi Y, Yamazaki K, Komuta M, Effendi K, Tanami Y, Tsujikawa H, Tanimoto A, Okuda S, Itano O, Kitagawa Y, Kuribayashi S, Sakamoto M. OATP1B3 expression is strongly associated with Wnt/beta-catenin signalling and represents the transporter of gadoxetic acid in hepatocellular carcinoma. J Hepatol. 2014;61(5):1080-7. doi: 10.1016/j.jhep.2014.06.008. PubMed PMID: 24946283.

## 11:00 – 12:30 Structured Report

### A8 Structured report: the German solution

#### T. Persigehl

##### Department of Diagnostic and Interventional Radiology, University Hospital Cologne, Cologne, Germany

The radiological report is the key component in the communication between radiologists and referring clinicians^1^. Traditionally, reports are written as free texts. Several studies have shown that structured reporting using dedicated report templates has a number of advantages compared to conventional reports. Therefore, many radiological societies have recommended implementation of structured reporting in clinical routine.

In the meantime, collections of freely available templates have been presented and software solutions for structured reporting have been made commercially available. These allow for quality improvements in the written radiological report as they ensure all relevant clinical information to be included. However, most of these structured templates are in English. Thus, the German Radiological Society (DRG) has set it as its goal to develop consensus-based, quality-assured report templates in German which will be also translated in other languages.

Within this German “structured reporting” project first report templates have been developed and even more are in development in close cooperation with the respective committees of the DRG and referring clinicians using the Delphi method (https://www.onkologische-bildgebung.drg.de/).

Reference

1. Pinto Dos Santos D, Hempel JM, Mildenberger P, Klöckner R, Persigehl T. Structured Reporting in Clinical Routine. Rofo. 2018 Aug 13

### A9 Structured reporting: the American (my) perspective?

#### Isaac R Francis

##### University of Michigan (Michigan Medicine)**,** Ann Arbor, Michigan, USA

Advantages (Cons):

Structured reporting uses a checklist, thereby reducing “missed diagnosis”. They also include standard lexicon and disease-specific templates which help in improving the quality of reports, and key imaging findings, which are essential for treatment strategies such as in pancreatic and rectal carcinoma staging [Refs1-5]. Structured reports also decrease the incidence of grammatical and non-grammatical errors. Complete documentation also can cut reimbursement losses [Ref.1-3].

Disadvantages and Cons:

Perceived negative impact on productivity, can be due to added time to change ingrained work habits. The decreased “eye dwell time” on the images can contribute to increased “missed findings”. Preloaded phrases on templates, that are not omitted from the reports as for example reporting that “no gallstones, or “Uterus: No masses”, when in fact, the patient has undergone a prior cholecystectomy or hysterectomy, can be problematic, due to patient confidence loss in the institution or health system [Refs.1-3].

Implementation:

Structured reporting is widely used in most academic radiology practices and is beginning to gain quick acceptance into the general radiology community.

Increased acceptance of templates can be achieved by getting input from end users, including trainees, as well as referring physicians and subspecialists. For more complex studies the input of subspecialty radiology and non- radiology society committees and endorsement of these templates by various major radiology and non-radiology subspecialties, via presentations by radiologists at non radiology subspecialty meetings and joint statement papers in their respective journals would be very helpful [Res.1-3,6,7].

Wider acceptance can also be anticipated in the future with buy-in from industry, with implementation of new software from PACS vendors, and voice recognition system software, automated launching of templates with study selection, quick-pick lists with drop down menus, as well as the ability to use free text within the structured template reports etc. [Refs.1-3,6,7].

Now, near future and future directions:

Other quality measures which are in progress include, follow up recommendations, critical report notifications, and incorporation of a lesion management tool to enable serial evaluation of lesion characteristics. Future directions include possibly radiation dose tracking, and interaction with decision making tools to guide radiologists to characterise lesions and enable a likely diagnosis as well as follow up recommendations [Refs.1-3].

Multi-media enhanced reports, with embedded images, and graphs, and correlation with other lab values including biomarkers, will likely soon be available, leading to a very comprehensive radiology report [Refs.1-3].

References

1. Ganeshan D, Duong PAT, Probyn L et al. Structured Reporting in Radiology. Academic Radiology 2018; 25: 66–73

2. European Society of Radiology eHealth and Informatics Subcommittee. ESR paper on structured reporting in radiology. Insights Imaging 2018: 9:1-7

3. Pinto dos Santos D et al. Structured Reporting in Clinical Routine. Fortschr Röntgenstr 2019

4. Sahni VA, Silveira PC, Sainani NI et al. Impact of a Structured Report Template on the Quality of MRI Reports for Rectal Cancer Staging. American journal of Roentgenology 2015; 205: 584–588

5. Brook OR, Brook A, Vollmer CM et al. Structured Reporting of Multiphasic CT for Pancreatic Cancer: Potential Effect on Staging and Surgical Planning. Radiology 2015; 274: 464–472

6. Larson DB, Towbin AJ, Pryor RM et al. Improving consistency in radiology reporting through the use of department-wide standardised structured reporting. Radiology 2013; 267: 240–250

7. Herts BR, Gandhi MS, Schneider E et al. How we do it: creating consistent structure and content in Abdominal Radiology Templates. 2019: 212:490-496

## 13:30 – 14:00 Keynote Lecture 1

### K1 Measuring the impact of imaging

#### Patrick M. Bossuyt

##### Amsterdam University Medical Centers, University of Amsterdam, The Netherlands

The introduction of new imaging modalities, or recommendations about their use for specific purposes, should be accompanied by solid evidence about the benefits they bring for patients and society. In that sense, imaging is not any different from laboratory medicine, and subject to the same considerations that apply for other healthcare interventions.[1] Most systems in Europe and the US for developing guidelines and for making decisions about reimbursement emphasise (comparative) effectiveness.

There is a specific challenge for the imaging community when invited to provide evidence of benefit, as most forms of imaging do not carry an immediate benefit for patients or society. Only the appropriate use of the radiologists’ readings will generate the desired improvement in patient outcomes or health care efficiency. This largely indirect relation between imaging and outcomes requires for creative and alternative approaches for documenting the impact of imaging.[2]

In historical terms, the introduction of CT imaging in the 1970s was a milestone in the evaluation of imaging. Radiologists were concerned that payers would only be willing to invest in the highly expensive new equipment if they could provide evidence of efficacy. This eventually inspired Fineberg, Fryback and Thornbury to develop a hierarchical system for evaluating the effectiveness of diagnostic imaging.[3-4]

Direct evidence of the effects of imaging on patient outcomes is rare, but it does exist. Randomised trials of imaging with patient-based outcomes have been designed, and not only in population screening programs.[5] More common, however, are linked evidence approaches, in which information on the clinical performance of diagnostic imaging (detection/sensitivity/specificity) are combined with evidence of the effectiveness of downstream actions, guided by imaging results.

The recent emphasis on value-added healthcare has also rekindled efforts to document the benefits that imaging brings to patients, physicians, and payers. Attempts have been made to make reimbursement commensurate with adherence to new metrics that should express radiology’s contribution to outcomes and costs.[5]

In this presentation, we will start with a historical perspective on attempts to measure and express the impact of imaging. We will then provide a framework that highlights the differences between process and outcome, and between outcomes, effectiveness and efficiency. We will then offer a critical perspective on some of the metrics to express impact.

## Afternoon Session: 14:00 – 15:30 - Characterisation: Head To Toe 1

### A10 Brain: tumours versus necrosis/infections

#### Giovanni Morana^1^, Antonia Ramaglia^2^, Andrea Rossi^1^

##### ^1^Neuroradiology Unit, IRCCS Istituto Giannina Gaslini, Genoa, Italy; ^2^Department of Radiology, IRCCS Fondazione Policlinico Universitario A. Gemelli, Rome, Italy

###### **Correspondence:** Giovanni Morana (giovannimorana@gaslini.org)

Differential diagnosis between brain tumours and tumour-like conditions is not always a straightforward process. Several non-neoplastic lesions can mimic brain neoplasms on neuroimaging. These include but are not limited to infections (abscesses/granulomas), radiation necrosis, vascular lesions (ischemic or haemorrhagic), inflammatory/demyelinating lesions as well as giant Virchow-Robin spaces [1-3].

Clinical data as well as follow-up imaging can add useful information in differentiating among these conditions. Misinterpretation may lead to a significant delay of adequate treatment of malignant tumours or may result in over-treatment of a tumour-like benign lesion.

Some peculiar conventional imaging findings (T1 hyperintensity, smooth rim of T2 hypointensity, and incomplete rim enhancement) can be extremely helpful to suggest a correct differential diagnosis. At the same time, diffusion weighted imaging (DWI) patterns (homogeneously reduced diffusion or leading-edge reduced diffusion) can also help in differentiating a bacterial abscess, fungal infection or a demyelinating lesion from a high-grade cystic necrotic neoplasm [4].

Additional advanced MRI modalities, such as magnetic resonance spectroscopy (MRS) and perfusion weighted imaging (PWI) may enhance diagnostic confidence through the estimation of the levels of various normal and abnormal metabolites and the evaluation of hemodynamic properties of brain tissue, thus overcoming some limitations of conventional MRI [5,6]. For example, high-grade brain neoplasms usually present with increased perfusion due to high microvascular density and neoangiogenesis, whereas most non-neoplastic lesions have lower blood volume.

The rational use and the most relevant applications of conventional and advanced imaging techniques in differentiating brain tumours from tumour-like conditions is the focus of the present work.

**References**

1. Bradley D, Rees J. Brain tumour mimics and chameleons. Pract Neurol. 2013, 13: 359-71.

2. Bosemani T, Poretti A. Tumour and Tumour like Masses in Pediatric Patients that Involve Multiple Spaces. Neuroimaging Clin N Am. 2017, 27:135-153.

3. Omuro AM, Leite CC, Mokhtari K, Delattre JY. Pitfalls in the diagnosis of brain tumours. Lancet Neurol. 2006, 5:937-48.

4. Starkey J, Li Y, Tihan T, Cha, S. Clinical Series: Five Simple MR Imaging Features to Identify Tumour Mimics. Neurographics, 2016, 6: 229-236.

5. Huisman TA. Tumour-like lesions of the brain. Cancer Imaging. 2009, 9: Spec No A:S10-3.

6. Cunliffe CH, Fischer I, Monoky D, Law M, Revercomb C, Elrich S, et al. Intracranial lesions mimicking neoplasms. Arch Pathol Lab Med. 2009, 133:101-23.

### A11 Mediastinal mass: characterisation

#### A. Chiti^1,2^ , M. Kirienko^1^, M. Sollini^1^

##### ^1^Humanitas University, Milan, Italy; ^2^Humanitas Research Hospital, Milan, Italy

###### **Correspondence:** A. Chiti (arturo.chiti@hunimed.eu)

A mediastinal mass can result from a multitude of malignant and benign conditions [1]. The most frequent entities of the anterior mediastinum are lymphoma and thymic neoplasms. However, the prevalence of the different diseases varies prominently with age and according to gender. In a recent consensus, the International Thymic Malignancy Interest Group (ITMIG) suggested a multidisciplinary diagnostic approach that comprises both clinical characteristics and medical imaging findings. At imaging, an anterior mediastinal mass can be identified without difficulties on a chest radiography; it usually appears as an extra soft tissue mass or opacity. Cross-sectional imaging is a second-level apprach to characterise the lesion. It allows to suppose a differential diagnosis, evaluate other abnormalities, and suggest the next step in patient management. Contrast-enhanced computed tomography (CT) is the modality of choice in the assessment of an anterior mediastinal mass. When evaluating a CT scan, location, size and morphology; heterogeneity, enhancement, presence of intralesional fat, cystic components, and calcification; involvement of adjacent structures can be evaluated [2]. However, in case of a cystic lesion, magnetic resonance imaging (MRI) is more appropriate. In fact, MRI has been shown to be superior to CT in differentiating cystic from solid masses such as thymic cysts from thymic neoplasms, identifying cystic/necrotic subvolumes within solid lesions, and distinguishing thymic hyperplasia from thymic neoplasms. ^18^F-FDG positron emission tomography (PET)/CT is not routinely performed to evaluate or characterise an anterior mediastinal mass. It plays role in staging and evaluating response in patients with particular malignant conditions. Accordingly, the lesions within the prevascular compartment could be distinguished by imaging alone (i.e., thyroid goiter, benign teratoma, cysts either pericardial or thymic, lipoma and thymolipoma, and thymic hyperplasia) or by combining imaging and clinical information (e.g., thymic hyperplasia, thymic epithelial neoplasms, lymphoma) [2]. Nonetheless, mediastinal mass appearance may not be straightforward or clinical presentation may be unusual resulting in a challenging diagnosis. Moreover, considering the multitude of entities arising in the mediastinum and their low incidence most imagers and clinicians may have difficulties in achieving a diagnosis [1]. Consequently, a mediastinal mass, with the exception of some well-circumscribed conditions requires histological assessment [1,3]. Moreover, in some contexts, biopsy is recommended to reach diagnosis before any treatment. The main risks in managing patients with a resectable mediastinal mass and uncertain diagnosis are surgical resection of lymphoma, which should be medically treated [4–6], or tumour seeding from an encapsulated malignancy during biopsy procedure [7,8]. Radiomics and machine learning methods have been proposed to extract data from medical images. Radiomics consists of calculation of a multitude of parameters, which capture the intensity distribution and texture of the lesions, to be tested for correlation with clinical and biological characteristics. Machine learning methods, comprising many different algorithms (generally using a supervised or semi-supervised approach), take medical images or image-derived parameters as inputs and provide a classification, clustering or prediction, in a data-driven manner. These image mining approaches have been used with promising results in many clinical settings [9], including mediastinal masses characterisation [10–14].

References

1. Carter BW, Okumura M, Detterbeck FC, Marom EM. Approaching the Patient with an Anterior Mediastinal Mass: A Guide for Radiologists. J Thorac Oncol. 2014;9:S110–8.

2. ITMIG Classification of Mediastinal Compartments and Multidisciplinary Approach to Mediastinal Masses 1.

3. Carter BW, Marom EM, Detterbeck FC. Approaching the Patient with an Anterior Mediastinal Mass: A Guide for Clinicians. J Thorac Oncol. 2014;9:S102–9.

4. Dreyling M, Thieblemont C, Gallamini A, Arcaini L, Campo E, Hermine O, et al. ESMO Consensus conferences: guidelines on malignant lymphoma. part 2: marginal zone lymphoma, mantle cell lymphoma, peripheral T-cell lymphoma. Ann Oncol Off J Eur Soc Med Oncol. 2013;24:857–77.

5. Eichenauer DA, Engert A, Andre M, Federico M, Illidge T, Hutchings M, et al. Hodgkin’s lymphoma: ESMO Clinical Practice Guidelines for diagnosis, treatment and follow-up. Ann Oncol. 2014;25:iii70–5.

6. Ghielmini M, Vitolo U, Kimby E, Montoto S, Walewski J, Pfreundschuh M, et al. ESMO Guidelines consensus conference on malignant lymphoma 2011 part 1: diffuse large B-cell lymphoma (DLBCL), follicular lymphoma (FL) and chronic lymphocytic leukemia (CLL). Ann Oncol Off J Eur Soc Med Oncol. 2013;24:561–76.

7. Kattach H, Hasan S, Clelland C, Pillai R. Seeding of stage I thymoma into the chest wall 12 years after needle biopsy. Ann Thorac Surg. 2005;79:323–4.

8. Nagasaka T, Nakashima N, Nunome H. Needle tract implantation of thymoma after transthoracic needle biopsy. J Clin Pathol. 1993;46:278–9.

9. Sollini M, Antunovic L, Chiti A, Kirienko M. Towards Clinical Application of Image Mining: A Systematic Review on Artificial Intelligence and Radiomics. Eur J Nucl Med Mol Imaging. 2019;

10. Yasaka K, Akai H, Abe O, Ohtomo K, Kiryu S. Quantitative computed tomography texture analyses for anterior mediastinal masses: Differentiation between solid masses and cysts. Eur J Radiol. 2018;100:85–91.

11. Yasaka K, Akai H, Nojima M, Shinozaki-Ushiku A, Fukayama M, Nakajima J, et al. Quantitative computed tomography texture analysis for estimating histological subtypes of thymic epithelial tumours. Eur J Radiol. 2017;92:84–92.

12. Iannarelli A, Sacconi B, Tomei F, Anile M, Longo F, Bezzi M, et al. Analysis of CT features and quantitative texture analysis in patients with thymic tumours: correlation with grading and staging. Radiol Med. 2018;123:345–50.

13. Lee HS, Oh JS, Park YS, Jang SJ, Choi IS, Ryu J-S. Differentiating the grades of thymic epithelial tumour malignancy using textural features of intratumoural heterogeneity via (18)F-FDG PET/CT. Ann Nucl Med. 2016;30:309–19.

14. Nakajo M, Jinguji M, Shinaji T, Nakajo M, Aoki M, Tani A, et al. Texture analysis of 18F-FDG PET/CT for grading thymic epithelial tumours: usefulness of combining SUV and texture parameters. Br J Radiol. 2018;91:20170546.

### A12 Chest: I find a nodule, what do I do?

#### C.J. Herold

##### Department of Biomedical Imaging and image-guided Therapy, Medical University of Vienna, Vienna, Austria

Pulmonary nodules are a common finding in daily clinical practice and occur in patients with or without none underlying diseases or disorders. In these patients, the role of imaging is to establish a diagnosis, to detect malignancy, to confirm benign disorders and thereby, to avoid unnecessary thoracotomy and finally, to limit the number of follow-up CT examinations. Performing these tasks can be supported by categorizing patients into different clinical scenarios. These include patients with known malignancy; individuals, who undergo lung cancer screening; symptomatic patients with fever, cough, malaise, etc.; and patients in whom nodules are found incidentally. Stratifying individuals into those categories will help to determine the individual pretest probability for having, a malignant nodule and subsequently, to apply management principles and guidelines.

**Patients with known malignancy**

In these patients, clinical symptoms may or may not be present. The role of CT is to detect or exclude focal abnormalities suggestive of pulmonary metastases. Management is commonly based on personalised decision-making in tumour boards. It is of note that up to 75 % of patients with known cancers show one or more nodules at CT examinations. Of these, up to 30 % are malignant, and up to 25 % are of metastatic origin (1,2,3). Pulmonary metastases are most common in sarcomas, melanomas, head and neck, thyroid and renal cancers as well as germ cell tumours. Patients with sarcomas are at the highest risk of having or developing pulmonary metastases. In these patients, a new lesion has a 80 % probability of representing metastasis, and there is also a high prevalence of metastases in small lesions less than 4 mm. In certain patients with a malignancy of the head and neck, oesophagus, lung, stomach, breast, cervix, biliary ducts, prostate and ovary, a new lesion is more likely to represent a second primary lung cancer than a metastatic lesion (2). Beyond metastases or second primaries, a new lesion in patients with known malignancies may also represent pseudoprogression of disease following or during immunotherapy, or benign disease such as COP or infection.

**Lung cancer screening**

Individuals undergoing lung cancer screening are by definition asymptomatic. CT will be performed to detect or exclude a focal lesion. Several management guidelines have been published, with ACR Lung-RADS^R^ 1.1 (4) being the most commonly used and most relevant for radiologists. Lung-RADS relates imaging findings to numeric categories. Lung-RADS^R^ 1.1 guidelines are now commonly integrated in institutional RIS and PACS systems, thus easifying the use in screening programs.

**Nodules in symptomatic patients**

Symptomatic patients may present with fever, cough, shortness of breath and systemic symptoms such as weakness or anemia. CT is commonly performed to detect or exclude abnormalities which may be the cause of the clinical symptomatology. No established guidelines for imaging and managing these patients exist, thus, management is case specific. Differential diagnoses of nodules and symptomatic patients include infection (bacteria, viruses, fungi) COP, focal eosinophil lung disease and nodules of malignant origin (lymphoma, Kaposi Sarcoma).

**Incidentally found nodules**

In these patients, imaging is commonly performed for symptoms unrelated to the presence or absence of a nodule. Nodules are commonly managed according to well established guidelines (revised Fleischner criteria, Gould criteria, etc.) (5,6). For radiologists, the Fleischner Society criteria are the most relevant and present revised recommendations for incidentally discovered lung nodules. They take into account NLST, Nelson, ELCAP, PanCan, and BCCA data, include refined clinical and morphologic risk factors and recognise the important role of physicians and patients preference for more conservative or more aggressive management.

**Summary**

The categorisation of the scenario in which a pulmonary nodule is detected helps in determining the pretest probability for a malignant nodule and aims at stratifying further management and subsequently, treatment.

References

1. Hanamiya M et al, Frequency and significance of pulmonary nodules on thin section CT in patients with extrapulmonary malignant neoplasms. Eur J Radiol. 2012, 81:152

2. Quint LE, et al, Solitary pulmonary nodules in patients with extrapulmonary neoplasms. Radiology. 2000;217:257

3. Callister MEJ, et al, British Thoracic Society Guidelines for the investigation and management of pulmonary nodules: accredited by NICE. BMJ 2015;70:Suppl.2

4. Martin MD et al, Lung-RADS: pushing the limits. RadioGraphics 2017;37:1975

5. MacMahon H., et al, Guidelines for management of incidental pulmonary nodules detected on CT-images: from the Fleischer Society 2017. Radiology 2017; 284:228

6. Bueno J, et al, Updated Fleischner Society Guidelines for managing incidental pulmonary nodules: common questions and challenging scenarios. RadioGraphics 2018; 38:1337

### A13 The oncological liver, not always METS

#### Khaled M. Elsayes

##### Department of Diagnostic Radiology, The University of Texas MD Anderson Cancer Center, Houston, Texas, USA 77030

A broad spectrum of focal lesions can involve the liver and represent a daily challenge in the clinical practice (1). These are usually detected and characterised contrast-enhanced computed tomography and magnetic resonance imaging which were proven to be reliable in the evaluation of various focal hepatic lesions. Metastatic deposit is the most common malignant pathology involving the liver which may present in various patterns and simulate other pathologies. Practical diagnostic approach has been proposed for the diagnosis of these lesions (2). Specifically, various enhancing lesions have been described in cirrhotic and non-cirrhotic livers. Vascular pathologies and variants can also mimic these neoplastic lesions (3) and will be also illustrated in this lecture.

This abstract has been previously published.

References

1. Elsayes KM, Leyendecker JR, Menias CO, et al. MRI characterisation of 124 CT-indeterminate focal hepatic lesions: evaluation of clinical utility. HPB (Oxford). 2007;9:208-15.

2. Elsayes KM, Narra VR, Yin Y, et al. Focal hepatic lesions: diagnostic value of enhancement pattern approach with contrast-enhanced 3D gradient-echo MR imaging. Radiographics. 2005;25:1299-320.

3. Elsayes KM, Shaaban AM, Rothan SM, A Comprehensive Approach to Hepatic Vascular Disease. Radiographics. 2017;37 (3):813-836.

## 16:00 – 17:30 Characterisation: Head To Toe 2

### A14 Retroperitoneum: there is a mass, what is it?

#### Isaac R Francis

##### University of Michigan (Michigan Medicine), Ann Arbor, Michigan, USA

**NEOPLASMS:**

1. Soft tissue masses:
  Sarcomas, lymphoma, lymph node metastases and extra gonadal germ cell tumours
2. Myxoid masses: (mixed soft tissue and low density)
  Neurogenic: Schwannoma, neurilemoma, ganglioneuroma, neurofibroma
3. Hypervascular masses:
  Paraganglioma, Castleman’s and other angiomatous tumours
4. Cystic masses:
  Lymphangiomas, mucinous tumours

**NON-NEOPLASMS:**

- Retroperitoneal fibrosis

Retroperitoneal Sarcomas:

The most common soft tissue sarcomas, are liposarcomas, with leiomyosarcomas being the next most common. [Refs.1.2]

Liposarcomas:

Atypical lipomatous tumours/well differentiated liposarcomas [ALT/WDLS], have imaging features that may overlap with that of benign lipomas. However malignant tumours usually measure > 10 cm in size, have thickened septa (measuring at least 2 mm), and have larger globular nodular solid enhancing foci of soft tissue. These tumours also show amplification of MDM2 and CDK4 positivity on FISH testing, whereas lipomas do not.

Leiomyosarcomas:

Retroperitoneal leiomyosarcomas may arise in the retroperitoneum and inferior vena cava (IVC) [Refs.1,2].

Lymphoma:

Non Hodgkin lymphoma involves extra-nodal and nodal sites, and tend to be hypovascular and usually demonstrate homogeneous enhancement. They tend to compress adjacent structures but vascular invasion and thrombosis are rare.

Metastatic lymph node enlargement:

Usually there is a known primary malignancy or one will be detected on imaging. Solid organ involvement is usually associated with lymph node enlargement enabling one to suggest the diagnosis.

Paragangliomas:

Paragangliomas arise from neural crest cells associated with autonomic ganglia. They can be associated with hereditary disorders such as von Hippel-Lindau disease(VHL), multiple endocrine neoplasia (MEN) sndromes among other [Ref.3].

On CT, and MRI paragangliomas appear as soft tissue attenuation masses that may show very brisk enhancement following intravenous contrast material administration.

Neuroblastomas/ganglioneuromas/ganglioneuroblastomas

Ganglioneuromas originate from the paravertebral sympathetic ganglia. They are seen as well-defined, homogeneous, low attenuation masses, with variable contrast enhancement, and low T1 and high T2 signal intensity on MRI [Ref.4].

Extragonadal germ cell tumours

Primary extra gonadal germ cell tumours of the retroperitoneum, have a varying appearance with seminomas presenting as homogeneous or heterogeneous soft tissue attenuation/signal intensity masses, teratomas often demonstrating fat, calcification and/or bone formation (seen only on CT), and with choriocarcinomas often being highly vascular and haemorrhagic [Ref.5].

Lymphangiomas:

These are rare benign congenital malformations, seen usually as thin walled multi-septated cystic lesions and may show foci of calcification [Refs.6,7].

Retroperitoneal fibrosis:

Seen usually as an ill-defined sheet-like region of abnormality surrounding the infra-renal abdominal aort and IVC, which can encase and obstruct the ureters. Rarely it can be “mass-like” and mimick a primary retroperitoneal malignancy [Ref.8].

**References**

1. Mizuki N, et al. Primary Retroperitoneal Neoplasms: CT and MR Imaging Findings with anatomic and pathologic diagnostic clues. Radiographics 2003:23:45-57.

2. Scali EP, et al. Primary retroperitoneal masses: what is the differential diagnosis? Abdom Imaging (2015) 40:1887–1903

3. Hayes WS, et al. Extra-adrenal retroperitoneal paraganglioma: clinical, pathologic, and CT findings. AJR Am J Roentgenol 1990;155(6):1247–50

4. Hughes MJ, Thomas JM, Fisher C, et al. Imaging features of retroperitoneal and pelvic schwannomas. Clin Radiol 2005;60(8):886–93.

5. Ueno T, et al. Spectrum of germ cell tumours: from head to toe. Radiographics 2004;24(2):387–404

6. Yang DM, et al. Retroperitoneal cystic masses: CT, clinical, and pathologic findings and literature review. Radiographics 2004;24(5): 1353–65.

7. Davidson AJ, Hartman DS. Lymphangioma of the retroperitoneum: CT and sonographic characteristic. Radiology 1990;175(2):507–10.

8. Vivas I, et al. Retroperitoneal fibrosis: typical and atypical manifestations. Br J Radiol 2000;73(866):214–22.

### A15 Differential Diagnosis of Renal Tumours

#### H. Chandarana

##### Department of Radiology, New York University School of Medicine, New York, USA

- Incidence of kidney cancer is steadily rising likely due to incidental detection of small renal masses.
- Nearly 20% of all small renal masses (less than 4cm in size) are benign at pathology. Furthermore, these small renal cancers represents 70% of all newly diagnosed Renal Cell Cancers (RCC).
- Detection of small renal masses leads to management dilemma, as it is not always possible to characterise these lesions on conventional imaging. Inability to discriminate benign from malignant and indolent from aggressive tumour results in surgical treatment for many of these patients.
- Surgery provides excellent oncologic control but it is associated with increased morbidity. Treatment based on tumour aggressiveness will result in optimal outcome by selecting patients with aggressive tumour for surgery and avoiding unnecessary surgery in indolent tumours. Such a paradigm requires non-invasive methods to accurately diagnose tumours of different aggressiveness.
- Tumours of different histopathology differ in tumour aggressiveness.
- Imaging can help investigate renal tumour histopathology and aggressiveness and can impact treatment decision and lower treatment cost.
- Imaging can assist with:

(A). Differentiating benign renal masses from malignant tumours.

- Protenacious or hemorrhagic cysts are hyperdense on CT and T1 hyperintense on MRI. Differentiating these lesions from solid masses requires assessment of enhancement. To assess for enhancement we can obtain pre-and post-contrast imaging on CT or MRI. Dual energy CT permits a single phase CT imaging to assess for enhancement, thus decreasing radiation dose. Subtraction imaging on MRI is useful to assess for enhancement.
- Benign angiomyolipoma (AML) contain bulk fat. This can be easily diagnosed on CT and MRI.
- Lipid poor AML can be difficult to differentiate from other types of renal masses on CT imaging. MRI can be helpful in suggesting the diagnosis of lipid poor AML. Some of the MRI features that suggest diagnosis of AML include homogenous and uniform enhancement, homogenous T2 signal similar to that of muscle, and restricted diffusion with low ADC. Suggesting this diagnosis is important as diagnosis of lipid poor AML can be made confidently at core biopsy, thus avoiding surgery in these patients
- It is nearly impossible to discriminate benign oncocytoma from chromophobe and clear cell subtypes of kidney cancers on conventional imaging. However, diffusion weighted imaging (DWI) and perfusion weighted imaging (PWI) has shown some promise in small pilot studies.

(B). Tumour subtypes of solid RCC

- Clear cell RCC are more aggressive compared to papillary and chromophobe subtype of RCC. These three subtypes account for over 90% of all RCC. Conventional CT and MR demonstrate increased enhancement in clear cell RCC compared to other subtypes of tumours but there is considerable overlap. On the other hand, papillary subtype of RCC is hypovascular compared to other subtypes. Furthermore, homogenous low T2 signal, T1 hyperintensity, and low ADC values can also suggest diagnosis of papillary RCC.
- Advance DWI and PWI may further improve accuracy of MRI in discriminating various subtype of kidney cancers and has shown potential in discriminating chromophobe RCC from other subtypes.

**References**

1. Thompson RH, Kurta JM, Kaag M, et al. Tumour size is associated with malignant potential in renal cell carcinoma. J Urol 2009;181(5):2033–6.

2. Hindman N, Ngo L, Genega EM, et al. Angiomyolipoma with minimal fat: can it be differentiated from clear cell renal cell carcinoma by using standard MR techniques? Radiology. 2012 Nov;265(2):468-77.

3. Sasiwimonphan K, Takahashi N, Leibovich BC, Carter RE, Atwell TD, Kawashima A. Small (<4 cm) renal mass: differentiation of angiomyolipoma without visible fat from renal cell carcinoma utilizing MR imaging. Radiology. 2012 Apr;263(1):160-8.

4. Rosenkrantz AB, Hindman N, Fitzgerald EF, Niver BE, Melamed J, Babb JS. MRI features of renal oncocytoma and chromophobe renal cell carcinoma. AJR Am J Roentgenol. 2010 Dec;195(6):W421-7.

5. Chandarana H, Rosenkrantz AB, Mussi TC, et al. Histogram analysis of whole-lesion enhancement in differentiating clear cell from papillary subtype of renal cell cancer. Radiology. 2012 Dec;265(3):790-8.

### A16 Uterus: fibroid vs sarcoma

#### Gigin Lin (giginlin@cgmh.org.tw)

##### Department of Medical Imaging and Intervention, Chang Gung Memorial Hospital, Taipei, Taiwan

Benign uterine fibroids share similar clinical presentations include abnormal vaginal bleeding, palpable pelvic mass, and occasionally pelvic pain, leading to approximately 0.5% of resected tumours with a preoperative diagnosis of fibroids are unexpectedly revealed to be sarcoma based on final histopathology. Preoperative imaging diagnosis of uterine sarcoma is increasingly important due to emerging non-hysterectomy options for symptomatic leiomyoma, for instance, laparoscopic myomectomy, uterine artery embolisation, or high intensity focused ultrasound. Because the myometrial location of tumours renders tissue diagnosis from endometrial samplings extremely difficult, preoperative imaging diagnosis plays a crucial role in tumour characterisation. Central non-enhancement on MRI should raise the suspicion of leiomyosarcoma (LMS) or smooth muscle tumour with uncertain malignant potential (STUMP) against benign fibroids [1]. Uterine carcinosarcoma (UCS), also known as the malignant mixed mesodermal tumour or malignant mixed Müllerian tumour (MMMT), most commonly present as an endometrial mass with a prolonged enhancement that distends the endometrial cavity and endocervical canal, mimicking a myoma delivery [2]. Endometrial stromal sarcoma (ESS) typically shows intratumoral T2 hypointense bands, marginal nodules, intramyometrial worm-like nodular extensions, which help to distinguish ESS from leiomyoma or adenomyosis on MRI. Feather-like enhancement is indicative of high-grade ESS with a poor prognosis [3]. The multi-septated cystic areas with a lattice-like appearance on MRI is suggestive of adenosarcoma (AS), which should not be confused with benign adenofibroma or endometrial polyp. Conversant with classical imaging characteristics and emerging technologies including artificial intelligence will continuously improve the management of uterine tumour [4].

References

1. Lin G, Yang LY, Huang YT, Ng KK, Ng SH, Ueng SH, et al. Comparison of the diagnostic accuracy of contrast-enhanced MRI and diffusion-weighted MRI in the differentiation between uterine leiomyosarcoma / smooth muscle tumour with uncertain malignant potential and benign leiomyoma. J Magn Reson Imaging. 2016;43:333-42.

2. Huang YT, Chang CB, Yeh CJ, Lin G, Huang HJ, Wang CC, et al. Diagnostic accuracy of 3.0T diffusion-weighted MRI for patients with uterine carcinosarcoma: Assessment of tumour extent and lymphatic metastasis. J Magn Reson Imaging. 2018;48:622-631.

3. Huang YL, Ueng SH, Chen K, Huang YT, Lu HY, Ng KK, et al. Utility of Diffusion-Weighted and Contrast-Enhanced Magnetic Resonance Imaging in Diagnosing and Differentiating Between High- and Low-Grade Uterine Endometrial Stromal Sarcoma. Cancer Imaging (in press)

4. Huang YT, Huang YL, Ng KK, Lin G. Current Status of Magnetic Resonance Imaging in Patients with Malignant Uterine Neoplasms: A Review. Korean J Radiol. 2019;20:18-33.

### A17 I see a lesion in the testis: what is it?

#### Michele Bertolotto (bertolot@units.it)

##### Department of Radiology, University of Trieste. Ospedale di Cattinara, Strada di Fiume 449, 34149 Trieste, IT

Ultrasonography has a nearly absolute sensitivity in detecting testicular lesions and in differentiating intra- vs. extra-testicular pathologies, but specificity is low. Many solid lesions have no special ultrasonographic character [1]. Laboratory tests can be useful, as elevated tumour markers testify malignancy, but normal markers do not rule out testicular neoplasms [2]. Assessment of lesion vascularity is of limited help.

In practice, we are often unable to characterise incidentally detected testicular lesions, to assess what their clinical relevance is, and how to manage them. This is an ever-growing problem with the increasing number of ultrasound investigations performed during the urologic workup. An increasingly large number of small, asymptomatic testicular lesions is incidentally detected [3–6].

The current urological guidelines suggest an aggressive approach to these lesions, with inguinal exploration in all patients, and orchidectomy for malignant tumours, or enucleation with frozen section histological examination if diagnosis is not clear [2]. Small incidentally detected testicular lesions, however, are benign in up to 68% of cases, and many are non-tumour nodules. Among them, granuloma, focal orchitis, abscess, infarction, fibrous pseudotumour, and hematoma can be difficult to differentiate from tumours [6–8].

Clinical correlation is vital: many non-neoplastic conditions likely manifest with acute scrotum [7]. One needs to be cautious, however, because also tumours can occasionally manifest with pain. Also, history of fever or trauma may suggest a non-neoplastic origin, permitting conservative management. In any case, the ultrasound findings of traumatic and inflammatory changes evolve rapidly; if a non-neoplastic intratesticular pathology is suspected, a short-term follow-up ultrasonographic examination allows differential diagnosis with tumour.

Technical advances in US, such as elastography and CEUS, can help tissue characterisation but often differentiation between benign and malignant lesions remains problematic [9, 10]. Further evaluation with MR may be helpful in selected cases to characterise lipomas, haematomas, and fibrous pseudotumours.

Urologists and radiologists are now fully aware that small, incidentally detected testicular lesions are overtreated and that a more conservative approach is necessary. Immediate orchidectomy should be avoided, and active surveillance with US should be performed every three months in the first year and then annually [5]. Surgery should be considered only for lesions that show increasing volume at follow-up. Testicular sparing surgery can be used, with removal of the lesion only, frozen-section analysis of the specimen and decision on orchidectomy (or not) based on the results provided by the pathologist.

Close cooperation among different specialists is needed in this field [5]. A multidisciplinary “testis unit” in which urologists, radiologists and pathologists work together on these patients and learn how to choose the best approach to each of them is the likely solution.

According with Scandura et al. a patient who undergoes orchidectomy for an incidentally discovered small testicular nodule which turns out to be benign is a “victim of modern imaging technology [6]”. However, He is more likely the victim of our misunderstanding of the meaning of what technology shows us and of the adherence to reactionary, outdated surgical dogma to the focal intra-testicular lesion.

**References**

1. Tsili AC, Bertolotto M, Rocher L et al. Sonographically indeterminate scrotal masses: how MRI helps in characterisation. Diagn Interv Radiol 2018; 24: 225-236.

2. Laguna MP, Albers P, Albrecht W et al. EAU Guidelines on Testicular Cancer. 2019;

3. Carmignani L, Gadda F, Gazzano G et al. High incidence of benign testicular neoplasms diagnosed by ultrasound. J Urol 2003; 170: 1783-1786.

4. Giannarini G, Dieckmann KP, Albers P, Heidenreich A, Pizzocaro G. Organ-sparing surgery for adult testicular tumours: a systematic review of the literature. Eur Urol 2010; 57: 780-790.

5. Rocher L, Ramchandani P, Belfield J et al. Incidentally detected non-palpable testicular tumours in adults at scrotal ultrasound: impact of radiological findings on management Radiologic review and recommendations of the ESUR scrotal imaging subcommittee. Eur Radiol 2016; 26: 2268-2278.

6. Scandura G, Verrill C, Protheroe A et al. Incidentally detected testicular lesions <10 mm in diameter: can orchidectomy be avoided. BJU Int 2018; 121: 575-582.

7. Bertolotto M, Derchi LE, Sidhu PS et al. Acute segmental testicular infarction at contrast-enhanced ultrasound: early features and changes during follow-up. AJR Am J Roentgenol 2011; 196: 834-841.

8. Valentino M, Bertolotto M, Martino P, Barozzi L, Pavlica P. Incidentally detection of non-palpable testicular nodules at scrotal ultrasound: what is new. Arch Ital Urol Androl 2014; 86: 378-382.

9. Sidhu PS, Cantisani V, Dietrich CF et al. The EFSUMB Guidelines and Recommendations for the Clinical Practice of Contrast-Enhanced Ultrasound (CEUS) in Non-Hepatic Applications: Update 2017 (Long Version). Ultraschall Med 2018; 39: e2-e44.

10. Sidhu PS, Cantisani V, Dietrich CF et al. The EFSUMB Guidelines and Recommendations for the Clinical Practice of Contrast-Enhanced Ultrasound (CEUS) in Non-Hepatic Applications: Update 2017 (Short Version). Ultraschall Med 2018; 39: 154-180.

## 16:00 – 17:30 Percutaneous Interventions

### A18 Percutaneous interventions: Lung – CT-guided Microwave ablation

#### S. Diederich (stefan.diederich@vkkd-kliniken.de)

##### Department of Diagnostic and Interventional Radiology, Düsseldorf, Germany

In this presentation I present a personal approach to lung MWA.

Microwave ablation (MWA) as well as other techniques of thermal ablation in primary lung cancer and pulmonary metastases is usually prescribed by multidisciplinary team decisions taking into account alternative therapies such as video-assisted thoracoscopic surgery (VATS) and stereotactic radiotherapy [Mouli, Ujiie].

Aspects that favour MWA over VATS include centrally located lesions in which MWA may allow better preservation of lung parenchyma compared to resection, the presence of pleural adhesions and (functional) single lung.

Aspects that favour MWA over stereotactic radiation include previous radiotherapy to the involved part of the chest and patients too uncooperative for radiotherapy.

Although MWA can be performed in conscious sedation we prefer therapy in general anaesthesia with double lumen intubation for better control of respiration and potential complications.

Contraindications include coagulation disorders, pulmonary hypertension, COPD, particularly paraseptal or bullous emphysema.

The patient is positioned in the CT scanner in a position that avoids puncture of vessels, bronchi and fissures and a needle position with sufficient distance of the active part of the microwave electrode from the pleura. The MWA probe is either advanced centrally through the target lesion or placed immediately adjacent to the lesion in different positions. The needle tip is placed distally to the target lesion. Energy and duration of the treatment is chosen according to the size and position of the lesion.

The therapy is monitored with intermittent CT imaging. Successful MWA is demonstrated by a halo of ground-glass density and/or consolidation surrounding the target lesion completely with a safety margin of at least 5 mm.

Tract ablation with reduced energy is performed during removal of the MWA probe to avoid haemorrhage and tumour seeding.

The most common immediate complication is pneumothorax which - if required - can be treated in the same session with aspiration or drainage. Haemorrhage is less common and is usually self-limited. It may require prolonged (double lumen) intubation, positioning with the involved lung in the dependent position and substitution of blood products.

CT follow-up on the next day ideally shows ground glass density completely surrounding the target lesion with a peripheral zone of consolidation.

Late complication include lung abscess and pleural empyema which may require antibiotic, percutaneous or surgical intervention.

The technical success rate depends on the lesion size (< 3 cm, > 3 cm), number and position of lesions [Healy]. If local recurrence occurs it can usually be treated with repeat MWA.

**References**

1. Mouli SK, Kurilova I, Sofocleous CT,^,^ Lewandowski RJ. The Role of Percutaneous Image-Guided Thermal Ablation for the Treatment of Pulmonary Malignancies. AJR Am J Roentgenol. 2017; 209: 740-751

2. Ujiie H, Yasufuku K. Understanding the possibility of image-guided thermal ablation for pulmonary malignancies. J Thorac Dis. 2018; 10: 603–609

3. Healey TT, March BT, Baird G, Dupuy DE. Microwave Ablation for Lung Neoplasms: A Retrospective Analysis of Long-Term Results. J Vasc Interv Radiol. 2017; 28: 206-211

## Tuesday 8^th^ October - Morning Session: 09:00 – 10:30 Artificial Intelligence In Oncological Imaging

### A19 Attitudes and perception of artificial intelligence and machine learning in oncological imaging

#### Dow-Mu Koh

##### Royal Marsden Hospital, United Kingdom

Artificial intelligence (AI) and machine learning are potential disruptors of imaging studies, which are likely to affect how radiologists and allied healthcare professionals work in the future. However, as these technologies are not embodied onto any single machine, many radiologists are unaware or alienated from developments in the field. In addition, it is also more difficult for radiologists to learn about these techniques, as the language of engagement is through mathematics and informatics, which are unfamiliar to most radiologists.

We undertook an online international survey to understand the current attitudes and perceptions of radiologists to AI and machine learning in cancer imaging, resulting in 664 responses from radiologists across more than 40 countries. Responders came from all practice backgrounds, across wide age ranges, and higher response rates from men (62%) than from women (38%).

More than 66% of the responders indicated that the benefits of AI and machine learning are much bigger or slightly bigger than the risks for cancer imaging. In addition, more than 86% of responders felt that AI tools would be used in at least some areas of work that would add value to cancer imaging within the next 5 years.

The participants had good agreement with the perceived positive effects of utilising AI and machine learning; but there was more disagreement about the possible negative effects such as whether these technologies would replace radiologists, whether radiologist’s workings would be dictated by machines or whether AI will devalue the work of radiologist. However, overall, there was a high level of agreement (>86%) that radiologists should engage in more direct communication/ consultations with patients. The majority of radiologist would also like early engagement with stakeholders and vendors in the field; as well as to prepare their departments now for these evolving technologies.

The responders to the survey indicated the importance of the following developments: (1) Tools that automates tracking of tumours across multiple imaging time points and assess their response to treatment; (2) Tools that improve automatic or semi-automatic tumour segmentation for different anatomical sites/ cancer type; (3) Tools that support radiologists in proforma reporting allowing annotated imaging data to be captured prospectively; (4) Tools that help to confidently identify normal studies so that radiologists can focus on dealing with the abnormal examinations; and (5) Tools that help to identify tumours across the body.

**Reference**

1. Bi WL, Hosny A, Schabath MB, Giger ML, Birkbak NJ, Mehrtash A, Allison T, Arnaout O, Abbosh C, Dunn IF, Mak RH, Tamimi RM, Tempany CM, Swanton C, Hoffmann U, Schwartz LH, Gillies RJ, Huang RY, Aerts HJWL. Artificial intelligence in cancer imaging: Clinical challenges and applications. CA Cancer J Clin. 2019 Mar;69(2):127-157.

### A20 Road map for development of artificial intelligence in oncological imaging

#### F. Prior (FWPrior@uams.edu)

##### Department of Biomedical Informatics, University of Arkansas for Medical Sciences, Little Rock, Arkansas 72205, USA

Artificial Intelligence (AI) and machine learning (ML) are not new to medicine or medical imaging. There is a substantial literature dealing with the application of machine learning techniques in medical imaging beginning in the 1980s. Known as Computer Aided Detection/Diagnosis (CAD), research in this field led to the development of key deep learning algorithms in the 1990s and the application of these technologies in commercial products[1]. In spite of years of research and development, the number of clinically successful CAD products with FDA approval has been rather limited, until recently [2]. What has changed to cause a resurgence in interest in AI in medical imaging? While the success of applications such as IBM’s Watson [3] figure heavily in the media, readily available computing power, a wide array of available software tools, large quantities of open access data and the emergence of radiomics facilitate development and drive research interest [4].

Despite recent concerns that “Radiology is Going Away…” [5] , robots are not going to replace radiologists[6]. However, AI will change all medical practice, including radiology. How will these changes come about? Perhaps we can draw on the history of Picture Archive and Communication Systems (PACS) for a possible roadmap. Initially there was resistance to PACS adoption, but as the technology evolved and adoption increased, automation led to changes in work patterns and improvements in efficiency. Today PACS is the norm in radiology departments world-wide, driven in large party by the rapid advancement of internet technologies that made digital imaging a global norm. Likely the same will be true with AI. PACS workstations will get smarter, image quality will improve, workflows will be simplified, new screening tools will handle the easy cases reducing workload [7], cognitive assistants [8] will help with scheduling, literature searches and much more. These advances will find their way into radiology practice, largely paralleling changes in everyday life – smart cars, the internet of things, ubiquitous digital assistants.

Perhaps as important as changes in clinical practice, ML techniques are opening new avenues for research. The ability to find new patterns in data, to identify new image features of significance to cancer diagnosis, and precise phenotypes to inform precise therapies will exert an increasing influence on cancer research [9].

As with many technology trends, AI applications in cancer imaging are currently experiencing a period of rapid growth and somewhat overheated speculation. However, the techniques are technically sound and productive applications will evolve and have a profound, positive impact on clinical practice.

References

1. Giger ML, Chan HP, Boone J. Anniversary paper: History and status of CAD and quantitative image analysis: the role of Medical Physics and AAPM. Medical physics. 2008;35(12):5799-820.

2. Topol EJ. High-performance medicine: the convergence of human and artificial intelligence. Nature medicine. 2019;25(1):44.

3. Bluemke DA. Radiology in 2018: Are You Working with AI or Being Replaced by AI? Radiology. 2018;287(2):365-6.

4. Prior F, Almeida J, Kathiravelu P, Kurc T, Smith K, Fitzgerald T, et al. Open access image repositories: high-quality data to enable machine learning research. Clinical radiology. 2019.

5. Moehrle A. “Radiology” Is Going Away... and That's Okay: Titles Change, A Profession Evolves. Journal of the American College of Radiology: JACR. 2018;15(3 Pt B):499-500.

6. Chan S, Siegel EL. Will machine learning end the viability of radiology as a thriving medical specialty? The British journal of radiology. 2018;91(xxxx):20180416.

7. Thrall JH, Li X, Li Q, Cruz C, Do S, Dreyer K, et al. Artificial intelligence and machine learning in radiology: opportunities, challenges, pitfalls, and criteria for success. Journal of the American College of Radiology. 2018;15(3):504-8.

8. Syeda-Mahmood T. Role of big data and machine learning in diagnostic decision support in radiology. Journal of the American College of Radiology. 2018;15(3):569-76.

9. Bi WL, Hosny A, Schabath MB, Giger ML, Birkbak NJ, Mehrtash A, et al. Artificial intelligence in cancer imaging: Clinical challenges and applications. CA: a cancer journal for clinicians. 2019;69:127-57.

## 11:30 – 13:00 Staging Head to Toe – What Surgeons Need to Know

### A21 Nasopharyngeal carcinoma: what surgeons need to know

#### A.D. King (King2015@cuhk.edu.hk)

##### Chinese University of Hong Kong, Hong Kong, PRC

**Introduction**

Nasopharyngeal carcinoma (NPC) is treated by radiotherapy for early-stage disease and chemoradiotherapy for advanced-stage disease. Therefore, the role of surgery is primarily for the initial detection of nasopharyngeal carcinoma and salvage surgery for residual/recurrent disease in the head and neck. Surgery is occasionally used also for selected patients with distant metastases, such as those with oligo metastases in the lungs, or for radiotherapy complications such as osteoradionecrosis, radiation injury in the brain, carotid blowout and radiation induced tumours. This lecture will focus on what surgeons need to know for NPC detection and for salvage surgery in the head and neck.

**NPC detection**

Nasopharyngeal endoscopic examination and biopsy for histological confirmation of a primary tumour is the investigation of choice for the diagnosis of NPC. However, compared to the rest of the aerodigestive tract the nasopharynx is a more challenging region for endoscopic examination because tumours may be hidden from view in the submucosa, pharyngeal recess or adenoid. Therefore, MRI has a complimentary role in the detection of these tumours. MRI has a high sensitivity (91%-100%) for NPC detection and can detect the 10-12% of tumours that are invisible on the endoscopic examination. This figure rises to 17% of NPCs identified from a recent population plasma EDV-DNA screening study in which there was a relatively high percentage of early-stage tumours compared to the historical data (71% vs 20%). Therefore, as more patients with suspected early-stage disease are subject to investigation, MRI will have an increasing role in the detection of this cancer. The MRI guideline for detection using a five-grade system will be illustrated in the lecture.

**Salvage surgery for residual/recurrent disease**

A multimodality approach based on MRI (including T2 and diffusion-weighted imaging) and FDG PET/CT is used to detect recurrence and map the extent of disease and relationship to vital structures.

Primary tumour relapse occurs in approximately 10% of NPC patients, and imaging plays an important role in detection because one third are submucosal and cannot be identified by endoscopic examination. Assessment of the extent of local recurrence on MRI also is essential for guiding surgical management and distinguishing the tumour boundary from post-radiotherapy changes. In the past nasopharyngectomy was reserved for early-stage primary recurrences and so involvement of the skull base (unless minor), internal carotid artery, brain and cavernous sinus were contraindications to nasopharyngectomy. However, with recent advancements in endoscopic surgery wider local excisions are being performed in specialised centres.

Nodal relapse occurs in approximately 5% of NPC patients and is often associated with local relapse, so it is important to scrutinise the primary tumour bed on imaging. Recurrent cervical nodal metastases are frequently found in the upper neck at level II and may not form a discrete node because they arise within the scar tissue of a previously treated metastatic node. Nodal relapse often involves multiple nodes at more than one level. Therefore, when planning a neck dissection all sites of nodal disease in both sides of the neck should be identified. This often requires both PET/CT and MRI (+/- ultrasound and FNAC for indeterminate nodes), while MRI is used to map the relationship of nodes with extranodal spread to vital structures including the carotid and vertebral vessels.

References

1. King AD, Vlantis AC. Bhatia KSS, et al. Primary nasopharyngeal carcinoma: Diagnostic accuracy of MR imaging versus that of endoscopy and endoscopic biopsy. Radiology 2011, 258: 531-537.

2. King AD, Woo JKS, Ai, Q-Y, et al. Complementary roles of MRI and endoscopic examination in the early detection of nasopharyngeal carcinoma. Annals of Oncology 2019, Epub 2019.

3. Shayah A, Wickstone L, Kershaw E, et al. The role of cross-sectional imaging in suspected nasopharyngeal carcinoma. Annals of the Royal College of Surgeons of England 2019, 101(5):325-327.

4. Chan KCA, Woo JKS, King A, et al. Analysis of Plasma Epstein-Barr Virus DNA to screen for nasopharyngeal cancer. New England Journal of Medicine 2017, 377:513-522

5. Becker M, Varoquaux AD, Combescure C, et al. Local recurrence of squamous cell carcinoma of the head and neck after radio(chemo)therapy: Diagnostic performance of FDG-PET/MRI with diffusion-weighted sequences. European Radiology 2017, 28(2):651-663

6. Chan JYW, Wong STS, Wei WI. Surgical salvage of recurrent T3 nasopharyngeal carcinoma: Prognostic significance of clivus, maxillary, temporal and sphenoid bone invasion. Oral Oncol. 2019, Epub 2019.

7. Liu J, Yu H, Sun X, Wang D et al. Salvage endoscopic nasopharyngectomy for local recurrent or residual nasopharyngeal carcinoma: a 10-year experience. Int J Clin Oncol. 2017, 22(5):834-842.

8. A preliminary report on the role of endoscopic endonasal nasopharyngectomy in recurrent rT3 and rT4 nasopharyngeal carcinoma. Wong EHC, Liew YT, Abu Bakar M et al. Eur Arch Otorhinolaryngol. 2017, 274(1):275-281.

9. Peng H, Wang SJ, Yang X et al. Modified radical neck dissection for residual neck disease after radiotherapy of nasopharyngeal carcinoma. Auris Nasus Larynx 2014, 41(5):485-90

### A22 Lung Cancer Staging

#### TC McLoud, MD

##### Massachusetts General Hospital, Harvard Medical School, Boston, Massachusetts, USA

Lung cancer remains the leading cause of cancer related mortality word-wide. Effective treatment strategies require accurate staging. The current 8^th^ edition of the TNM staging system became active in 2017 and was developed from a detailed analysis of a new large international database of lung cancer cases [1-3]. TNM-8 changes include modifications to the T classifications on the basis of 1) 1-cm increments in tumour size; 2) grouping of lung cancers that result in partial or complete lung atelectasis; 3) grouping of tumours with involvement of a main bronchus irrespective of distance from carina; 4) reassignment of diaphragmatic invasion in a terms of T classification; 5) elimination of mediastinal pleural invasion from the T classification and 6) subdivision of the M classification into different descriptors on the basis of number and site of extrathoracic metastases.

Established stage groups have been modified and others have been created. Additional issues have been addressed which include multiple primary lung cancers, lung cancers with separate tumour nodules, multiple ground-glass/lepitic lesions and consolidation as well as recommendations for lesion measurement.

The numerous changes to individual TNM descriptors and TNM/8 classification have resulted in important modifications to the stage classification [4]. The separation of T1 lung cancers into T1a, T1b and T1c components on the basis of 1-cm and 2-cms thresholds has resulted in the creating of three new stages-IA1, IA2 and IA3 respectively to describe these tumours in the absence of lymph node involvement and metastatic disease. In addition, a new stage group, Stage IIIC has been created to include locally advanced T3 and T4 lung cancers associated with N3 disease but without metastases. This reflects their relatively worse prognosis compared with that for stage IIIB. Changes have been made to stage IV on the basis of the location and extent of metastatic disease. For example intrathoracic metastatic disease including contralateral tumour nodules, pleural or pericardial spread and myocardial cardiac metastases remains classified as stage IVA. However a single metastasis to a single organ (M1b disease) is now considered stage IVA. Multiple distant metastases to a single organ or multiple organs (M1c disease) are now considered stage IVB.

Finally four distinct patterns of disease in cases of lung cancer characterised by multiple sites of pulmonary involvement have been defined [5,6].

**References**

1. Carter BW, Lichtenberger JP, 3rd, Benveniste MK, de Groot PM, Wu CC, Erasmus JJ, et al. Revisions to the TNM Staging of Lung Cancer: Rationale, Significance, and Clinical Application. Radiographics. 2018;38(2):374-91.

2. Goldstraw P, Chansky K, Crowley J, Rami-Porta R, Asamura H, Eberhardt WE, et al. The IASLC Lung Cancer Staging Project: Proposals for Revision of the TNM Stage Groupings in the Forthcoming (Eighth) Edition of the TNM Classification for Lung Cancer. J Thorac Oncol. 2016;11(1):39-51.

3. Detterbeck FC, Nicholson AG, Franklin WA, Marom EM, Travis WD, Girard N, et al. The IASLC Lung Cancer Staging Project: Summary of Proposals for Revisions of the Classification of Lung Cancers with Multiple Pulmonary Sites of Involvement in the Forthcoming Eighth Edition of the TNM Classification. J Thorac Oncol. 2016;11(5):639-50.

4. Detterbeck FC, Bolejack V, Arenberg DA, Crowley J, Donington JS, Franklin WA, et al. The IASLC Lung Cancer Staging Project: Background Data and Proposals for the Classification of Lung Cancer with Separate Tumour Nodules in the Forthcoming Eighth Edition of the TNM Classification for Lung Cancer. J Thorac Oncol. 2016;11(5):681-92.

5. Detterbeck FC, Marom EM, Arenberg DA, Franklin WA, Nicholson AG, Travis WD, et al. The IASLC Lung Cancer Staging Project: Background Data and Proposals for the Application of TNM Staging Rules to Lung Cancer Presenting as Multiple Nodules with Ground Glass or Lepidic Features or a Pneumonic Type of Involvement in the Forthcoming Eighth Edition of the TNM Classification. J Thorac Oncol. 2016;11(5):666-80.

6. Travis WD, Brambilla E, Noguchi M, Nicholson AG, Geisinger KR, Yatabe Y, et al. International association for the study of lung cancer/american thoracic society/european respiratory society international multidisciplinary classification of lung adenocarcinoma. J Thorac Oncol. 2011;6(2):244-85.

### A23 Rectal Cancer MRI: Pre-Operative Staging

#### KS Jhaveri^1,2^

##### ^1^University of Toronto, ON, Canada; ^2^University Health Network, Mt.Sinai & WCH, Toronto, Canada

Rectal cancer is defined as adenocarcinoma within in the distal 15 cm of the gastrointestinal tract as measured from the anal verge. Surgical resection achieving negative margins on postoperative histopathology is considered the standard optimal locally curative therapy. The initial locoregional staging is performed to determine which patients require preoperative chemoradiation therapy (CRT) for downstaging prior to surgery or to plan surgery in those not requiring CRT with the intent to obtain a negative margin post-operatively. MRI is the imaging test of choice in local staging of rectal cancer. MRI accurately assesses extent of extramural tumour spread and relationship to mesorectal fascia as well as sphincters, which are critical for treatment plan and surgical approach. Rectal cancer MRI at our institution includes multiplanar and high-resolution oblique T2-weighted, axial T1-weighted, diffusion-weighted imaging and Gadolinium enhanced T1-weighted sequences .The mandatory part of this protocol is the T2-weighted imaging; the other sequences are considered optional for baseline staging although DWI is recommended in most guidelines.

MRI staging of rectal cancer essentially evaluates the tumour location and relationship to MRF and sphincters, tumour size, extent of extramural spread (T stage), peritoneal reflection, EMVI, lymph nodes, and bony metastasis. A structured synoptic MRI report is recommended to ensure that all necessary features are included. For tumour localisation, the distance of the inferior tumour border to the anal verge is measured. The proximity of the inferior border of the tumour to the top border of the anal sphincters guides the selection of sphincter-preservation surgery for low rectal tumours. Differentiation of T1 tumours from T2 tumours on MRI is not very reliable and ERUS maybe considered if local excision is planned. For T3 tumours, the shortest distance between the most penetrating part of the tumour and the mesorectal fascia (MRF) should be measured. The MRF is not circumferential at or above the peritoneal reflection and here, it covers the posterior or posterolateral aspects of mesorectal fat of the rectum. Tumour–MRF distance of more than 1 mm is a reliable predictor for negative margins after total mesorectal excision. In the presence of satellite nodules, the shortest distance between the nodules and the MRF should also be reported. Tumour involving the visceral peritoneum is staged as T4a. EMVI status on initial MRI staging has been suggested by some studies as a prognostic factor for the stratification of patients for selecting the appropriate treatment, especially for indicating adjuvant therapy and its intensity. In the TNM system, disease involving only the regional nodes, including the mesorectal and internal iliac nodes, accounts for the N stage; involvement of other nodes is regarded as metastasis (M stage). The most commonly advocated size cutoffs for the diagnosis of positive nodes are in the range of 5–8 mm; however, adding morphologic features, such as irregular contour and mixed signal intensity, to a size cut-off can increase the diagnostic accuracy.

This abstract has been previously published.

References

1: Nougaret S, Jhaveri K, Kassam Z, Lall C, Kim DH. Rectal cancer MR staging: pearls and pitfalls at baseline examination. Abdom Radiol (NY). 2019 May 21.:[Epub ahead of print]

2: Recio-Boiles A, Hammad H, Howell K, Kalb BT, Nfonsam VN, Scott AJ, Babiker HM, Elquza E. Locally Advanced Rectal Cancer Evaluation by Magnetic Resonance Imaging after Neoadjuvant Therapy on Decision Making: Cancer Center Experience and Literature Review. J Gastrointest Cancer. 2019 May 4.[Epub ahead of print]

3: Chan BP, Patel R, Mbuagbaw L, Thabane L, Yaghoobi M. EUS versus Magnetic Resonance Imaging in Staging Rectal Adenocarcinoma: A Diagnostic Test Accuracy Meta-Analysis. Gastrointest Endosc. 2019 Apr 17. [Epub ahead of print]

4: Kennedy ED, Simunovic M, Jhaveri K, Kirsch R, Brierley J, Drolet S, Brown C, Vos PM, Xiong W, MacLean T, Kanthan S, Stotland P, Raphael S, Chow G, O'Brien CA, Cho C, Streutker C, Wong R, Schmocker S, Liberman S, Reinhold C, Kopek N, Marcus V, Bouchard A, Lavoie C, Morin S, Périgny M, Wright A, Neumann K, Clarke S, Patil NG, Arnason T, Williams L, McLeod R, Brown G, Mathieson A, Pooni A, Baxter NN. Safety and Feasibility of Using Magnetic Resonance Imaging Criteria to Identify Patients With “Good Prognosis” Rectal Cancer Eligible for Primary Surgery: The Phase 2 Nonrandomised QuickSilver Clinical Trial. JAMA Oncol. 2019 Apr 11[Epub ahead of print]

5: Ale Ali H, Kirsch R, Razaz S, Jhaveri A, Thipphavong S, Kennedy ED, Jhaveri KS. Extramural venous invasion in rectal cancer: overview of imaging, histopathology, and clinical implications. Abdom Radiol (NY). 2019 Jan;44(1):1-10. doi: 10.1007/s00261-018-1673-2. Review. PubMed PMID: 29967984.

6: Gollub MJ, Arya S, Beets-Tan RG, dePrisco G, Gonen M, Jhaveri K, Kassam Z, Kaur H, Kim D, Knezevic A, Korngold E, Lall C, Lalwani N, Blair Macdonald D, Moreno C, Nougaret S, Pickhardt P, Sheedy S, Harisinghani M. Use of magnetic resonance imaging in rectal cancer patients: Society of Abdominal Radiology (SAR) rectal cancer disease-focused panel (DFP) recommendations 2017. Abdom Radiol (NY). 2018 Nov;43(11):2893-2902.

7: Jhaveri KS, Hosseini-Nik H, Thipphavong S, Assarzadegan N, Menezes RJ, Kennedy ED, Kirsch R. MRI Detection of Extramural Venous Invasion in Rectal Cancer: Correlation With Histopathology Using Elastin Stain. AJR Am J Roentgenol. 2016 Apr;206(4):747-55. doi: 10.2214/AJR.15.15568. Epub 2016 Mar 2.

8: Tarulli E, Thipphavong S, Jhaveri K. A structured approach to reporting rectal cancer with magnetic resonance imaging. Abdom Imaging. 2015 Oct;40(8):3002-11. doi: 10.1007/s00261-015-0518-5. Review. PubMed PMID: 26239398.

9: Snelgrove RC, Subendran J, Jhaveri K, Thipphavong S, Cummings B, Brierley J, Kirsch R, Kennedy ED. Effect of Multidisciplinary Cancer Conference on Treatment Plan for Patients With Primary Rectal Cancer. Dis Colon Rectum. 2015 Jul;58(7):653-8. doi: 10.1097/DCR.0000000000000390. PubMed PMID: 26200679.

10: Jhaveri KS, Hosseini-Nik H. MRI of Rectal Cancer: An Overview and Update on Recent Advances. AJR Am J Roentgenol. 2015 Jul;205(1):W42-55. doi: 10.2214/AJR.14.14201. Review. PubMed PMID: 26102418.

11: Furey E, Jhaveri KS. Magnetic resonance imaging in rectal cancer. Magn Reson Imaging Clin N Am. 2014 May;22(2):165-90, v-vi. doi: 10.1016/j.mric.2014.01.004. Review. PubMed PMID: 24792676.

12: Beets-Tan RGH, Lambregts DMJ, Maas M, Bipat S, Barbaro B, Curvo-Semedo L, Fenlon HM, Gollub MJ, Gourtsoyianni S, Halligan S, Hoeffel C, Kim SH, Laghi A, Maier A, Rafaelsen SR, Stoker J, Taylor SA, Torkzad MR, Blomqvist L. Correction to: Magnetic resonance imaging for clinical management of rectal cancer: Updated recommendations from the 2016 European Society of Gastrointestinal and Abdominal Radiology (ESGAR) consensus meeting. Eur Radiol. 2018 Jun;28(6):2711.

13: Beets-Tan RGH, Lambregts DMJ, Maas M, Bipat S, Barbaro B, Curvo-Semedo L, Fenlon HM, Gollub MJ, Gourtsoyianni S, Halligan S, Hoeffel C, Kim SH, Laghi A, Maier A, Rafaelsen SR, Stoker J, Taylor SA, Torkzad MR, Blomqvist L. Magnetic resonance imaging for clinical management of rectal cancer: Updated recommendations from the 2016 European Society of Gastrointestinal and Abdominal Radiology (ESGAR) consensus meeting. Eur Radiol. 2018 Apr;28(4):1465-1475. doi: 10.1007/s00330-017-5026-2. Epub 2017 Oct 17.

14: Dijkhoff RAP, Beets-Tan RGH, Lambregts DMJ, Beets GL, Maas M. Value of DCE-MRI for staging and response evaluation in rectal cancer: A systematic review. Eur J Radiol. 2017 Oct;95:155-168. doi: 10.1016/j.ejrad.2017.08.009. Epub 2017 Aug 12.

15: Morino M, Risio M, Bach S, Beets-Tan R, Bujko K, Panis Y, Quirke P, Rembacken B, Rullier E, Saito Y, Young-Fadok T, Allaix ME; European Association for Endoscopic Surgery; European Society of Coloproctology. Early rectal cancer: the European Association for Endoscopic Surgery (EAES) clinical consensus conference. Surg Endosc. 2015 Apr;29(4):755-73. doi: 10.1007/s00464-015-4067-3. Epub 2015 Jan 22.

## 11:30 – 13:00 Pitfalls in Diagnostic Imaging

### A24 Pitfalls in paediatric oncologic imaging

#### Kieran McHugh

##### Great Ormond Street Hospital, London, UK

Radiologists are very good at opting to image new abdominal or superficial masses in children with ultrasound. It is easy to forget, however, to assess for regional lymphadenopathy, which is very important for many malignancies, particularly limb tumours and sarcomas (the inguinal or axillary regions for lower and upper limb tumours respectively should also be routinely evaluated). Pediatric tumours tend not to invade other organs but they can often be adherent to adjacent viscera – real time dynamic ultrasound can be very useful to assess movement of one organ relative to another. When considering cross-sectional imaging, CT is easier than MRI to do in children as CT scanning is so fast. MRI, however, is often the better test and is superior for assessing spinal canal invasion, chest wall involvement by tumour and bone marrow disease. MRI is best for pelvic, liver, paraspinal and neck masses as a general rule, and ideally should be performed for all new abdominal masses at initial presentation. Due to their usual lack of mediastinal and intra-abdominal fat, non-contrast enhanced CT is generally a waste of time and best avoided in children. Dual or triple phased enhanced CT is seldom necessary (all masses should have been assessed with Doppler ultrasound before a CT) and should also be avoided to reduce the radiation burden from CT.

This abstract has been previously published.

### A25 Paediatric PET/CT: physiologic uptake, normal variants and pitfalls

#### Pek-Lan Khong

##### The University of Hong Kong, Hong Kong SAR

The normal distribution and physiologic variants of 18F-FDG uptake differs between children and adults and it is important to recognise this to avoid pitfalls in interpretation. This is especially important when the location of uptake can obscure or mimic pathologies.

*Brown adipose tissue*

Brown adipose tissue contains large quantities of mitochondrion and induces non-shivering thermogenesis to control body temperature and energy expenditure. Its presence is related to BMI, age, sex and outdoor temperature, with age being the most important factor in our cohort (younger patients have a higher prevalence) [1]. It is mostly detected by symmetrical uptake on PET in the supraclavicular region and lower neck. Other sites include the axillae, mediastinum, perivascular, paravertebral, intercostal and infra-diaphragmatic regions.

*Thymus Gland*

The normal thymus gland in children typically has diffuse, very low grade uptake, which generally disappears during adolescence. The normal thymus gland may shrink during stress (e.g. chemotherapy), and upon recovery become larger, indicating thymic rebound hyperplasia. The thymus gland is diffusely enlarged with a smooth convex contour and homogenous low grade uptake. Although this commonly occurs 2-6 months after chemotherapy, thymic rebound can develop over a period as short as one week, and may persist for 12-24 months [2]. It has been found that the time course of FDG uptake reaches a peak around 10 months after therapy, and will decline slowly thereafter [3]. Occasionally, thymic hyperplasia may extend superiorly and appear as a superior mediastinal nodule which may be confused as adenopathy [4].

*Adenoids*

Adenoids are prominent in children compared to adults and the uptake may be moderate in the lymphatic tissue peaking at 6-8 years of age [5]. Its symmetrical shape and diffuse uptake usually helps to distinguish it from pathology.

*Cervical and mesenteric lymph nodes*

Cervical lymph nodes larger than 10mm in short axis diameter are common in children, of which some maybe reactive lymph nodes and may show increased uptake. Although uptake is generally higher in malignant compared to benign lymph nodes, there is no well accepted cut-off value and overlap exists [6]. CT features including shape, configuration and enhancement are important in improving specificity.

Mesenteric lymph nodes are commonly prominent in children, especially in the right lower quadrant, and are non-specific. Lymph node size peaks at around 10 years of age then decreases with age, and asymptomatic children may have mesenteric nodes measuring up to 10mm in short axis diameter [7].

*Physeal plate*

Skeletally immature patients demonstrate physiological linear uptake along the physes and apophyses.

*Skeletal muscle*

Babies suckling on pacifiers during uptake time may have skeletal muscle uptake in the masseter muscles and tongue, and crying may cause uptake in the diaphragmatic crus and intercostal muscles.

*Ovaries and endometrium*

Physiologic uptake is seen in the endometrium during midcycle and during menstruation, whilst normal ovarian uptake is seen at mid-cycle ovulation and in corpus luteal cysts.

References

1) Leung TM, Lam KSL, Wong CY, Khong PL. Prevalence and factors associated with brown adipose tissue detected by 18F-fluorodeoxyglucose positron emission tomography/computed tomography in Southern Chinese. Journal of the Hong Kong College of Radiologists 2013; 16:183-190.

2) Nasseri F, Eftekhari F. Clinical and Radiologic normal and abnormal thymus: pearls and pitfalls. Radiographics 2010; 30: 413-428

3) Goethals I, Hoste P, Vriendt CD et al, Time-dependent changes in ^18^F-FDG activity in the thymus and bone marrow following combination chemotherapy in paediatric patients with lymphoma. Eur J Nucl Med Mol Imaging 2010;37:462-467.

4) Smith C, Schoder H, Yeung HWD. Thymic extension in the superior mediastinum in patients with thymic hyperplasia: potential cause of false-positive findings on 18F-FDG PET/CT. AJR 2007; 188:1716-21.

5) Shammas A, Lim R, Charron M. Pediatric FDG PET/CT: physiologic uptake, normal variants and benign conditions. Radiographics 2009; 29: 1467-68.

6) Vali R, Bakari AA, Marie E et al, FDG uptake in cervical lymph nodes in children without head and neck cancer. Pediatr Radiol 2017; 47: 860-67.

7) Simanovsky N, Hiller N. Importance of sonographic detection of enlarged abdominal lymph nodes in children. J Ultrasound Med 2007; 26: 581-4.

### A26 Pitfalls in Genitourinary Imaging

#### Vargas HA

##### Memorial Sloan Kettering Cancer Center, New York, NY, USA

The best chance of making a positive impact on patient management and outcomes through imaging is to ensure optimal image acquisition, interpretation and communication with all parties involved in patient care. In genitourinary imaging pitfalls arise in these three domains, and this lecture will focus on systematic approaches to recognise and avoid them as well as tips to prevent them. There will be a focus on cancer and cancer mimics, and a variety of cancers involving the urinary system and male and female genital organs will be covered.

## Afternoon Session: 14:15 – 15:30 Gynaecological Cancer

### A27 Ovarian Masses: Differential Diagnosis and Clinical Implications

#### Gabriele Masselli, MD Phd (gabriele.masselli@uniroma1.it)

##### Policlinico Umberto I. Sapienza University Rome Italy

Adnexal masses, both incidental and symptomatic, are a common finding in clinical practice and pose a challenging diagnostic problem. Approximately 25% of sonographically identified adnexal masses have indeterminate origin. Despite these lesions have low rate of malignancy (10-20%), patients are frequently referred for surgery.

A multidisciplinary approach with physical examination, imaging exams and laboratory tests is necessary for the evaluation of an adnexal mass.

Primary ovarian tumours can be classified, according to tumour origin, in epithelial, germ cell and sex cord-stromal tumours. Ovaries are also affected by metastastic tumours. [1]

Epithelial tumours account for approximately 85 % of ovarian malignancy: the most common type is serous carcinoma. Dermoid cyst (mature cystic teratoma) is the most common benign ovarian neoplasm. [2]

Ovarian neoplasms may be benign, borderline or malignant and may appear on imaging as unilocular cyst, multilocular cyst, mixed cystic and solid, predominantly solid.

Although ovarian tumours have similar clinical and radiologic findings, each type may present predominant or specific key features. Even if there are many overlapping morphologic characteristics and corresponding imaging features, a thick, irregular wall, thick septa, papillary projections and a large soft-tissue component with necrosis are malignant features. [3]

Ultrasound (US) (performed with sovrapubic and/or transvaginal) is the first-line imaging investigation for the suspected adnexal mass for its low invasivity, high availability and low cost. It allows to investigate morphological features (such as cystic or solid components) and vascular characteristics on doppler exam.

Computed Tomography (CT) plays an important role in the staging of the disease (especially for metastatic disease) and in the evaluation after therapy, while it has a poor role in primary detection and characterisation of adnexal mass. It can be useful in masses wich contain calcifications and fat, such as teratomas.

Imaging of the indeterminate adnexal masses is one of the most common gynecologic indication for MRI and is often used in complex adnexal mass with malignant features, in pelvic mass of equivocal origin and in solid adnexal mass [4].

A MRI protocol to investigate adnexal mass comprises T1- and T2-weighted sequences to evaluate morphological features, T1 fat sat -weighted images to detect haemorrhagic or fatty areas and contrast-enhanced T1 sequences to evaluate solid components, enhancing septa and metastatic implants.

MRI is the most accurate modality in adnexal mass characterisation, and many of the benign adnexal lesions considered indeterminate at CT and US may be confidently diagnosed as benign with MRI [5]

References

1. World Health Organisation Classification of Tumours (2003) Pathology and genetics of tumours of the breast and female genital organs. IARC

2. Foti PV, Attinà G, Spadola S et al. MR imaging of ovarian masses: classification and differential diagnosis. Insights Imaging (2016) 7:21–41

3. Jung SE, Lee JM, Rha SE et al. CT and MR imaging of ovarian tumours with emphasis on differential diagnosis**.** Radiographics. 2002 Nov-Dec;22(6):1305-25

4. Chilla B, Hauser N, Singer G et al (2011) Indeterminate adnexal masses at ultrasound: effect of MRI imaging findings on diagnostic thinking and therapeutic decisions. Eur Radiol 21(6):1301–10

5. Masch WR, Daye D, Lee SI. MR Imaging for Incidental Adnexal Mass Characterisation. Magn Reson Imaging Clin N Am. 2017 Aug;25(3):521-543

### A28 Ovarian Cancer: Post treatment surveillance

#### A Sahdev

##### St Bartholomew’s Hospital, Barts Health, London, UK

Ovarian cancer is the 6^th^ most common cancer and 7^th^ commonest cause of death in women worldwide. The surveillance of ovarian cancer patients after initial treatment is a challenging question in clinical practice. Several strategies have been employed following completion of primary treatment. Most treated women undergo long-term follow-up. Clinical examination, serum CA 125, physical examination, and imaging examinations have been employed with different schedules for follow-up . Although there are no consensus guidelines for surveillance, most recommend a pelvic examination every 2 to 4 months for the first 4 years after treatment and every 6 months for the next 3 years [1]. Imaging tests including x-rays, CT scans, MRI scans, ultrasound studies are used for investigating symptoms and rising CA125 levels [2,3]. It has been suggested routine imaging is not be effective in improving survival or quality of life and may not be cost-effective [4]. Varying surveillance strategies based on age, tumour subtype and stage, presenting and nadir CA125 levels have been proposed to direct surveillance strategies [5]. The application of a single surveillance strategy for all ovarian cancer has shown little benefit. However, by stratifying the likelihood of risk for recurrence, beneficial protocols are likely to arise. Aggressive and high stage tumours, with high risk or relapse are likely to benefit from routine imaging for allow detection and appropriate management of early relapse. With increasing treatment options, this strategy may improve survival. Patients at low risk of recurrence are unlikely to benefit from routine imaging surveillance. The choice of imaging modality and frequency of imaging also has no consensus. CT remains the most widely applied modality for both asymptomatic and symptomatic patients. Transvaginal US in low risk patients, for detection of local early pelvic recurrence is often applied. In patients with a rising CA125 and undetected disease on CT, MRI with diffusion weighted imaging and ^18^F FDG PET CT has been advocated [6].

This abstract has been previously published.

References

1. Rustin GJS What Surveillance Plan Should Be Advised for Patients in Remission After Completion of First-Line Therapy for Advanced Ovarian Cancer? International Journal of Gynecologic Cancer 2010;20:S27-S28.

2. Esselen KM, Cronin AM, Bixel K, et al. Use of CA-125 Tests and Computed Tomographic Scans for Surveillance in Ovarian Cancer. JAMA Oncol. 2016;2(11):1427–1433. doi:10.1001/jamaoncol.2016.1842

3. Low RN, Saleh F, Song SY, Shiftan TA, Barone RM, Lacey CG, Goldfarb PM. Treated ovarian cancer: comparison of MR imaging with serum CA-125 level and physical examination--a longitudinal study. Radiology. 1999 May;211(2):519-28.

4. Ledermann JA, Raja FA, Fotopoulou C, Gonzalez-Martin A, Colombo N, Sessa C; ESMO Guidelines Working Group. Newly diagnosed and relapsed epithelial ovarian carcinoma: ESMO Clinical Practice Guidelines for diagnosis, treatment and follow-up. Ann Oncol. 2018 Oct 1;29(Supplement_4)

5. Rizzuto I, Stavraka C, Chatterjee J, et al Risk of Ovarian Cancer Relapse Score: A Prognostic Algorithm to Predict Relapse Following Treatment for Advanced Ovarian Cancer International Journal of Gynecologic Cancer 2015;25:416-422.

6. Gu P, Pan LL, Wu SQ, Sun L, Huang G. CA 125, PET alone, PET-CT, CT and MRI in diagnosing recurrent ovarian carcinoma: a systematic review and meta-analysis. Eur J Radiol. 2009 Jul; 71(1):164-74. Epub 2008 Apr 18

## 16:00 – 17:30 Paediatric Oncology

### A29 Paediatric bone tumours

#### Anne MJB Smets (a.m.smets@amsterdamumc.nl)

##### Department of Radiology, Amsterdam UMC – location AMC, University of Amsterdam, The Netherlands

Imaging characteristics of a bone lesion- a malignant tumour, a benign tumour or a tumour-like process- can be very specific, but may also be misleading at times, making characterisation and differentiation more challenging or even impossible [1, 2]. Malignant bone tumours are rare in the paediatric age group. Ewing sarcoma and osteosarcoma are the most frequent primary malignant bone tumours with a variable incidence according to age. Other malignant bone tumours such as primary skeletal lymphoma, chondrosarcoma, fibrosarcoma, haemangioendothelioma and adamantinoma are even rarer [3]. In young children bone metastases may be the presenting manifestation of neuroblastoma and leukaemia. Bone lesions in a context of Langerhans cell histiocytosis (LCH), now considered a neoplasm, occur mainly in the flat bones, spine and proximal long bones.

The most common benign condition that can show aggressive features and may thus mimic a malignant bone tumour on imaging studies is osteomyelitis, particularly when the patient has no fever [2, 4].

Plain radiograph is the initial and most useful examination for differentiating benign from malignant bone processes. CT and MRI are in many cases the next diagnostic test.

Paediatric bone has specific features and particularities such as growth, bone marrow conversion and variation of vascularisation of the bone with age. Furthermore, certain bone lesions are age-related [5], hence age is an important element in the differential diagnosis. A thorough analysis of the images, including the number of lesions, the location, the appearance and size of the lesion(s), and the appearance of the adjacent bone and periosteal reaction, is fundamental.

In case of multiple lesions, LCH, chronic recurrent multifocal osteomyelitis and polyostotic fibrous dysplasia are possible diagnoses.

The type of bone, flat, short or long, in which the lesion is located, as well as epi-, meta- or diaphysis is important in the differential diagnosis.

The size of the lesion is not a very specific feature although lesions larger than 5-6 cm are more suspicious for malignancy. The morphology of the lesion is equally non-specific: most benign lesions are elliptical but some malignant tumours can have the same appearance (lymphoma, low-grade osteosarcoma).

Benign lesions usually grow at a slow pace and have sharp borders. Malignant lesions commonly have poorly defined margins and show cortical destruction.

A periosteal apposition occurs whenever an infection or a tumour, either malignant or benign, irritates the periosteum or as a reaction to trauma.

The pattern of periosteal reaction can be benign (as seen in benign lesions or trauma) or aggressive (as seen in malignancies, infections or LCH).

Benign bone tumours generally have well-defined and often sclerotic margins, show cortical expansion and may produce solid periosteal reaction. Malignant tumours usually have poorly defined margins, show cortical destruction and periosteal reaction of the spiculated, onionskin or interrupted type and are most of the time accompanied by a soft tissue mass. In conclusion, a meticulous analysis of all available imaging studies with the age of the patient in mind is required for a reliable diagnosis or differential diagnosis.

References

1. Kan JH. Major pitfalls in musculoskeletal imaging-MRI. Pediatr Radiol 2008; 38 Suppl 2:S251-255.

2. McCarville MB. The child with bone pain: malignancies and mimickers. Cancer imaging 2009; 9 Spec No A:S115-121.

3. Wootton-Gorges SL. MR imaging of primary bone tumours and tumour-like conditions in children. Magn Reson Imaging Clin N Am 2009; 17:469-487.

4. Khung S, Budzik JF, Amzallag-Bellenger E, et al. Skeletal involvement in Langerhans cell histiocytosis. Insights Imaging 2013; 4:569-579.

5. Chan BY, Gill KG, Rebsamen SL, et al. MR Imaging of Pediatric Bone Marrow. Radiographics 2016; 36(6):1911-1930.

### A30 Pediatric Chest Masses

#### Stephan D. Voss, MD, PhD

##### Department of Radiology, Boston Children’s Hospital, Harvard Medical School, Boston, MA USA

Pediatric chest masses include both mediastinal tumours and pulmonary parenchymal neoplasms. Primary pulmonary tumours are rare in childhood, with metastatic disease a far more common cause of pulmonary malignancy. Of the primary pediatric neoplasms commonly encountered, approximately 3/4 are malignant with the remainder representing benign tumours. Although pediatric pulmonary tumours are rare, they can contribute to considerable morbidity and mortality secondary to their location and mass effect on adjacent structures such as the heart and airways, as well as local tissue and vascular invasion. Some tumours, such as NUT midline carcinoma, have particularly aggressive features and a propensity for metastatic spread. In contrast to adult lung cancers, which are commonly epithelial tumours, most primary pediatric lung tumours are either endobronchial or mesenchymal in origin.

The purpose of this presentation is to provide an overview of the common benign and malignant pediatric pulmonary neoplasms. We will discuss ages at presentation, distinctive imaging features characteristic of specific pulmonary neoplasms. The choice of diagnostic imaging techniques, including both in atomic and functional imaging, and the role of imaging in diagnosis, pre-operative staging, assessing response to therapy, and in long-term follow-up will be reviewed.

**References**

1. Guillerman RP, Vogelius E, Pinto-Rojas A, and Parham DM. (2015) Malignancies of the pediatric lower respiratory tract. In: Parham DM et al. (eds) *Pediatric Malignancies: Pathology and Imaging, 227-243*. Springer New York

2. Lyons K, Guillerman RP, and McHugh K. (2014) Pulmonary and extrathymic mediastinal tumours. In: Garcia-Pena P. and Guillerman RP (eds). *Pediatric Chest Imaging, 349-372.* Springer New York

3. Weldon CB, Shamberger R. (2008) Pediatric pulmonary tumours: primary and metastatic. *Semin Pediatr Surg.* 17(1):17-29.

### A31 Neuroblastoma and Image Defined Risk Factors (IDRF’s)

#### Stephan D. Voss, MD, PhD

##### Department of Radiology, Boston Children's Hospital, Harvard Medical School, Boston, MA USA

Neuroblastoma is the most common non-CNS pediatric solid tumour, making up approximately 7% of all pediatric neoplasms. The International Neuroblastoma Staging System (INSS), which was established in the late 1980’s, has been universally accepted and used for several decades in the staging of neuroblastoma. The INSS staging system relies primarily on characterisation of the primary tumour, identification of locoregional lymph node involvement, invasion of adjacent structures, and presence or absence of metastatic disease, and was based both on initial imaging and extent of tumour remaining after surgical resection. In addition to the identification of other factors (histology, age, N-myc) important for establishing overall prognosis, it became clear that the INSS – because it relied on surgical criteria established at the time of tumour resection – was of limited use in risk stratifying patients prior to initiating therapy.

As a result the International Neuroblastoma Risk Group (INRG) recently developed a revised staging system for neuroblastoma The INRG staging system focuses on establishing tumour stage prior to surgery or chemotherapy and is based on the presence of one or more of 20 agreed upon “image defined risk factors” (IDRF’s), which allow patients to be assigned to specific risk groups at the time of diagnosis prior to initiating treatment. These IDRF’s are surgical risk factors, based on imaging, that could influence the surgical resectability of a tumour and the likelihood of achieving a gross total resection.

The purpose of this presentation is to provide an overview and practical approach to using IDRF’s in the initial staging evaluation of newly diagnosed neuroblastoma patients. We will discuss the role of both anatomic and functional imaging techniques both in the initial comprehensive staging evaluation, and in assessing response to therapy.

**References**

1. Chen AM, Trout AT, and Towbin AJ. (2018) A review of neuroblastoma image-defined risk factors on magnetic resonance imaging. *Pediatric Radiology;* 48(9):1337-1347

2. Brisse HJ, McCarville MB, Granata C, Krug KB, Wootton-Gorges SL, Kanegawa K, Giammarile F, Schmidt M, Shulkin BL, Matthay KK, Lewington VJ, Sarnacki S, Hero B, Kaneko M, London WB, Pearson AD, Cohn SL, Monclair T; International Neuroblastoma Risk Group Project. (2011) Guidelines for imaging and staging of neuroblastic tumours: consensus report from the International Neuroblastoma Risk Group Project. *Radiology;* 261(1): 243-57.

3. Dumba M, Jawad N, McHugh K. (2015) Neuroblastoma and nephroblastoma: a radiological review. *Cancer Imaging.* Apr 8; 15:5

### A32 Malignant renal tumours in children

#### Beth McCarville, MD

##### St. Jude Children’s Research, Memphis, TN, USA

Renal tumours account for about 6% of all pediatric cancers with Wilms tumour comprising approximately 95% of cases. Wilms tumour is also the second most common intraabdominal malignancy in children. Other rare pediatric renal malignancies include clear cell sarcoma, rhabdoid tumour, translocation associated renal cell carcinoma, papillary renal cell carcinoma, renal medullary carcinoma and primitive neuroectodermal tumour [1,2]. These tumours have unique clinical and imaging features that are helpful in narrowing the differential diagnosis. When a child presents with a possible abdominal mass, imaging is needed to confirm its presence and determine the organ of origin. Subsequently, the radiologist is one of the first physicians to be involved in the care of these patients. Therefore, it is crucial for radiologists who interpret the imaging of children to have an awareness of the unique clinical and imaging features of renal malignancies to properly direct further management. Due to its availability, portability, lack of radiation or need for sedation, ultrasound is typically the first line imaging modality used to evaluate a child with a suspected abdominal mass. Because of the complex nature of renal tumours and the propensity to spread to lymph nodes, solid organs and the lungs, additional cross-sectional imaging with CT and/or MRI is warranted. In this presentation I will review the salient clinical and imaging features of the most common malignant pediatric renal tumours. Representative clinical cases will be illustrated with ultrasound, CT and MRI. At the end of my lecture, attendees should be able to identify the most likely diagnosis of these renal tumours based on their imaging appearance, patient demographics and clinical presentation.

References

1. Shehata BM, Naguib MM, Lin J, Khanna G. In Pediatric Malignancies:Pathology and Imaging. 1^st^ Ed. Parham, Khoury and McCarville Editors. 2015 Springer; 271-295.

2. Lowe LH et al.. Pediatric renal masses: Wilms tumour and beyond. Radiographics. 2000;20(6):1585-603

## Wednesday 9th October – Morning Session: 08:30 – 10:30 Prostate Cancer

### A33 Delivering PI-RADS pathway benefits

#### Anwar Padhani

##### Paul Strickland Scanner Centre, Mount Vernon Cancer Centre, UK

High-quality evidence shows that MRI in biopsy-naive men can reduce the number of men who need prostate biopsy and can reduce the number of diagnoses of clinically insignificant cancers that are unlikely to cause harm. In men with prior negative biopsy results who remain under persistent suspicion, MRI improves the detection and localisation of life-threatening prostate cancer with greater clinical utility than the current standard of care, systematic transrectal US-guided biopsy. Systematic analyses show that MRI-directed biopsy increases the effectiveness of the prostate cancer diagnosis pathway. The incorporation of MRI-directed pathways into clinical care guidelines in prostate cancer detection has begun. The PI-RADS MRI-directed biopsy pathway enables the delivery of key diagnostic benefits to men suspected of having cancer based on clinical suspicion. This lecture demonstrates how the MRI pathway should be incorporated into routine clinical practice using case examples, and relays the challenges of delivering the positive health impacts needed by men suspected of having clinically significant prostate cancer.

### A34 Biochemical Recurrence in Prostate Cancer: What is the Role of Imaging?

#### R. J. Hicks

##### Cancer Imaging, Peter MacCallum Cancer Centre, Melbourne, Australia

Bone-scanning techniques and contrast-enhanced abdomino-pelvic CT (ceCT) are still widely used in the restaging of prostate cancer at biochemical recurrence (BCR) following radical prostatectomy or definitive radiotherapy with curative intent. However, both modalities lack sufficient sensitivity in the context of the PSA levels that are generally considered to be suitable for salvage therapy with surgery or template pelvic radiotherapy. Accordingly, they are only recommended within most specialist guidelines when PSA levels are relatively high, typically >10-20 ng/ml (1). While Tc-99m scintigraphy, especially when performed as a combined SPECT/CT examination, remains reasonably sensitive for the detection of bone metastasis at these levels of PSA elevation, its diagnostic performance is inferior to that of F-18 fluoride PET/CT bone scanning, which has advantages in sensitivity, specificity and prognostic stratification (2). In selecting patients for treatment with bone-seeking therapeutic radiopharmaceuticals, such as Ra-223 or Sm-153 EDTMP, or for planning possible palliative irradiation of painful bone metastases, these investigations remain useful for demonstrating active osteoblastic metastases. Assessment of nodal disease in the abdomen and pelvis using ceCT suffers from low sensitivity and imperfect specificity (3) and is increasingly discouraged by uro-oncology experts (4). In the context of low PSA levels, multi-parametric magnetic resonance imaging (mpMRI) of the pelvis and novel molecular imaging techniques are entering into clinical practice in the hope of identifying patients with salvageable loco-regional recurrences or oligometastatic disease amenable to aggressive treatment, or, alternatively, identifying patients needing systemic therapy including androgen deprivation therapy (ADT) in the first instance . These newer modalities provide improved sensitivity and specificity for the detection and characterisation of sites of residual malignant involvement in the prostate bed, regional nodes and more distant metastatic sites. Whole-body MRI has also been advocated for the latter purpose but will not be further discussed here. Depending on the nature of prior treatment, mpMRI is probably the most sensitive technique for prostate bed recurrence but is particularly compromised in the context of brachytherapy seeds and appears to be less sensitive for detection of nodal disease in the pelvis than molecular imaging techniques (5). PET/CT using choline analogues, such as C-11 choline and F-18 fluorocholine (FCH), the synthetic amino acid F-18-fluciclovine (Axumin) and various prostate-specific membrane antigen (PSMA) ligands have all be shown to be diagnostically superior to conventional restaging techniques with PSMA PET/CT having the highest sensitivity and specificity for disease detection, especially at very low levels of PSA elevation (6). The most widely evaluated of these agents is Ga-68 PSMA-11. The high accuracy of this agent has also been demonstrated to significantly impact management of patients with BCR (7). Practical advantages of newer F-18-based PSMA agents may see them replace Ga-68 PSMA-11 in some situations (8, 9). The ability to assess PSMA-expression also provides the opportunity to select patients with disseminated disease for radionuclide therapy, although this has been primarily evaluated and shown to be effective for patients with metastatic castrate-resistant prostate cancer (10) rather than in the context of BCR While false negative results can occur with small disease deposits (11), some aggressive neuroendocrine-differentiated prostate cancers can also lack PSMA expression. These tend to be better visualised using F-18 fluoro-deoxyglucose (FDG) PET/CT, which has also been shown to provide prognostic stratification despite relatively poor sensitivity for most prostate cancer metastases (12). There remain many clinical questions regarding how to integrate these new and more sensitive imaging technologies into management planning, particularly whether it is appropriate to escalate the aggressiveness of treatment in the case of detection of small volume disease or to observe rather than treat empirically patients with BCR but negative scanning. Nevertheless, advanced imaging techniques is BCR are changing the therapeutic landscape of BCR by defining disease burden and guiding loco-regional salvage.

**References**

1. Gillessen S, Attard G, Beer TM, Beltran H, Bossi A, Bristow R, et al. Management of Patients with Advanced Prostate Cancer: The Report of the Advanced Prostate Cancer Consensus Conference APCCC 2017. Eur Urol. 2018;73(2):178-211.

2. Even-Sapir E, Metser U, Mishani E, Lievshitz G, Lerman H, Leibovitch I. The detection of bone metastases in patients with high-risk prostate cancer: 99mTc-MDP Planar bone scintigraphy, single- and multi-field-of-view SPECT, 18F-fluoride PET, and 18F-fluoride PET/CT. J Nucl Med. 2006;47(2):287-97.

3. Hovels AM, Heesakkers RA, Adang EM, Jager GJ, Strum S, Hoogeveen YL, et al. The diagnostic accuracy of CT and MRI in the staging of pelvic lymph nodes in patients with prostate cancer: a meta-analysis. Clin Radiol. 2008;63(4):387-95.

4. Fanti S, Minozzi S, Antoch G, Banks I, Briganti A, Carrio I, et al. Consensus on molecular imaging and theranostics in prostate cancer. Lancet Oncol. 2018;19(12):e696-e708.

5. Metser U, Chua SS, Ho B, Punwani S, Johnston E, Pouliot F, et al. The contribution of multiparametric pelvic & whole body MR to interpretation of. J Nucl Med. 2019.

6. Perera M, Papa N, Christidis D, Wetherell D, Hofman MS, Murphy DG, et al. Sensitivity, Specificity, and Predictors of Positive 68Ga-Prostate-specific Membrane Antigen Positron Emission Tomography in Advanced Prostate Cancer: A Systematic Review and Meta-analysis. Eur Urol. 2016;70(6):926-37.

7. Roach PJ, Francis R, Emmett L, Hsiao E, Kneebone A, Hruby G, et al. The Impact of 68 Ga-PSMA PET/CT on Management Intent in Prostate Cancer: Results of an Australian Prospective Multicenter Study. J Nucl Med. 2018;59(1):82-8.

8. Rowe SP, Macura KJ, Ciarallo A, Mena E, Blackford A, Nadal R, et al. Comparison of Prostate-Specific Membrane Antigen-Based 18F-DCFBC PET/CT to Conventional Imaging Modalities for Detection of Hormone-Naïve and Castration-Resistant Metastatic Prostate Cancer. J Nucl Med. 2016;57(1):46-53.

9. Ferreira G, Iravani A, Hofman MS, Hicks RJ. Intra-individual comparison of (68)Ga-PSMA-11 and (18)F-DCFPyL normal-organ biodistribution. Cancer Imaging. 2019;19(1):23.

10. Hofman MS, Violet J, Hicks RJ, Ferdinandus J, Thang SP, Akhurst T, et al. [(177)Lu]-PSMA-617 radionuclide treatment in patients with metastatic castration-resistant prostate cancer (LuPSMA trial): a single-centre, single-arm, phase 2 study. Lancet Oncol. 2018;19(6):825-33.

11. Jilg CA, Drendel V, Rischke HC, Beck T, Vach W, Schaal K, et al. Diagnostic Accuracy of Ga-68-HBED-CC-PSMA-Ligand-PET/CT before Salvage Lymph Node Dissection for Recurrent Prostate Cancer. Theranostics. 2017;7(6):1770-80.

12. Jadvar H, Desai B, Ji L, Conti PS, Dorff TB, Groshen SG, et al. Baseline 18F-FDG PET/CT parameters as imaging biomarkers of overall survival in castrate-resistant metastatic prostate cancer. J Nucl Med. 2013;54(8):1195-201.

### A35 Complication of Prostate Cancer Treatment

#### Aslam Sohaib

##### Department of Imaging, Royal Marsden Hospital, Fulham Road, London, UK

Patients with prostate cancer confined to the prostate gland have many management options. These include watchful waiting, active surveillance or active treatment with radiotherapy, hormonal therapy, minimally invasive therapy and radical prostatectomy. The decision as to which of these is selected depends on many factor including potential complications from treatment. Imaging plays a central role following treatment, most often looking for response or in the detection of recurrent disease and less commonly for treatment related complications. However it is important to assess these complications on imaging to guide further management.

Radical prostatectomy can be performed by laparoscopic or robot-assisted or as an open procedure and complications related to radical prostatectomy include urinary leaks or fistula formation. Lymphocele formation are common following pelvic side wall nodal dissection.

Radiotherapy can be delivered in a variety of ways. Conventional external beam radiotherapy has largely been replaced with more advanced (conformal) techniques eg IMRT. These allows for more accurate targeting of radiation whilst reducing toxicity to surrounding structures. The morbidity from radiation therapy relates to the volume of tissue irradiated, the dose delivered and the inherent radio-sensitivity of the organ. The distal colon and rectum are most commonly affected by pelvic radiotherapy as they lie within the radiation field. Patients with a history of previous radiation or abdominal surgery are at an increased risk as a result of adhesions as the bowel is less able to move from the radiation fields. Segments of diverticular disease may be susceptible to develop diverticulitis and fistulation. Acute toxicity usually manifests as diarrhoea, tenesmus, mucoid discharge and rectal bleeding if there is ulceration. Small bowel involvement from pelvic radiotherapy leads to abdominal pain, nausea and watery diarrhoea. Late symptoms are more insidious and can develop months to years after therapy and may be unrelated to history of symptoms of acute toxicity. If the terminal ileum is affected then malabsorption may occur. Radiation-induced injury to the urinary tract or the genital system has been reported to cause symptoms affecting quality of life in up to 30 % of prostate cancer patients. The bladder wall thickening observed at CT and MR imaging is often worse than the clinical symptoms.

Low-dose brachytherapy involves permanent placement of small radioactive seeds into the prostate under ultrasound guidance. Brachytherapy is usually contraindicated in patients with large prostates, history of transurethral resection of the prostate, moderate to severe storage symptoms and in those with a history of abdomino-perineal resection. Brachytherapy, early side effects include urinary retention, haematuria, urethritis, infection and misplaced seeds. Later effects include worsening of storage symptoms, rectal symptoms such as proctitis or faecal urgency, fistulae, radiation osteitis and urethral strictures.

Hormonal therapies are commonly used in prostate cancer either alone or in combination with radiotherapy treatment. The adverse effects of androgen deprivation therapy (ADT) include osteoporosis, greater incidence of clinical fractures, obesity, insulin resistance and greater risk for diabetes and cardiovascular disease.

Following radiotherapy, brachytherapy, or hormonal therapies usually results in glandular involution and loss of zonal differentiation on T2 weighted image and this also decreases the T2-weighted contrast between the tumour and the normal glandular tissues. Functional imaging techniques (e.g. DCE- MRI and DWI) are increasingly used to assess the prostate in the post- therapy setting

Though complications from modern treatment from prostate cancer are infrequent, imaging is key in the multidisciplinary management of these patients.

This abstract has been previously published.

**Reference**

1. Shah A., Sohaib S.A., Koh DM. (2015) Imaging of Complications and Toxicity Following Tumour Therapy: Pelvis and Genitourinary (Male). In: Kauczor HU., Bäuerle T. (eds) Imaging of Complications and Toxicity following Tumour Therapy. Medical Radiology. Springer, Cham

## 11:00 – 11:30 Keynote Lecture 3

### K2 Theranostics

#### R. J. Hicks

##### Cancer Imaging, Peter MacCallum Cancer Centre, Melbourne, Australia

Even before the advent of molecular imaging, the therapeutic potential of radionuclides was recognised. This included the topical use of Ra-226 for the treatment of skin malignancies (1) followed by the application of P-32 for haematological conditions (2) and I-131 for thyroid cancer (3). The rectilinear scanner created the basis for the selection and monitoring of treatment with radionuclides by the pairing of diagnostic scans evaluating the same cellular target as that leveraged for therapeutic effect. This capability was further expanded by whole body scanning on the gamma camera and then PET. This paradigm is now called “theranostics” and can be summarised by the statement; “If you can see it, you can treat it”. While the use of the iodine isotopes, I-123 and I-124 to select and monitor the use of I-131 in the treatment of metastatic thyroid cancer has been used in the largest number of patients and for the longest period of time, demonstrating the effectiveness of the theranostic paradigm, recent decades have seen an enlarging array of diagnostic and therapeutic pairs emerge into clinical practice. Imaging and treatment of metastatic phaeochromocytoma/paraganglioma (PCC/PGL) (4) and of neuroblastoma (5) with radio-iodinated meta-iodo-benzyl-guanidine (MIBG) represented an evolution of the concepts established by radio-iodine treatment of thyroid cancer, leveraging specific transport of a precursor for a biomolecule produced by the cancer in question; catecholamines and thyroxine, respectively. The next conceptual advance occurred with the recognition of cell surface receptors on cancer cell membranes. Leveraging the near universal and high expression of somatostatin receptors (SSRs) on low-grade neuroendocrine tumours (NETs), various radiolabeled somatostatin analogues (SSAs) have been developed for both diagnostic and therapeutic application. In-111 DTPA-pentetreotide (Octreoscan) was the first of these (6) but has been supplanted by further SSAs labeled with Y-90 and Lu-177. The latter now has a dominant position in the treatment of NETs following publication of the NETTER-1 study (7), the first randomised control trial (RCT) of peptide receptor radionuclide therapy (PRRT). This study supported a large number of single institutional but largely retrospective clinical reports of the use of PRRT (8), generally given as a treatment of last resort under compassionate-use indications. The application of PRRT has now extended beyond low-grade NETs into higher-grade malignancies with neuroendocrine features (9), including small cell lung cancer (10) and Merkel cell carcinoma as well as for other cancers that express this target, including neuroblastoma (11) and PCC/PGL (12). The combination of Ga-68 DOTA-octreotate and Lu-177 DOTA-octreotate now represents a widely accepted theranostic paradigm. The concept of receptor-based Ga-68/Lu-177 theranostics has more recently been applied to advanced metastatic castrate resistant prostate cancer (mCRPC) through identification of the high expression of prostate-specific membrane antigen (PSMA) in this condition (13). Ga-68 PSMA-11 and Lu-177 PSMA-617 have now been widely evaluated in mCRPC. Although, again, largely within retrospective compassionate-use trials, a recent prospective trial with rigorous eligibility criteria and standardised response assessment confirmed the efficacy and safety of this approach (14) and a prospective, multi-institutional RCT in Australia and another large international RCT are being performed using this paradigm. An important lesson in the development of theranostics has been the recognition of the impact of disease heterogeneity on treatment outcomes (15). With tumour evolution, expression of the theranostic target can be lost. This is generally associated with more aggressive disease biology as a consequence of dedifferentiation. Accordingly, such tumour deposits generally become more metabolically-active and can be identified by F-18 fluoro-deoxyglucose (FDG) PET (16). Thus, FDG PET/CT is increasingly being used in the assessment thyroid cancer, NETs and mCRPC, particularly when lesions identified on CT or MRI have low or no uptake of the theranostic agent. The presence of FDG-avid but target-negative disease has both prognostic and therapeutic implications. On the background of these successes, increasingly sophisticated integration of theranostics into mainstream oncological therapy is in progress. This includes efforts to manipulate target expression pharmacologically, for example with the use of mitogen-activated kinase (MAPK) pathway inhibitors in non-iodine-avid thyroid cancer (17). Improved prescription of administered activity through dosimetry estimation and combination of radionuclide therapy with radiosensitizing chemotherapy, DNA-repair modifying agents or immune check-point mediators are all being actively investigated in the expectation of further enhancing patient outcomes. Even without these innovations, the theranostic paradigm has proven highly effective and very well tolerated in a group of patients that has often failed a large number of prior treatments and typically has poor physical and physiological reserves. These results are encouraging further investment in identification of additional tractable targets for theranostic application and refinement of the choice of radionuclides and targeting agents for both diagnostic and therapeutic application. The wide range of physical characteristics of radioisotopes provides a rich opportunity for tailoring treatment to the kinetics of the targeting agent and the size of disease deposits in individual patients and disease types. High and low-energy beta, alpha and Auger electron emitting radionuclides will likely all find application in the future. Variations in the kinetics of biodistribution between peptides and larger biomolecules including antibodies and antibody fragments, as well as differences in the affinity of agonists and antagonists and the impact of cell internalisation of targeting agents will likely also influence choice of the optimal therapeutic agent. Combination of theranostic agents to achieve increased radiation dose to tumour while distributing off-target radiation to normal tissues is also an attractive area for development. The advances are likely to see greater penetration of theranostics into the treatment of cancer and emphasise the complementary role of molecular imaging to CT and MRI in characterizing the disease sites that are being found with ever greater sensitivity by the latter techniques. Nuclear medicine is returning again to its therapeutic roots and will require new training approaches for imaging specialists to become cognizant of these new concepts (18, 19) and multi-disciplinary engagement in advancing radionuclide therapy.

**References**

1. de Hevesy GC. Marie Curie and her contemporaries. The Becquerel-Curie memorial lecture. J Nucl Med. 1984;25(1):116-31.

2. Steinkamp RC, Lawrence JH, Born JL. Long term experiences with the use of P-32 in the treatment of chronic lymphocytic leukemia. J Nucl Med. 1963;4:92-105.

3. Seidlin SM, Marinelli LD, Oshry E. Radioactive iodine therapy; effect on functioning metastases of adenocarcinoma of the thyroid. J Am Med Assoc. 1946;132(14):838-47.

4. Shapiro B, Sisson JC, Eyre P, Copp JE, Dmuchowski C, Beierwaltes WH. 131I-MIBG--a new agent in diagnosis and treatment of pheochromocytoma. Cardiology. 1985;72 Suppl 1:137-42.

5. Shulkin BL, Shapiro B. Current concepts on the diagnostic use of MIBG in children. J Nucl Med. 1998;39(4):679-88.

6. Krenning EP, Kooij PP, Pauwels S, Breeman WA, Postema PT, De Herder WW, et al. Somatostatin receptor: scintigraphy and radionuclide therapy. Digestion. 1996;57 Suppl 1:57-61.

7. Strosberg J, El-Haddad G, Wolin E, Hendifar A, Yao J, Chasen B, et al. Phase 3 Trial of 177Lu-Dotatate for Midgut Neuroendocrine Tumours. N Engl J Med. 2017;376(2):125-35.

8. Hicks RJ, Kwekkeboom DJ, Krenning E, Bodei L, Grozinsky-Glasberg S, Arnold R, et al. ENETS Consensus Guidelines for the Standards of Care in Neuroendocrine Neoplasia: Peptide Receptor Radionuclide Therapy with Radiolabeled Somatostatin Analogues. Neuroendocrinology. 2017.

9. Thang SP, Lung MS, Kong G, Hofman MS, Callahan J, Michael M, et al. Peptide receptor radionuclide therapy (PRRT) in European Neuroendocrine Tumour Society (ENETS) grade 3 (G3) neuroendocrine neoplasia (NEN) - a single-institution retrospective analysis. Eur J Nucl Med Mol Imaging. 2018;45(2):262-77.

10. Lewin J, Cullinane C, Akhurst T, Waldeck K, Watkins DN, Rao A, et al. Peptide receptor chemoradionuclide therapy in small cell carcinoma: from bench to bedside. Eur J Nucl Med Mol Imaging. 2015;42(1):25-32.

11. Kong G, Hofman MS, Murray WK, Wilson S, Wood P, Downie P, et al. Initial Experience With Gallium-68 DOTA-Octreotate PET/CT and Peptide Receptor Radionuclide Therapy for Pediatric Patients With Refractory Metastatic Neuroblastoma. J Pediatr Hematol Oncol. 2016;38(2):87-96.

12. Kong G, Grozinsky-Glasberg S, Hofman M, Callahan J, Meirovitz A, Maimon O, et al. Efficacy of Peptide Receptor Radionuclide Therapy (PRRT) for Functional Metastatic Paraganglioma and Phaeochromocytoma. The Journal of Clinical Endocrinology & Metabolism. 2017.

13. Afshar-Oromieh A, Malcher A, Eder M, Eisenhut M, Linhart HG, Hadaschik BA, et al. PET imaging with a [68Ga]gallium-labelled PSMA ligand for the diagnosis of prostate cancer: biodistribution in humans and first evaluation of tumour lesions. Eur J Nucl Med Mol Imaging. 2013;40(4):486-95.

14. Hofman MS, Violet J, Hicks RJ, Sandhu S. [(177)Lu]-PSMA-617 radionuclide treatment in patients with metastatic castration-resistant prostate cancer (LuPSMA trial): a single-centre, single-arm, phase 2 study. Lancet Oncol. 2018;19(8):e373.

15. Hicks RJ. Use of molecular targeted agents for the diagnosis, staging and therapy of neuroendocrine malignancy. Cancer Imaging. 2010;10 Spec no A:S83-91.

16. Garin E, Le Jeune F, Devillers A, Cuggia M, de Lajarte-Thirouard AS, Bouriel C, et al. Predictive value of 18F-FDG PET and somatostatin receptor scintigraphy in patients with metastatic endocrine tumours. J Nucl Med. 2009;50(6):858-64.

17. Pattison DA, Solomon B, Hicks RJ. A New Theranostic Paradigm for Advanced Thyroid Cancer. J Nucl Med. 2016;57(10):1493-4.

18. Mankoff D, Pryma DA. Nuclear Medicine Training: What Now? J Nucl Med. 2017;58(10):1536-8.

19. Hicks RJ, Freudenberg L, Beyer T. A new model for training in hybrid imaging. Lancet Oncol. 2018;19(9):1152-4.

## 11:00 – 13:00 Imaging in the Era of Precision Medicine

### A36 Malignant Bone Disease

#### C Messiou^1,2^ (Christina.Messiou@rmh.nhs.uk)

##### ^1^Department of Radiology, The Royal Marsden NHS Foundation Trust, Fulham Rd, London, UK; ^2^Division of Radiotherapy and Imaging, The Institute of Cancer Research, 15 Cotswold Rd, Sutton, UK

Conventional imaging techniques are failing to accurately define the presence, volume, viability and response of malignant bone disease which is a prerequisite for precision medicine approaches.

Standard oncological staging with CT lacks sensitivity and for sclerotic disease fails to differentiate healing flare response from true progression. In the absence of soft tissue elements, metastatic bone disease remains non-measureable by RECIST 1.1 [1]. This is particularly problematic in cancers such as breast and prostate where metastases occur preferentially or exclusively in bone. Current imaging also lacks sufficient diagnostic accuracy to guide metastasis directed therapy for oligometastatic disease. These therapies potentially shorten or postpone the use of systemic treatment and can delay further metastatic progression with potential to influence overall survival [2]. Durable responses now seen in immunotherapy represent a major advancement in patient care but with significant potential for severe toxicity, robust patient selection and monitoring is paramount.

In multiple myeloma the benefits of more advanced imaging in the form of whole body MRI are perhaps more widely accepted having been incorporated into International Myeloma Working Group Guidance and UK NICE guidance [3,4]. The benefits of early diagnosis, impact on quality of life and positive health economic analyses have been highly influential in the rise of whole body MRI. International consensus (MY-RADS) has reinforced diffusion weighted imaging as a core component of the protocol which has positioned whole body MRI as a leading imaging tool for guiding precision medicine approaches [5]. Similar consensus guidelines on whole body MRI for metastatic prostate cancer (MET-RADS-P) is further advancing acceptance and utilisation [6].

For both metastatic and myeloma bone disease, deficiencies in the ability of serum biomarkers to reflect disease status particularly in later stages of the disease strengthens the need for quantitative whole body imaging. Targeted biopsy or posterior iliac crest trephine are not only invasive but prone to sampling errors. Parallel advances in imaging and informatics are allowing progress from single site biopsy and phenotyping to phenotypic probabilities of multiple sites over multiple time points. This becomes increasingly relevant as our insight into spatial heterogeneity of bone disease evolves. In multiple myeloma spatial genomic heterogeneity occurs in more than 75% of patients and is also positively associated with the size of biopsied focal lesions consistent with regional outgrowth of advanced clones [7].

In this session we will explore use of imaging in malignant bone disease to direct the right treatment, to the right patient, at the right time and for the right duration.

**References**

1. Eisenhauer EA, Therasse P, Bogaerts J et al. New response evaluation criteria in solid tumours: revised RECIST guideline (version 1.1). EJC 2009;45:228-47.

2. Lecouvet F, Oprea-Lager D, Liu Y et al. Use of modern imaging methods to facilitate trials of metastasis-directed therapy for oligometastatic disease in prostate cancer: a consensus recommendation from the EORTC Imaging Group. Lancet Oncology 2018;19:e435-e454.

3. Dimopoulos M, Hillengass J, Usmani, S et al. Role of Magnetic Resonance Imaging in the Management of Patients with Multiple Myeloma: A Consensus Statement. Journal of Clinical Oncology 2015; 6: 657-664.

4. Myeloma Diagnosis and Management. NICE (NG35). https://www.nice.org.uk/guidance/ng35

5. Messiou C, Hillengass J, Delorme S et al. Guidelines for Acquisition, Interpretation, and Reporting of Whole-Body MRI in Myeloma: Myeloma Response Assessment and Diagnosis System (MY-RADS). Radiology 2019; 291:5-13

6. Padhani AR, Lecouvet FE, Tunariu N, et al. Metastasis Reporting and Data System for Prostate Cancer: Practical guidelines for acquisition, interpretation, and reporting of whole-body magnetic resonance imaging-based evaluations of multiorgan involvement in advanced prostate cancer. Eur Urol 2017;71(1):81–92.

7. Rasche L, Chavan S, Stephens O et al. Spatial genomic heterogeneity in multiple myeloma revealed by multi-region sequencing. Nature Communications 2017; 8:268.

### A37 Immunotherapy: Imaging evaluation of response and toxicities from immune-checkpoint inhibitors

#### M. Nishino

##### Dana-Farber Cancer Institute, Boston, MA, USA

Immunotherapy for cancer using immune-checkpoint inhibitors has become a major treatment option for various advanced cancers, and has brought a paradigm shift in therapeutic approaches to cancer patients [1-2]. The mechanism of immune-checkpoint inhibitor therapy is via the blockade of immune inhibition by tumours, which are associated with novel types of tumour response patterns and toxicities. Imaging plays a critical role in objectively characterizing immune-related tumour responses and progression, and in detecting and monitoring immune-related toxicities [1-2]. Given a rapidly increasing use of cancer immunotherapy in the clinical setting, it is essential for radiologists to be familiar with the current status of immunotherapy, cutting-edge approaches for immune-related response and toxicity evaluations, and the limitations and pitfalls of the current strategies.

In some patients treated with immune-checkpoint inhibitors, unconventional tumour response patterns have been noted on imaging, including 1) response after an initial increase of tumour burden, and 2) response during or after appearance of new lesions [1-3]. These patterns are often termed “pseudoprogression”, because they meet the criteria for disease progression by the conventional tumour response criteria, such as RECIST, based on the initial increase or appearance of new lesions. The underlying mechanism of pseudoprogression is thought to be the infiltration of T cells into tumours resulting in in initial apparent increase of tumour burden. Several modified criteria have been developed to capture these unconventional patterns, including irRC, irRECIST, and iRECIST [1, 2, 4-6].

Although the phenomenon of pseudoprogression is often featured as a representative immune-related response pattern, it is also important to note that the overall incidence of pseudoprogression in patients treated with immune-checkpoint inhibitors is low, commonly only up to 5-10% or less [1-2]. Therefore, the accumulating data suggest that tumour burden increase during immune-checkpoint inhibitor therapy indicates true progression in most patients rather than pseudoprogression. Therefore, radiologists have an important role to objectively assess imaging findings and have active dialogues with referring clinicians, in order to contribute to optimal patient care.

Furthermore, the emerging data indicate the importance of evaluating the longitudinal tumour burden dynamics on serial imaging studies during therapy, which helps to provide a practical marker for survival and treatment benefits in patients treated with immune-checkpoint inhibitor therapy [7]. Another recently described pattern is known as hyperprogressive disease, which indicates the early and rapid tumour progression with an accelerated rate of tumour growth after stating immune-checkpoint inhibitor therapy [8]. These observations indicate the complexity of immune-related tumour responses and emphasise the needs for further investigations for biomarker and imaging marker development.

Immune-checkpoint inhibitor therapy is also associated with unique toxicities, termed immune-related adverse events (irAEs), which can involve various organs from head to toe. The irAEs present a spectrum of imaging manifestations in an organ-specific manner [1, 9-12]. In many irAEs that involve major organs, radiologists have important roles for early detection, accurate diagnosis, and monitoring during clinical management as a part of multidisciplinary team for cancer care.

**References**

1. Nishino M, Hatabu H, Hodi FS. Imaging of Cancer Immunotherapy: Current Approaches and Future Directions. Radiology. 2019;290(1):9-22.

2. Nishino M, Ramaiya NH, Hatabu H, Hodi FS. Monitoring immune-checkpoint blockade: response evaluation and biomarker development. Nature reviews Clinical oncology. 2017;14(11):655-68.

3. Nishino M, Jagannathan JP, Krajewski KM, O'Regan K, Hatabu H, Shapiro G, et al. Personalised tumour response assessment in the era of molecular medicine: cancer-specific and therapy-specific response criteria to complement pitfalls of RECIST. AJR American journal of roentgenology. 2012;198(4):737-45.

4. Nishino M, Giobbie-Hurder A, Gargano M, Suda M, Ramaiya NH, Hodi FS. Developing a common language for tumour response to immunotherapy: immune-related response criteria using unidimensional measurements. Clinical cancer research : an official journal of the American Association for Cancer Research. 2013;19(14):3936-43.

5. Seymour L, Bogaerts J, Perrone A, Ford R, Schwartz LH, Mandrekar S, et al. iRECIST: guidelines for response criteria for use in trials testing immunotherapeutics. The Lancet Oncology. 2017;18(3):e143-e52.

6. Wolchok JD, Hoos A, O'Day S, Weber JS, Hamid O, Lebbe C, et al. Guidelines for the evaluation of immune therapy activity in solid tumours: immune-related response criteria. Clinical cancer research : an official journal of the American Association for Cancer Research. 2009;15(23):7412-20.

7. Nishino M, Dahlberg SE, Adeni AE, Lydon CA, Hatabu H, Janne PA, et al. Tumour Response Dynamics of Advanced Non-small Cell Lung Cancer Patients Treated with PD-1 Inhibitors: Imaging Markers for Treatment Outcome. Clinical cancer research : an official journal of the American Association for Cancer Research. 2017;23(19):5737-44.

8. Champiat S, Dercle L, Ammari S, Massard C, Hollebecque A, Postel-Vinay S, et al. Hyperprogressive Disease Is a New Pattern of Progression in Cancer Patients Treated by Anti-PD-1/PD-L1. Clinical cancer research : an official journal of the American Association for Cancer Research. 2017;23(8):1920-8.

9. Nishino M, Sholl LM, Hodi FS, Hatabu H, Ramaiya NH. Anti-PD-1-Related Pneumonitis during Cancer Immunotherapy. The New England journal of medicine. 2015;373(3):288-90.

10. Nishino M, Giobbie-Hurder A, Hatabu H, Ramaiya NH, Hodi FS. Incidence of Programmed Cell Death 1 Inhibitor-Related Pneumonitis in Patients With Advanced Cancer: A Systematic Review and Meta-analysis. JAMA oncology. 2016; 2(12):1607-1616.

11. Nishino M, Ramaiya NH, Awad MM, Sholl LM, Maattala JA, Taibi M, et al. PD-1 Inhibitor-Related Pneumonitis in Advanced Cancer Patients: Radiographic Patterns and Clinical Course. Clinical cancer research : an official journal of the American Association for Cancer Research. 2016;22(24):6051-60.

12. Tirumani SH, Ramaiya NH, Keraliya A, Bailey ND, Ott PA, Hodi FS, et al. Radiographic Profiling of Immune-Related Adverse Events in Advanced Melanoma Patients Treated with Ipilimumab. Cancer immunology research. 2015;3(10):1185-92.

### A38 Abdominal and pulmonary complications of immunotherapy

#### Richard M. Gore, Kiran H. Thakrar, Daniel R. Wenzke, Silvers RI

##### Department of Radiology, North Shore University Health System-University of Chicago, Evanston, IL, USA

###### **Correspondence:** Richard M. Gore (rgore@uchicago.edu)

**Introduction:** Over the last 15 years, the management of cancer patients has been revolutionised by the advances in molecular targeted therapy and immunotherapy with significant benefits for patient outcomes and comfort. These therapies however are associated with new toxicities and complications that: can be mild, moderate or life-threatening; may require alteration or cessation of therapy; or simulate disease progression. In this presentation the various classes of immunotherapy associated with thoracic and abdominal complications are reviewed and the drug-associated injuries and their differential diagnosis are presented.

**Hepatitis:** Hepatitis following immunotherapy is typically detected on routine serum liver function tests. Other causes of liver damage such as viral infection, alcohol, other medications or cancer progression need to be excluded. Sinusoidal obstruction syndrome, Budd-Chiari syndrome and portal vein thrombosis should also be excluded. On imaging, immunotherapy associated hepatitis manifests with non-specific and variable findings according to clinical severity: hepatomegaly, edema and enlarged lymph nodes in the periportal region. Liver biopsy, only necessary in complicated cases, may reveal predominantly hepatocyte injury (acute hepatitis pattern) with sinusoidal histiocytic infiltrates, central hepatic vein damage and endothelial inflammation similar to autoimmune hepatitis, or predominant bile duct injury (biliary pattern, with portal inflammation).

**Colitis:** Two distinct patterns of anti-CTLA-4-associated colitis have been observed on CT: a more common diffuse colitis characterised by mesenteric vessel engorgement, and a segmental colitis with moderate wall thickening and associated pericolonic fat stranding in a segment of preexisting diverticulosis. Colonoscopy is the most accurate means of evaluating the extent and severity of colitis and is recommended in appropriate cases since recent data suggest that the presence of ulceration on endoscopy predicts steroid-refractory disease. For grade ≥ 2 diarrhea, systemic immunosuppression should be initiated promptly after ruling out infectious etiology.

**Pneumonitis:** Drug induced pneumonitis develops in up to 10% of patients on immunotherapy and remains a diagnosis of exclusion that must be differentiated from infection and malignant lung infiltration. Five different patterns have been described on CT: ground glass opacities with preserved bronchovascular markings; increased interstitial markings, interlobular septal thickening, peribronchovascular infiltration, subpleural reticulation, and honeycomb pattern in severe cases ; cryptogenic organizing pneumonia-like, with discrete patchy or confluent consolidation with or without air bronchograms, predominantly peripheral or subpleural in location; non-specific, with a mixture of nodular and other subtypes, not clearly fitting into other subtype classifications.

**Bronchiolitis Obliterans:** There is myxoid fibrous tissue filling the distal bronchioles and extending into alveolar ducts and associated with inflammatory cells. On CT imaging findings include: bilateral regions of patchy consolidation or small irregular nodular opacities, bronchial wall thickening and dilation, and small pleural effusions.

**Radiation Recall Pneumonitis:** This is an inflammatory reaction in previously irradiated areas of lung producing well defined areas of alveolar consolidation, ground glass opacities or infiltrates corresponding to the radiation portals. This pneumonitis usually presents 3-4 months following radiotherapy and the patient presents with cough and dyspnea.

**Pulmonary Veno-Occlusive Disease**: Progressive occlusion of postcapillary pulmonary venules leads to increased pulmonary resistance, pulmonary hypertension, and right ventricular failure. CT findings include diffuse ground-glass opacification, septal thickening, peribronchial thickening, soft tissue oedema around the hila and mediastinum, small pleural effusions, and dilatation of the central pulmonary arteries.

**Sarcoid-Like Granulomatous Reactions:** Intrathoracic lymphadenopathy simulating sarcoidosis develops in up to 10% of patients following ipilimuab and nivolumab therapy. The adenopathy may manifest and newly enlarged lymph nodes or enlargement of pre-existing lymph nodes that occur in isolation or associated with bilateral upper lobe and middle lobe predominant ground glass opacities, parenchymal consolidations and/or irregular nodules. Most patients are asymptomatic and biopsy show non-caseating granulomas with elevated CD4:CD8 levels. Extrathoracic diffuse adenopathy and cutaneous non-caseating granulomas have also been described.

**Pseudoprogression:** Immunotherapy often may initially provoke infiltration of cytotoxic T lymphocytes and other immune cells into the tumour bed. This may cause an increase in tumour size or the development of new lesions as an early response. Pseudoprogression is defined as ≥ 25% increase in tumour burden that is not seen on repeat imaging performed 4 weeks or more after the initial study. Mixed immune-related responses or pseudoprogression are quite problematic in assessing treatment response using RECIST criteria.

This abstract has been previously published.

**References**

1. Nishino M, Hatabu H, Hodi FS: Imaging of Cancer Immunotherapy: Current Approaches and Future Directions. Radiology 2018; 290: 9-22.

2. Kwak JJ, Tirumani SH, Van den Abbeele AD, et al: Cancer Immunotherapy: Imaging Assessment of Novel Treatment Response Patterns and Immune-related Adverse Events. RadioGraphics 2015; 35: 424-437.

3. Wang GX, Kurra V, Gainor JF, et al: Immune Checkpoint Inhibitor Cancer Therapy: Spectrum of Imaging Findings. RadioGraphics 2017; 37: 2132-2134.

4. Krajewski KM, Braschi-Amirfarzan M, DiPiro PJ, Jagannathan JP, Shinagare AB: Molecular Targeted Therapy in Modern Oncology: Imaging Assessment of Treatment Response and Toxicities. Korean J Radiol 2017; 18: 28-41.

5. Kroschinsky F, Stolzel F, von Bonin S, et al: **New drugs, new toxicities: severe side effects of modern targeted and immunotherapy of cancer and their management.** *Critical Care* 2017; **21**: 89—100.

6. Tabchi S, Messier C, Blais N: **Immune-mediated respiratory adverse events of checkpoint inhibitors**. *Curr Opin Oncol* 2017; **28:** 269-277.

### A39 Cancer Imaging in Drug Development for Precision Imaging

#### Annick D. Van den Abbeele^1,2,3,4^ (abbeele@dfci.harvard.edu)

##### ^1^Department of Imaging and Center for Biomedical Imaging in Oncology, Dana-Farber Cancer Institute, Harvard Medical School, Boston, MA, USA; ^2^Tumour Imaging Metrics Core, Dana-Farber/Harvard Cancer Center, Harvard Medical School, Boston, MA, USA; ^3^Brigham Health, Harvard Medical School, Boston, MA; ^4^Cancer Imaging, International Cancer Imaging Society, London, United Kingdom

There is still an enormous gap between preclinical studies demonstrating proof-of-principle drug efficacy and successful translation of these novel drugs into daily clinical practice [1]. The process from discovery to regulatory approval is lengthy (up to 15 years), risky and expensive. One key cost driver is the increasing number of failed drug candidates. Reasons for these failures are numerous, but many of these could be mitigated by early and comprehensive incorporation of biomarkers into drug development strategies, as highlighted in a recent report indicating that the use of biomarkers increased success rates at each stage of clinical development [2, 3]. Using a sample of 406,038 entries of clinical trial data for over 21,143 compounds from January 1, 2000 to October 31, 2015, Wong et al estimated aggregate clinical trial success rates and duration [4]. The authors reported that after declining to 1.7% in 2012, success for oncology trials has improved to 2.5% and 8.3% in 2014 and 2015, respectively. In addition, oncology trials that use biomarkers in patient-selection had higher overall success probabilities than trials without biomarkers [4].

Cancer imaging can direct valuable resources toward the development of drugs that are most likely to succeed through the use of routine noninvasive whole body imaging including hybrid imaging technologies, and the inclusion of imaging biomarkers in prospective clinical trials where their objectivity and reproducibility can be tested as primary objectives. These new strategies can help identify promising new drug candidates early, while eliminating those that are unlikely to be successful. With a well-developed biomarker strategy, valuable insights into disease pathogenesis can also be derived from a clinical trial [5], and biomarkers could be validated as companion diagnostics, pharmacodynamic markers, or “virtual biopsies”.

Pharmacodynamic biomarkers can, indeed, demonstrate, that a molecule effectively engages its target early after initiation of therapy. If patients can then be selected appropriately using these predictive biomarkers, a more confident assessment of the link (or lack thereof) between a molecular target and a clinical manifestation can be made [5]. This strategy has been used in several preclinical and clinical trials including trials with molecularly targeted drugs such as imatinib and sunitinib [6-8], as well as others including immunotherapy [9]. Several new opportunities, in the form of co-clinical trials [10], novel trial designs, modeling strategies, longitudinal studies of patients to obtain information about anticancer therapies throughout their life cycle using innovative imaging biomarkers, functional imaging techniques, artificial intelligence, deep learning and radiomics [11] could significantly improve the overall drug development process [12] Being able to assess who to treat and how to match patients to the best clinical trial and the best treatment would also allow for rapid testing in less patients, improve response evaluations, and help choose combination therapies with the greatest likelihood of success, efficacy and safety [13].

Cancer imaging is, indeed, well positioned to make a significant impact on the different phases of drug development, and shorten the timeline between discovery, pivotal preclinical/clinical trials and regulatory approval in a safe, timely and cost-effective manner [14, 15].

**References**

1. Tarkin, J.M., M.R. Dweck, and J.H.F. Rudd, *Imaging as a surrogate marker of drug efficacy in cardiovascular disease.* Heart, 2019. **105**(7): p. 567-578.

2. Townsend, M.J. and J.R. Arron, *Reducing the risk of failure: biomarker-guided trial design.* Nature Reviews Drug Discovery, 2016. **15**: p. 517.

3. David W. Thomas, J.B., John Audette, Adam Carroll, Corey Dow-Hygelund, Michael Hay, *Clinical Development Success Rates: 2006–2015.*

4. Wong, C.H., K.W. Siah, and A.W. Lo, *Estimation of clinical trial success rates and related parameters.* Biostatistics, 2019. **20**(2): p. 273-286.

5. Townsend, M.J. and J.R. Arron, *Reducing the risk of failure: biomarker-guided trial design.* Nat Rev Drug Discov, 2016. **15**(8): p. 517-8.

6. Demetri, G.D., et al., *Efficacy and safety of imatinib mesylate in advanced gastrointestinal stromal tumours.* N Engl J Med, 2002. **347**(7): p. 472-80.

7. Van den Abbeele, A.D. and M. Ertuk, *FDG-PET to Measure Response to Targeted Therapy: The Example of Gastrointestinal Stromal Tumour and Imatinib Mesylate (Gleevec).* PET Clin, 2008. **3**(1): p. 77-87.

8. Demetri, G.D., et al., *Molecular target modulation, imaging, and clinical evaluation of gastrointestinal stromal tumuor patients treated with sunitinib malate after imatinib failure.* Clin Cancer Res, 2009. **15**(18): p. 5902-9.

9. Pandit-Taskar, N., et al., *Biodistribution and Dosimetry of (18)F-Meta-Fluorobenzylguanidine: A First-in-Human PET/CT Imaging Study of Patients with Neuroendocrine Malignancies.* J Nucl Med, 2018. **59**(1): p. 147-153.

10. Chen, Z., et al., *A murine lung cancer co-clinical trial identifies genetic modifiers of therapeutic response.* Nature, 2012. **483**(7391): p. 613-7.

11. Nass, S.J., et al., *Accelerating anticancer drug development - opportunities and trade-offs.* Nat Rev Clin Oncol, 2018. **15**(12): p. 777-786.

12. Willmann, J.K., et al., *Molecular imaging in drug development.* Nat Rev Drug Discov, 2008. **7**(7): p. 591-607.

13. Constance, J. *Pharm Exec's 2019 Pipeline Report*. Pharmaceutical executive 2019 Nov 23,2018 [cited 38 11]; Available from: http://www.pharmexec.com/pharm-execs-2019-pipeline-report.

14. Hricak, H., *2016 New Horizons Lecture: Beyond Imaging-Radiology of Tomorrow.* Radiology, 2018. **286**(3): p. 764-775.

15. Van den Abbeele, A.D., et al., *Cancer Imaging at the Crossroads of Precision Medicine: Perspective From an Academic Imaging Department in a Comprehensive Cancer Center.* J Am Coll Radiol, 2016. **13**(4): p. 365-71.

### A40 Integrating imaging with other “omics” biomarkers

#### Gigin Lin (giginlin@cgmh.org.tw)

##### Department of Medical Imaging and Intervention, Chang Gung Memorial Hospital, Taipei, Taiwan

The purpose of this talk is to discuss how to integrate imaging as a personalised biomarker in the era of precision medicine. Advance in biotechnology brings precision medicine into clinical reality. Molecular diagnostics applied to blood samples or tumour tissues can measure changes in genomics, proteomics, or metabolomics at the individual cell level. Next generation sequencing enables high-throughput gene profiling, while mass spectrometry can detect thousands of proteins or metabolites. Imaging, on the other hand, detects more clinically significant disease and provides valuable information regarding tumour characteristics such as location and extension to guide surgical planning [1]. Imaging can also fill these knowledge gaps between biospecimen by providing complementary information on tumour characteristics, including heterogeneity and the microenvironment, as well as on pharmacokinetic parameters, drug–target engagement and responses to treatment. Probing molecular changes will aid not only cancer diagnosis, but also provide tumour grading, based on gene-expression analysis and imaging measurements of cell proliferation and changes in metabolism; staging, based on imaging of metastatic spread and elevation of protein biomarkers; and the detection of therapeutic response, using serial molecular imaging measurements or monitoring of serum markers. This integrative approach could therefore streamline biomarker and drug development, although a range of issues needs to be overcome in order to enable broader use of molecular imaging in clinical trials [2].

References

1. Lin G, Keshari KR, Park JM. Cancer Metabolism and Tumour Heterogeneity: Imaging Perspectives Using MR Imaging and Spectroscopy. Contrast Media Mol Imaging. 2017;2017:6053879.

2. Lin G, Lai CH, Yen TC. Emerging Molecular Imaging Techniques in Gynecologic Oncology. PET Clin. 2018;13:289-99.

### P1 Atypical imaging presentations of lung cancers on CT

#### Khin YT, Chawla A

##### Khoo Teck Puat Hospital, Singapore

###### **Correspondence:** Khin YT (kythein@yahoo.com)

Learning Objectives

To understand atypical presentation of lung cancer on CT scan.

Content Organisation

The most common presentation of lung cancer on CT scan is of a solid nodule or a mass. However, increasing cases of atypical appearance of lung cancer is being realised amongst the radiologists. Apart from solid nodule or mass, lung cancer should be suspected in following situation such as bubbly mass, ground glass density nodule or mass, solid mass with pseudocavitation, cystic mass with solid component, persistent mucus plug in airways, endobronchial nodule, crazy paving pattern with nodules, isolated collapse of pulmonary segments or lobe and persistent focal consolidation.

We will present the spectrum of imaging findings of these atypical presentation of lung cancer on CT.

Conclusion

Early diagnosis of lung cancer is essential to provide adequate treatment and improve the mortality. Understanding of atypical appearance of lung cancer on imaging can help in early work-up of the patient with lung cancer.

### P2 Peritoneal malignancies: imaging in different modalities and a proposed selection framework

#### Hennedige T, Nazir B

##### National Cancer Centre Singapore, Singapore

###### **Correspondence:** Hennedige T (hennedige.tiffany.priyanthi@singhealth.com.sg)

Learning Objectives

To review mechanisms of spread within the peritoneum and the advantages and shortcomings of using CT, MRI and PET for the detection of peritoneal deposits

Content Organisation

Review of peritoneal anatomy and mechanisms of spread of malignancy including intraperitoneal dissemination, direct spread along peritoneal pathways and haematogeneous tumour dissemination.

Peritoneal imaging for malignancy remains relatively challenging, this is compounded by the variety of imaging modalities available.

MR protocol and interpretation including diffusion weighted imaging and fat-suppressed dynamic contrast-enhanced imaging.

Benefits and limitations of using CT, MRI and PET in the detection of peritoneal deposits in varying peritoneal malignancies.

Side-by-side pictorial comparison of the detection of peritoneal nodules using varying imaging modalities in different peritoneal malignancies.

A proposed selection framework of the appropriate imaging modality for the peritoneal malignancy in question.

Conclusion

Imaging of peritoneal malignancies is relatively challenging, and the availability of varying imaging modalities compounds the problem. This proposed framework may aid the clinician in selecting the appropriate imaging modality for the malignancy in question.

### P3 Gastrointestinal stromal tumours: a comprehensive review of clinical, imaging and pathological features

#### Weston B, Douek M, Raman S, Kadell B, Patel M

##### University of California Los Angeles, Los Angeles, California, USA

###### **Correspondence:** Weston B (westonbr89@gmail.com)

Learning Objectives

This exhibit will provide a comprehensive review of the imaging appearance of gastrointestinal stromal tumours (GISTs) with an emphasis on the imaging of treatment response. The radiologist’s role in assessing treatment response will be discussed and correlated with treatment strategies.

Content Organisation

GISTs are mesenchymal neoplasms that can present throughout the entire gastrointestinal tract, from the esophagus through the rectum. Though they comprise less than 1% of primary gastrointestinal tumours, the 5-year survival rate is greater than 75%, and patients require frequent imaging surveillance. This exhibit will discuss the epidemiology, pathologic grading and clinical presentation of GISTs with correlation to distribution in the stomach, small and large bowel. The imaging appearance of primary and metastatic lesions on CT and MRI will be reviewed. Tumour staging, including RECIST, iRecist and Choi criteria will be discussed. The exhibit will provide an update of treatment strategies including chemotherapy, immunotherapy, surgery, and ablation with imaging correlates for treatment response.

Conclusion

Radiologists play an important part in the management of GISTs, from initial diagnosis, to therapy and long term surveillance. Oncologic imagers must be familiar with tumour appearance on multiple imaging modalities and the different classification schemes for staging disease. Knowledge of the current treatment regimens and expected post-treatment changes allows the radiologist to provide meaningful patient care.

### P4 Set lasers to cure: radiology’s role in laser ablation surgery for brain tumours

#### Weston B, Salamon N

##### University of California Los Angeles, Los Angeles, California, USA

###### **Correspondence:** Weston B (westonbr89@gmail.com)

Learning Objectives

Laser interstitial thermal therapy is a minimally invasive surgical technique which allows for precise targeting of small lesions that are not amenable to surgical excision. Radiologists play an integral role in pre-surgical planning and providing follow-up imaging to assess treatment response. Understanding the indications for treatment, the basics behind magnetic resonance thermometry, and expected post-ablation changes, can help the radiologist provide critical information to the clinical team.

Content Organisation

This presentation will demonstrate a comprehensive review of the radiologist’s role in laser interstitial thermal therapy ranging from pre-treatment planning to post-ablation monitoring. The indications for laser ablation therapy (including primary CNS neoplasms, hypothalamic hamartomas, and metastatic disease) will be reviewed. Advanced imaging techniques (MRI thermometry with real time monitoring, diffusion tensor imaging and 3D reformatting) and their utility in target delineation will be discussed. Sample cases will familiarise the radiologist with key findings on intra-operative imaging and post-ablation follow-up.

Conclusion

Laser interstitial thermal therapy is an effective way to treat diseases located in deep structures which are not amenable to surgical excision or radiation therapy. As the role of this treatment evolves, radiologists play a critical role in pre-surgical planning and monitoring disease response/progression. A strong understanding of neuroanatomy and magnetic resonance imaging allows the radiologist to maximise the patient’s chance at a successful outcome.

### P5 Efficacy of imaging modalities in detecting nasopharyngeal carcinoma extension to the inferior orbital fissure

#### Chee K Kee^1^, Faimee E M Nor^1^, James B K Khoo^2^

##### ^1^National University Hospital, Singapore; ^2^National Cancer Centre, Singapore

###### **Correspondence:** Chee K Kee (chee_kwang_kee@nuhs.edu.sg)

Aim

The inferior orbital fissure (IOF) is a potential pathway for orbital and intracranial extension of nasopharyngeal carcinoma (NPC). In univariate analysis, orbital or intracranial involvement by NPC confer bad prognosis. In this study we are comparing the efficacy of PET-CT, contrast-enhanced CT (CECT) and MRI in detecting extension of NPC to IOF.

Materials and Methods

A total of 78 patients with histological-proven NPC whose underwent MRI nasopharynx (Signa Echospeed, GE), PET-CT (Siemens Biograph LSO unit) and CECT were enrolled. MRI and CECT images were read independently by two diagnostic radiologists respectively. PET-CT studies were read by a nuclear medicine specialist. The images were examined for possible tumour extension to the IOF and beyond this, orbital and/or intracranial extension.

Results

MRI detected all 10 patients (100%) with NPC with IOF involvement. CECT detected 7 (70%) and PET-CT detected 4 (40%) of the patients, respectively. There were 3 patients whose IOF disease was detected only on MRI but not on PET-CT or CECT. 2 of 10 (20%) patients also had extension of disease into the orbit. 8 of 10 (80%) patients had intracranial extension.

Conclusions

Although PET-CT has been shown to be more accurate in detecting distant metastasis in NPC, it is less accurate in the staging of local disease, in particular, disease extending to the IOF. Of the 3 modalities, MRI is superior in the detection of IOF disease due to its superior contrast resolution.

### P6 Breast suspicious microcalcifications; Diagnostic Accuracy of Ultrasound-guided Core Needle Biopsy

#### Asmaa Abdel Magied^1^, Lamiaa Salah-Aldin^1^, Rasha Wessam^1^, Omar Omar^2^

##### ^1^Department of Radiodiagnosis, Faculty of Medicine, Cairo University, Cairo, Egypt; ^2^Department of Surgery, Faculty of Medicine, Cairo University, Cairo, Egypt

###### **Correspondence:** Asmaa Abdel Magied (asmaa_dr79@hotmail.com)

Aim

Evaluate the accuracy of ultrasound guided core needle biopsy and rate of successful retrieval for target sampling of sonographicallydetected suspicious microcalcifications in solid lesions

Material and methods

Evaluation of 30 female patients with 32 lesions presenting with suspicious microcalcifications on mammography and sonography wasdone. The age ranged from 21 to 75 years (mean= 48.53). All of the patients were subjected to digital mammography, B-mode ultrasound examination and ultrasound guided 14-G semi-automated core biopsy(CNB). Specimen radiographs were done and tissue samples were sent to histopathology. 30/32 lesions underwent surgical excision and histopathology.

Results

Calcifications were successfully retrieved in 30/32 lesions (93.8 %) and confirmed on specimen radiography. Failure of calcification retrieval was encountered in two lesions (2/32) (6.3%). 26/30 lesions (86.7%) were proved malignant based on surgical excision and final pathology reports (22 IDC, 2 DCIS, 2 ILC) and 4 out of the 30 lesions were benign (two ADH, one fibroadenoma, and one fibroadenosis). The total number of retrieved cores in the successful retrieval group ranged from six to 11(mean=7.73), in the failed retrieval group (two lesions); it ranged from seven to 12 (mean=9.5). In the successful retrieval group, the mean number of cores containing calcification ranged from two to nine (mean= 5.50). The overall accuracy of US-guided 14-G semi-automated CNB was 90.0 % (27/32).

Conclusion

US-guided 14-G semi-automated CNB is useful procedure for sonographically detected suspicious microcalcifications. Specimen radiography should be done in all cases to confirm the accurate retrieval of microclcifications by core biopsy.

### P7 Interactive, up-to-date meta-analysis: A novel format to bring the latest evidence to patients and practitioners

#### A.S. Becker, S. Woo, S. Ghafoor, H. Hricak, H.A. Vargas

##### Memorial Sloan Kettering Cancer Center

###### **Correspondence:** A.S. Becker (anton.s.becker@gmail.com)

Introduction

Systematic reviews (SR) and meta-analyses of randomised controlled clinical trials represent the highest level of evidence in evidence based medicine. However, traditional scientific publishing essentially separates the underlying raw data from the published review, where the data is typically coerced in the form of a few graphs and summary tables. The heterogeneity of study populations and study designs, together with the rigid display may limit the applicability of SR to the single use case.

Learning Objectives

1. The fundamental principles and most important metrics of meta analyses are reviewed on the example of Multiparametric vs. biparametric MRI in the detection of prostate cancer
2. The reader will become familiar with the new format “iu-ma”, which allows for
  - continuous updating of the meta-analysis as new eligible studies are published
  - personalised selection of study characteristics included in the analysis and interactive display of results tailored the individual treatment center or patient

Methods

The statistical programming language R, together with the RStudio and shiny software, meld data and real-time graphical presentation thus enabling interactive exploration of a dataset by the user. Interactive, up-to-date meta-analysis (iu-ma) is a novel format of displaying data from a systematic review and let the user chose to customise the display of the relevant results.

Conclusion

The iu-ma format directly connects figures and tables with the underlying data, thus allowing for dynamic presentation of relevant subsets of the data as well as facilitated updating of the data as new original studies are continuously published.

### P8 Incidence of contrast-induced acute kidney injury (CI-AKI) in high-risk oncology patients undergoing contrast-enhanced-CT with iodixanol

#### M. Horger, S. Werner

##### Eberhard-Karls-University Tuebingen, Department of Radiology, Tuebingen, Germany

###### **Correspondence:** M. Horger (marius.horger@med.uni-tuebingen.de)

Aim

Determine the incidence of contrast-induced acute kidney injury (CI-AKI) [increase in serum creatinine(SCr)≥0.5mg/dl or≥25% over the baseline value/decrease in eGFR(MDRD)≥25% (Definition 1) or increase in SCr≥0.3 mg/dl or ≥50%(Definition 2)] within 1 week following i.v. iodixanol administration.

Materials and methods

Retrospective analysis of 252 high-risk oncology patients (mean age, 70y) presenting primarily with restricted renal function (eGFR<60mL/min/1.73m²) undergoing 345 CECT with iodixanol. Median-baseline SCr and eGFR were: 1.4mg/ml (range,0.9-3.2) and 47.2ml/min/1.73m² (range,14.6-59.9ml/min/1.73m²). All patients received 60 ml iodixanol followed by 30 ml NaCl. Oral hydration with 500 ml water before and after CECT was provided. Following risk factors: post-renal transplantation/post-stem cell transplantation/diabetes mellitus/arterial hypertension/peripheral artery disease[PAD]/congestive heart failure[CHF]/nephrotoxic medication including concomitant chemotherapy for PC-AKI were evaluated.

Results

Overall post-contrast AKI (causal and coincidental role of contrast agent for AKI) incidence was 4.9% (Definition 1) and 7.8% (Definition 2). Excluding concurrent medical conditions resulted in a CI-AKI incidence of 3.8% and 6.2%, respectively. Subgroups considering baseline eGFR yielded incidences of 5.1% and 6.1 % (eGFR>45-60ml/min/1.73m²), 4.6% and 9.9% (eGFR 30-45 ml/min/1.73m²) and 6.3% and 12.5 % (eGFR<30 ml/min/1.73m²), respectively. No patient required renal replacement therapy. There were no statistically significant differences regarding the median absolute and relative change pre- and post-CECT in eGFR (p=0.3) and SCr (p=0.2) in subgroups with a baseline eGFR of <30/30-4/45-60 ml/min/1.73m². Only CHF and PAD were independently associated with the occurrence of CI-AKI.

Conclusion

The overall incidence of CI-AKI was 3.8% and 6.2%, respectively, depending on the diagnostic criteria used. The use of a reduced-dose iso-osmolar contrast medium is safe in high-risk oncologic patients.

### P9 Optimisation of Hepatocellular Carcinoma CT Imaging across UK NHS Trusts

#### A. Sklavounos, D. Scotto, S. Howard-Walker, I. Gonzalez Douglas, S. Gregory, A. Koundouraki, S. Khorsandi

##### Department of Women and Children's Health, King's College London, UK

###### **Correspondence:** A. Sklavounos (alexandros.sklavounos@kcl.ac.uk); D. Scotto (daniele.scotto@kcl.ac.uk)

Background

CT imaging is essential for Hepatocellular Cancer (HCC) diagnosis and staging1. European Association for the Study of the Liver (EASL) guidelines recommend contrast-enhanced triphasic CT imaging for HCC2. Uptake of EASL guidelines has not been uniform across UK NHS trusts, causing delays in HCC diagnosis and treatment.

Aims

To improve the quality of liver CTs and promote uptake of HCC imaging guidelines by Southeast England hospitals. Outcome measures and targets were:

1. 20% reduction in HCC patients undergoing CT rescans
2. 20% reduction in suboptimal CTs
3. 20% increase in triphasic CTs
4. 20% reduction in CTs with inadequate coverage

Methods

We collected data over 22 weeks, analysing 588 patients and 898 CTs from 22 HCC multidisciplinary meetings (MDM). We assessed the quality of liver CTs using EASL guidelines on HCC3. Data from July-September 2018 was classified as baseline. We introduced our interventions between December 2018 and February 2019. We applied Plan-Do-Study-Act (PDSA) quality improvement methodology:

- PDSA 1: Circulation of the EASL/LIRAD algorithm to referring hospitals.
- PDSA 2: Development of an HCC CT-protocol business card
- PDSA 3: Dissemination of CT business cards to referring hospitals.

Results

1. 44.9% reduction in CT rescans
2. 20.6% reduction in suboptimal CTs
3. 55.0% increase in triphasic scans
4. 27.0% reduction in CTs with inadequate coverage.

Discussion

To our knowledge, this is the largest quality improvement study conducted in HCC imaging. If sustained, our improvements will lessen the burden on HCC MDM and radiology departments, minimise delays, improve patient outcomes, and reduce costs associated with rescanning4.

References

1. Ayuso, C., Rimola, J., Vilana, R., Burrel, M., Darnell, A., García-Criado, Á., Bianchi, L., Belmonte, E., Caparroz, C., Barrufet, M. and Bruix, J., 2018. Diagnosis and staging of hepatocellular carcinoma (HCC): current guidelines. European journal of radiology, 101, pp.72-81.

2. Galle, P.R., Forner, A., Llovet, J.M., Mazzaferro, V., Piscaglia, F., Raoul, J.L., Schirmacher, P. and Vilgrain, V., 2018. EASL clinical practice guidelines: management of hepatocellular carcinoma. Journal of hepatology, 69(1), pp.182-236.

3. Tang, A., Bashir, M.R., Corwin, M.T., Cruite, I., Dietrich, C.F., Do, R.K., Ehman, E.C., Fowler, K.J., Hussain, H.K., Jha, R.C. and Karam, A.R., 2017. Evidence supporting LI-RADS major features for CT-and MR imaging–based diagnosis of hepatocellular carcinoma: a systematic review. Radiology, 286(1), pp.29-48.

4. Reid, Scott, 2017. NHS Burden Reduction Plan

### P10 CT and MR imaging of cardiac tumours and pseudomasses

#### E Park, A Prosper

##### University of California Los Angeles, Los Angeles, California, USA

###### **Correspondence:** E Park (eypark@mednet.ucla.edu)

Learning objectives

The purpose of this exhibit is to:

1. Provide a comprehensive review of common cardiac tumours, including their clinical features
2. Present a framework for identifying common cardiac tumours based on location, as well as their computed tomography (CT) and magnetic resonance (MR) imaging features
3. Review tumour mimics, including normal intracardiac variant structures.

Content organisation

Cardiac tumours can have potentially devastating consequences for the patient if not diagnosed and treated appropriately. It is therefore important to be able to identify their imaging characteristics on both CT and MR imaging. Furthermore, being able to differentiate cardiac tumours from tumour mimics such as normal intracardiac variant structures will help avoid inappropriate management.

We will review the epidemiology and imaging features of the following categories of cardiac masses:

- Intracavitary (metastases, myxoma, lipoma, and hemangioma)
- Valvular (papillary fibroelastoma and vegetations)
- Intramural, including malignant (metastases, sarcoma, and lymphoma) and benign lesions (lipoma, paraganglioma, rhabdomyoma, and fibroma)
- Epicardial/pericardial (metastases, hemangioma, lymphangioma, and mesothelioma)
- Tumour mimics and pseudomasses (thrombus, crista terminalis, and a prominent Chiari network)

Conclusion

The range of differential diagnoses for a cardiac mass is wide, spanning the spectrum of benign masses to malignant masses to normal intracardiac variant structures. It is therefore important for the radiologist to be familiar with the imaging characteristics of these cardiac masses and pseudomasses so that they may be appropriately diagnosed and managed.

### P11 Inter-observer repeatability of total bone marrow volume measured from WB-MRI in patients with bone metastases

#### A. Colombo, A. Azzena, P. Summers, F. Zugni, M. Bellomi, G. Petralia

##### Istituto Europeo di Oncologia, Milano, Italia

###### **Correspondence:** A. Colombo (alberto.colombo@ieo.it)

Aim

WB-MRI is an increasingly recommended technique for the evaluation of patients with metastatic bone diseases. Semi-automatic segmentation techniques of diffusion weighted images in WB-MRI examination were developed to allow quantitative evaluation of tumour burden in bone metastases. The aim of this study was to evaluate the inter-observer repeatability of the segmentation of healthy and metastatic bone marrow in patients with bone metastases from breast cancer (BCa) and prostate cancer (PCa).

Materials and Methods

Two independent observers processed eight WB-MRI examinations from patients with bone metastases (4 women with BCa and 4 men with PCa). Segmentation of bone marrow was performed applying a threshold on high b-value diffusion weighted images and manually removing misclassified non-bone regions, and then total volume of bone marrow (Vbm) was obtained. We measured repeatability of Vbm using Dice Similarity Coefficient (DSC) and Intra-class Correlation Coefficients (ICC).

Results

Overall DSC and ICC of Vbm were good and excellent respectively: DSC was 0.80 (95% CI: 0.72-0.88) and ICC was 0.95 (95% CI: 0.78-0.99, p<0.01). We observed higher repeatability in BCa patients, for whom DSC was 0.90 (95% CI: 0.86-0.94) and ICC was 0.99 (95% CI: 0.83-1.00, p<0.01), than in PCa patients, for whom DSC was 0.69 (95% CI: 0.53-0.85) and ICC was 0.91 (95% CI: 0.22-0.99, p<0.05).

Conclusions

Overall DSC and ICC were good and excellent respectively. However, DSC and ICC were higher in BCa patients than in PCa patients.

### P12 The usefulness of Gd-EOB-DTPA late phase in the evaluation of hepatic metastases from GEP-NETs

#### Giannetta V, Funicelli L., Aleksander-Markuszewska Z, Summers P, Fazio N, Bellomi M

##### Postgraduate School in Radiodiagnostics, Università degli Studi di Milano, Milan, Italy - IEO

###### **Correspondence:** Giannetta V (vincenzo.giannetta@unimi.it)

Purpose

To assess the value of hepatobiliary phase (HBP) gadoxetic acid-enhanced magnetic resonance imaging (MRI) as a single-phase protocol in the follow- up evaluation of hepatic metastases from gastroenteropancreatic tumours (GEP-NETs).

Material and methods

We retrospectively reviewed our institution’s medical records of 338 patients diagnosed of liver metastases from GEP-NETs, from 2000 to 2018, identifying 43 patients with pancreatic (n=22) and ileal (n=21) metastasis who underwent at least two Gd-EOB-DTPA-enhanced MR examinations that included 20-minute delayed hepatobiliary phase imaging.

Two radiologists independently evaluated two sets of MRI exam of each patient, baseline and follow-up series, using RECIST 1.1 categorisation and they classified them into one of 3 categories: response, stable and progressive disease.

For the follow up exam, one radiologist reviewed the complete exam, the second-one disposed only of Gd-EOB-DTPA-enhanced 20-min hepatobiliary phase images.

We compared RECIST evaluation of the two readers.

Results

There was a good agreement between the two reviewers (Cohen’s Kappa=0,94) with only 1 discordance in RECIST categorisation (stable vs. progressive disease).

Of the 43 patients included in the study, 5 was excluded because of the incomplete examination. Of the 38 evaluated patients 15/16 were categorised as progressive, 22/21 as stable disease and 1 as a partial response.

Conclusion

In conclusion hepatobiliary phase images obtained after gadoxetic acid–enhanced dynamic MRI have a good diagnostic accuracy in assessment of GEP-NET’s hepatic metastases. Single phase MRI-hepatobiliary phase gadoxetic acid-enhanced MRI should be considered as a short protocol in the interval follow-up in this group of patients.

This abstract has been previously published.

### P13 Unusual metastasis from renal cell carcinoma – Radiological/Pathological correlation and clinical features

#### Murphy G^1^, Goldstein M^1^, Miller B^1^, Wilson P^2^, Zarkar A^3^, Langman G^4^

##### ^1^Departments of Radiology; ^2^Gastroenterology; ^3^Radiation Oncology; ^4^Pathology, University Hospitals Birmingham (Heartlands Hospital), Birmingham, UK

###### **Correspondence:** Murphy G (grainne.murphy@heartofengland.nhs.uk)

Aim

The presentation will discuss an unusual gastric metastasis from renal cell carcinoma, with pathological correlation

Materials and Methods

A patient presented to gastroenterology for upper GI endoscopy which demonstrated a polyp which was biopsied. History was significant for nephrectomy for renal cell carcinoma 8 years prior. CT was performed and dmeonstrated an anterior mediastinal node and an enhancing rounded gastric lesion. Histopathology was in keeping with metastatic renal cell carcinoma.

Results

The presentation will demonstrate the CT imaging findings of renal cell carcinoma metastasis to the stomach.

Teaching points

Gastric metastasis from RCC is rare, occurring in only approximately 0.2% of cases.

The presentation will describe the imaging and pathological features of this unusual entitiy and briefly discuss the more common metastatic locations of renal cell carcinoma.

The patient’s past history is relevant to interpretation, even if remote.

### P14 Markers of response assessment in pediatric patients with bulky Hodgkin lymphoma: PET/MR-based radiomics analyses

#### G Cherobin, G Fichera, R Scotto Opipari, P Zucchetta, D Cecchin, C Giraudo, E Quaia

##### University of Padua, Padua, Italy

###### **Correspondence:** G Cherobin (giuliachero@gmail.com)

Aim

Metabolic assessment is essential for the therapeutic management of pediatric bulky Hodgkin lymphoma (BHL) since lymphatic tissue can be detected on radiological imaging even at full remission. Aim of our study is to assess if MR radiomic features collected by PET/MR are markers of BHL’s response to treatment.

Methods

PET/MRs performed in our tertiary center (2016-2019) for staging and restaging of BHL patients were examined. One nuclear medicine physician measured the FDG uptake (SUVmax and SUVmean) of BHLs, before and after chemotherapy (CHT) and one radiologist used an open source software to perform a 3D segmentation of the mediastinal lymphatic tissue and to extract 33 radiomic features belonging to three categories (first order statistics, gray level co-occurrence matrix, gray level run length matrix). Radiomic features and SUV values before and after CHT were compared (Student’s t-test); the accuracy of the variables showing a statistically significant difference was assessed by receiver operating characteristic curves.

Results

Ten pediatric patients were selected (7 females; mean age 16.75±1.29 yrs). SUVmax (10.78±2.17 vs 2.3±1), SUVmean (7.53±1.6 vs 1.8±0.8) and three out of the 33 investigated radiomic features (energy 119,628,217±111,884,639 vs 20,549,346±22,406,980; correlation 0.37±0.13 vs 0.22±0.25; sum entropy 352,671,364,800±5,4055,822,550 vs 267,849,402,500±109,143,443,700), showed a significant difference, before and after CHT (p<0.05, each). Accuracy was excellent for energy (AUC=0.911), good for sum entropy (AUC=0.800) and poor for correlation (AUC=0.678).

Conclusions

Specific radiomic features could be used as MR markers of response assessment in pediatric BHLs with a strong impact on the diagnostic and therapeutic management.

### P15 CT evaluation of small bowel tumours: a pictorial essay

#### A Zanfardini, P Ramos, F Pomés, M Fernández, A Monjes, M Saborido, E Eyheremendy

##### Hospital Alemán, Buenos Aires, Argentina

###### **Correspondence:** A Zanfardini (azanfardini@hospitalaleman.com)

Learning objectives:

To review the small bowel neoplasms and their imaging features at CT.

Content organisation:

Small bowel neoplasms are infrequent lesions, representing less than 5% of gastrointestinal tumours.

CT is useful for the detection of these lesions. It also provides information about the precise location of the mass, the relationship of the tumour to the lumen, and the presence of concomitant disease.

A small bowel tumour may manifest as an annular lesion, a nodular mass, or an ulcerative lesion.

We will review the imaging features of the benign and malignant tumours of the small bowel:

- Lipomas
- Carcinoid tumours
- Adenocarcinomas
- Gastrointestinal stromal tumours (GIST)
- Lymphomas
- Metastases

We will also discuss the appropriate study protocols for the diagnosis of these entities and the need of another imaging modality (MRI, PET-CT) for a better characterisation.

Conclusions

CT is useful for evaluating small bowel tumours, helps to determine the location of the lesion and the extension of the disease.

The knowledge of the main imaging features of these lesions helps to make to an adequate diagnosis.

### P16 Multiparametric MRI in the presurgical planning of soft tissue tumours

#### M. Nazar, C. Barale, P. Ramos, L. Castro Cavallo, M. Saborido, L. Alvarez, E. Eyheremendy

##### Hospital Alemán, Buenos Aires, Argentina

###### **Correspondence:** M. Nazar (mnazar@hospitalaleman.com)

Learning objectives

Emphasise on the most appropriate and useful MR imaging sequences for assessment of musculoskeletal masses.

Describe MR imaging characteristics that represent typical features of soft-tissue tumours

Analyse the relation between soft tissue tumours and vascular and neural involvement.

Content organisation

Soft-tissue tumours includes a varied group of lesions with different anatomic locations, biologic behavior, and pathologic features. Its radiologic evaluation has been improved drastically by the development of new imaging technology. Currently, multiparametric resonance plays a key role in the analysis of these tumours. The selection and application of the appropriate MRI sequences, can be defining in arriving at the correct diagnosis and therapy approach.

We analyzed patients treated in our institution by our soft tissue tumours team in which MRI multiparametric exams were performed using a 1.5T Phillips Achieva and 3T GE Healthcare Architect using a three-phase dynamic scan. DWI, gadolinium and angiographic time resolution imaging sequences were used.

Conclusions

Lately, multiparametric MRI has become the most useful tool not only to characterise the soft tissue tumours but also to asses vascular and neural involvement which could lead to a different therapeutic approach. Radiologists should be familiarised with this diagnostic method and acknowledge the latest advances in the field.

### P17 Radiomic characterisation of metastatic and localised Wilms tumour

#### G Fichera, L Baffoni, T Toffolutti, M Zuliani, B Giorgi, E Quaia, C Giraudo

##### University of Padua, Italy

###### **Correspondence:** G Fichera (giulia.fichera@studenti.unipd.it)

Aim

Radiomics allow a multifactorial tissue characterisation of neoplastic lesions but, up to now, only a few studies applied it in pediatric imaging. Thus, aim of our study was to investigate the role of CT radiomic features of Wilms tumour for distinguishing between metastatic and localised cancer.

Materials and Methods

Pediatric patients affected by Wilms referring to our tertiary center for staging from 2012 to 2018 and examined by contrast-enhanced CT were included in this retrospective study and then subdivided in two groups (metastatic Wilms (mW) and localised Wilms (lW)). One radiologist with three years experience in oncological imaging drew regions of interest along the margins of all primary tumours, covering the entire volume using an open source software extracting the radiomics features. Three radiomic categories were collected: First Order Statistics, Gray level co-occurrence matrix (GLCM), and Gray-Level Run Length Matrix for a total of 33 examined features. A comparison between mW and lW for each feature has been performed (Student’s t-test;p<0.05).

Results

Overall 14 pediatric patients were included (six mW and eight lW; 8 males; mean age 4.00 ±2.07 yrs). Only six (all GLCM) out of the 33 investigated features showed a statistically significant difference between mW and lW: maximum probability (187,385,487,100±205,565,392,000 vs 427,806,936,000±108,036,120,600), difference entropy (253,333,689,800±352,478,905,300 vs 80,257,092,150±50,281,103,830), inverse variance (171,668,772,800±188,099,095,100 vs 452,336,44960±113,021,712,600), correlation (285,774,657,900±317,191,155,500 vs 111,146,900,900±214,600,007,800), lmc2 (322,955,839,500±358,547,613,600 vs 56,077,741,380±134,156,335,300) and lmc1 (-109,180,806,100±122,928,761,400 vs -1,816,444,640±4,267,472,788) (p<0.05, each).

Conclusion

GLCM radiomic features seem to be biomarkers of metastatic disease in children affected by Wilms.

### P18 Thermal ablation of primary and secondary lung malignancies: a review for diagnostic cancer imagers

#### E Park, K Ruchalski, R Dewan, F Abtin, S Genshaft, R Suh

##### University of California Los Angeles, Los Angeles, California, USA

###### **Correspondence:** E Park (eypark@mednet.ucla.edu)

Learning objectives

The purpose of this exhibit is to:

1. Consider current indications for thoracic thermal ablation and the role of diagnostic imaging in candidate selection
2. Demonstrate the expected computed tomography (CT) and/or FDG-PET imaging features of the immediate post-ablation zone and expected temporal evolution
3. Review unexpected imaging features consistent with tumour progression

Content organisation

Lung cancer is one of the leading causes of death in the world. While surgical resection remains the treatment of choice for patients with early-stage primary lung cancer, approximately 16% of these patients are not surgical candidates due to comorbidities. In such patients, lung-sparing local therapies offer survival benefit. Aggressive local therapies (including thermal ablation) in the multifocal, advanced, oligo-recurrent and oligometastatic lung cancer population may confer survival benefits as well. In those patients with extrathoracic oligometastatic disease to the lung, thermal ablation contributes to improved overall and progression free survival.

We will therefore review the following:

- Indications for thoracic thermal ablation
- Candidates who would benefit from thermal ablation
- Expected evolutionary changes of the post-ablation zone on imaging
- Recurrence detection post ablation

Conclusion

As an alternative to surgical resection and stereotactic radiotherapy, lung-sparing thermal ablation offers the advantages of repeatability, low associated morbidity and mortality, and lower overall medical costs. It confers competitive overall survival in both primary and secondary lung malignancies. Familiarity with this form of local therapy, in particular its imaging findings, is crucial for imaging surveillance and accurate assessment of treatment response.

### P19 Total-body 68Ga-PSMA-11 PET/CT for bone metastasis detection in prostate cancer patients

#### KL Pomykala^1^, M Jardon^1^, J Czernin^2^, J Williams^2^, TR Grogan^3^, J Calais^2^

##### ^1^Departments of Radiology; ^2^Molecular and Medical Pharmacology; ^3^Medicine Statistics Core, David Geffen School of Medicine, University of California Los Angeles, Los Angeles, CA, USA

###### **Correspondence:** J Calais (calais@mednet.ucla.edu)

Aim:

To determine the relationship between serum PSA level and incidence of bone metastases detected by 68Ga-PSMA-11 PET/CT and to assess if expanding the 68Ga-PSMA-11 PET/CT imaging field to include the vertex and lower extremities affects bone metastasis detection and patient management.

Methods:

Retrospective analysis of 388 prostate cancer patients enrolled in five prospective studies (NCT02940262, NCT03368547, NCT03042312, UCLA IRB#17-001336, NCT03515577). All underwent 68Ga-PSMA-11 PET/CT scans acquired from vertex to toes for primary staging (n=93/388, 24%), biochemical recurrence (BCR) localisation (n=225/388, 58%) or re-staging M1 disease (n=70/388, 18%) between September 2017 and May 2018.

Results:

321/388 patients (83%) had a positive 68Ga-PSMA-11 study. PSMA-positive bone lesions were found in 105/388 (27%) patients. Their incidence was positively associated with serum PSA levels (< 10 ng/ml: 21%; 10-20 ng/ml: 41%; ≥ 20 ng/ml: 41%, p<0.001). Bone metastases occurred most frequently in re-staging M1 patients. Bone metastasis incidence was not significantly associated with NCCN risk score (p=0.22). The average number of PSMA positive regions also increased with serum PSA levels (p<0.001). 18/388 (5%) and 18/388 (5%) had lesions above the superior orbital ridge and below the proximal third of the femur, respectively. There was only 1/388 patient (0.25%) in whom the total body PET acquisition had an impact on management.

Conclusion

Including the total body (vertex to toes) for 68Ga-PSMA-11 PET/CT imaging revealed additional bone lesions in 6% of patients, however, without affecting patient management. Bone metastases as assessed with 68Ga-PSMA PET/CT are surprisingly prevalent even in patients with low serum PSA levels.

### P20 Basic pathways of spread of primary tumours, local recurrences and metastatic disease

#### NaHyun Jo, Medhini Rachamallu, Sireesha Yedururi

##### The University of Texas MD Anderson Cancer Center, Houston, Texas, USA

###### **Correspondence:** Sireesha Yedururi (syedururi@mdanderson.org)

Teaching/Discussion Points

- Case based illustrative review of the basic pathways of spread of solid tumours.
- Hematogenous spread
- Lymphatic dissemination
- Intracavitary spill
- Any combination of the above
- Clinical application of the knowledge in accurate staging, predicting future sites of spread and trouble-shooting challenging cases
- Lesions seen in atypical locations and distribution, i.e. not following the basic pathways of spread should be evaluated for the possibility of alternative diagnosis

### P21 IVIM DWI of retropharyngeal lymph nodes: distinguishing benign change from metastases in nasopharyngeal carcinoma

#### Qiyong Ai, Tiffany Y So, Sahrish Qamar, Weitian Chen, Ann D King

##### The Chinese University of Hong Kong, Prince of Wales Hospital, Hong Kong SAR, PRC

###### **Correspondence:** Qiyong Ai (aqy0621@cuhk.edu.hk)

Aim

Size is the main criterion for diagnosis of metastatic retropharyngeal lymph nodes (RPN) from nasopharyngeal carcinoma (NPC) but there is an overlap in size with benign reactive nodes. This study aimed to evaluate if intravoxel incoherent motion (IVIM) DWI can discriminate between malignant and benign RPNs.

Materials and Methods

IVIM DWI using 14 b-values was performed on 38 metastatic RPNs from 28 patients with NPC and 27 benign RPNs from 20 subjects without history of head and neck cancers. The mean, standard deviation (SD), skewness and kurtosis were obtained for the pure diffusion (D), pseudo-diffusion (D*), and perfusion fraction (f) coefficients. Parameters were compared between two groups using the Mann-Whitney U-test. Receiver-operating characteristics analysis was used to identify the optimal threshold and the diagnostic performance was calculated. A p-value of < 0.05 was considered statistically significant.

Results

Compared to benign RPNs, metastatic RPNs showed significantly lower SD for D (0.18 ± 0.06 vs 0.13 ± 0.03 × 10-3mm2/s) and f (0.10 vs 0.08) (all p < 0.05), but not for D* (p = 0.699). Differences of the mean, skewness and kurtosis for all IVIM parameters between two groups were not significant (all p > 0.05). DSD provided the highest area under the curve of 0.74 with the optimal threshold of < 0.15 × 10-3mm2/s showing a sensitivity of 78.9%, specificity of 70.4%, and an accuracy of 75.8%.

Conclusion

IVIM DWI has potential to discriminate NPC metastatic RPNs from benign ones and DSD was the most promising parameter.

### P22 Breast incidentalomas: differentiating benign from malignant breast lesions on computed tomography

#### I Desai^1^, F Urdaneta^2^, S Chen^3^

##### ^1^University of California Los Angeles, Los Angeles, California, USA; ^2^University of Southern California, Los Angeles, California, USA; ^3^Cedars Sinai Medical Center, Los Angeles, California, USA

###### **Correspondence:** I Desai (idesai@mednet.ucla.edu)

Learning objectives

To help the general radiologist and dedicated non-breast oncologic imager identify what constitutes normal breast appearance on computed tomography (CT) of the chest.

To help better categorise and describe benign and malignant breast findings.

Content organisation

Given the sheer number of CTs performed today, in the emergency department and now for lung cancer screening, there is increased importance of being able to identify these findings on non-breast imaging. In some cases, a chest CT may be the baseline scan for breast lesions and may even prove to be the first detection of primary breast malignancy.

We will discuss the following:

- The appearance of the normal breast on CT.
- Common CT characteristics of benign breast lesions.
- Common CT characteristics of malignant breast lesions.
- Review of current literature for the analysis of incidental breast findings on CT.

Conclusions

Because of the rapid rise in the number of chest CTs, it is increasingly important to be able to recognise concerning breast findings on chest CT. The common characteristics and discussion of current data for these findings should help radiologists better aid the clinician and breast radiologist in further work up.

### P23 Identifying common and uncommon imaging manifestations of chemotherapy-induced complications in the gastrointestinal system

#### I Desai^1^, F Urdaneta^2^

##### ^1^University of California Los Angeles, Los Angeles, California, USA; ^2^University of Southern California, Los Angeles, California, USA

###### **Correspondence:** I Desai (idesai@mednet.ucla.edu)

Learning objectives

To be able to identify uncommon and common gastrointestinal complications related to chemotherapy. To be able to differentiate benign post-treatment imaging appearance from actionable complications.

Content organisation

With the increasing use chemotherapeutic agents and directed cancer treatments, the radiologist can play an increasingly important role in not only identifying imaging manifestations of post-treatment complications but also in appropriately attributing the findings to the causative agent.

- Discussion of common agents and treatments associated with gastrointestinal toxicity and the risk factors associated with the development of complications.

-Pictorial review of gastrointestinal toxicities, highlighting actionable complications where applicable.

o Discussion of chemotherapy-related imaging findings of but not limited to: gastritis, enteritis, colitis, pneumatosis, perforation and megacolon.

Conclusions

The awareness of complications of common cancer therapies is vital to the radiologist in that it can significantly aid in increased detection and attribution of these radiologic manifestations to the appropriate culprit.

### P24 Prostate Specific Membrane Antigen (PSMA) and Fluoro-deoxy glucose (FDG) Positron Emission Tomography in Prostate Cancer

#### CNB Harisankar (hari.cnb@gmail.com)

##### Department of Nuclear medicine, Meenakshi Mission Hospital and Research Centre, Madurai, Tamil Nadu India

Learning objectives

To review the patterns of imaging findings in prostate cancer using PSMA and FDG PET-CT scans. The role of PSMA PET scan in evaluation of prostatomegaly before prostatic biopsy, staging of newly diagnosed prostate cancer, biochemical recurrence following prostatectomy and in follow up of metastatic prostate cancer will be highlighted. Role of FDG PET in hormone refractory prostate cancer will be elucidated.

Content organisation

Prostate cancer diagnosis and evaluation of extent of disease is expected to witness a major change after availability of PSMA PET scanning technique. PSMA has been shown to be highly sensitive in identification of prostate cancer and possibly outperforms other conventional imaging modalities.

FDG PET has lower sensitivity in prostate cancer in view of low glycolytic activity of these tumours. However, FDG PET is useful in patients with hormone refractory prostate cancer.

The different scenarios of PSMA PET presented will be as follows:

1. PSMA PET scan - Physiologic tracer distribution
2. PSMA PET Scan - Before biopsy in a patient with and without prostatomegaly and increased serum PSA levels
3. PSMA PET Scan - In a biopsy proven prostate cancer - patterns of metastatic disease
4. FDG PET scan - Different patterns of positive findings and incidentally noted prostate cancer

Conclusion

Prostate specific membrane antigen PET imaging is a highly sensitive and specific imaging in prostate cancer. PSMA PET has potential to be a one-stop imaging modality in prostate cancer. PSMA PET may potentially identify candidates for whom biopsy may be avoided.

### P25 Sub-staging metastatic disease from a diverse spectrum of non-neural solid tumours: Cancer imagers’ perspective

#### Sireesha Yedururi^1^, Leonardo Marcal^1^, NaHyun Jo^1^, Venkata Subbiah Katabathina^2^, Medhini Rachamallu^1^, Srinivasa Prasad^1^

##### ^1^The University of Texas MD Anderson Cancer Center, Houston, Texas, USA; ^2^UT Health San Antonio, San Antonio, Texas, USA

###### **Correspondence:** Sireesha Yedururi (syedururi@mdanderson.org)

Learning Objectives

To discuss and illustrate the link between anatomic and physiologic connections and spread of tumour (with emphasis on venolymphatic drainage and lymphaticovenous connections)

Why and how metastatic disease in different locations from the same primary tumour may imply similar/different prognoses

How metastatic disease is a continuous and ongoing process and how metastases further metastasise.

Content Organisation

Introduction to metastatic disease.

Brief review of existing sub classification of metastatic disease for some cancers (e.g. lung cancer and colorectal carcinoma) and their pitfalls.

Rationale for the need to sub-classify metastatic disease in the age of immunotherapy and targeted chemotherapy.

Role of venous drainage, lymphatic drainage and lymphaticovenous connections in the spread of tumours.

New proposal for sub-staging of metastatic disease that can be applied to a diverse spectrum of non-neural solid tumours, regardless of location or tumour sub-type

Practical applications of the newly proposed sub-staging of metastatic disease in routine oncologic imaging practice.

Conclusion:

Not all metastatic disease implies terminal illness nor do metastatic disease at different locations from a primary tumour carry similar prognosis. The existing staging systems for most cancer do not go beyond the initial staging. However, with improving survival of patients with metastatic disease, further classification of metastatic disease is helpful in understanding the stage and prognosis of disease. We propose and illustrate a new sub-staging scheme for metastatic disease for a diverse spectrum of non-neural solid tumours based on the knowledge of pathways of spread of tumours into M1, M2 and M3.

### P26 Machine learning and artificial intelligence in oncologic imaging: Potential barriers and solutions, abdominal imagers’ perspective

#### Sireesha Yedururi^1^, Venkata Subbiah Katabathina^2^, NaHyun Jo^1^, Medhini Rachamallu^1^, Srinivasa Prasad^1^, Leonardo Marcal^1^

##### ^1^The University of Texas MD Anderson Cancer Center, Houston, Texas, USA; ^2^UT Health San Antonio, San Antonio, Texas, USA

###### **Correspondence:** Sireesha Yedururi (syedururi@mdanderson.org)

Learning Objectives

Learn how the following day to day practices could potentially hinder machine learning

1. heterogeneity in the language used by radiologist to describe all sites of disease beyond the PIRADS, LIRADS etc., for primary tumours
2. lack of standardised annotations of all sites of tumour
3. lack of standard nomenclature for lymphatic dissemination (e.g. regional nodal disease is described as lymphatic metastasis in routine practice and literature)

Understand the scenarios in which the existing machine learning algorithms are efficient (presence or absence of suspicious lung nodules or breast lesions).

Understand the challenges of machine learning in other scenarios (e.g., less inherent soft tissue contrast in the abdominal viscera, unclear or incorrect clinical question, co-existence of findings unrelated to the malignancy and/or complications of treatment)

Content Organisation

Introduction

Discuss the existing machine learning and artificial intelligence algorithms

Usefulness and limitations of existing algorithms

Review the potential barriers to machine learning in oncological imaging

Discuss potential solutions to facilitate machine learning e.g.

1. standardised descriptions for all sites of disease on baseline and follow up imaging (benign, indeterminate but likely benign, indeterminate, indeterminate but likely malignant and malignant)
2. standard annotations

Review some persistent hurdles

Conclusion

Machine learning has shown promising results in lung nodule and breast lesion detection. However, there are multiple potential barriers to machine learning in routine oncologic imaging, particularly in the abdomen. We will summarise oncologic imagers’ perspective of potential barriers to machine learning in oncologic imaging and list some potential solution.

### P27 Imaging findings of mediastinal and chest wall toxicity after streotactic body radiotherapy

#### KJ Park, SG You, JS Sun, OK Noh

##### Ajou University, Suwon, South Korea

###### **Correspondence:** KJ Park (kjpark@ajou.ac.kr)

Learning objectives:

To review the imaging findings of radiation effect and complication in the mediastinum and chest wall after streotactic body radiotherapy (SBRT) of lung cancer.

Content organisation:

SBRT is increasingly used for the treatment of non-small cell lung cancer in medically inoperable patients. In addition to the postradiation lung injuries that are known to be different in the extent and pattern from those after conventional radiation therapy, SBRT has a higher risk for mediastinal and chest wall complications due to highly concentrated irradiation than conventional radiotherapy.

We will review pathophysiology, clinical manifestations, and imaging findings of postradiation change and toxic effect of SBRT to the mediastinum and chest wall, and discuss possible differential diagnosis.

- Airway – wall thickening, narrowing, atelectasis, necrosis and fistula
- Esophagus – esophagitis, stricture, perforation, tracheobronchial fistula
- Cardiac toxicity – change in pericardium and myocardium
- Pulmonary artery and aorta- vasculitis, aneurysm, hemoptysis
- Chest wall toxicity – skin change, chest wall edema, rib fracture
- Others – pneumothorax, vagus nerve or brachial plexus injury

Conclusion:

In lung cancer patients undergoing SBRT, there can be various complications in the mediastinum and chest wall with diverse imaging findings. Knowledge of the imaging findings of such complications will be helpful for accurate diagnosis and rapid treatment.

### P28 Diffusion weighted MRI for the differentiation between xanthogranulomatous cholecystitis from malignancy

#### S Mohamed^1,2^, I Sulieman^3^, A Elaffandi^3,4^, W Elmoghazy^3,5^, H Khalaf^3^

##### ^1^Department of Clinical Imaging, Hamad Medical Corporation, Doha, Qatar; ^2^Department of Clinical Imaging, National Cancer Institute, Cairo University, Egypt; ^3^Department of Surgery, Organ Transplant & Liver Unit, Hamad General Hospital, Doha, Qatar; ^4^Department of Surgical Oncology, National Cancer Institute, Cairo University, Egypt; ^5^Department of Surgery, Sohag University, Sohag, Egypt

###### **Correspondence:** A Elaffandi (saffandy2000@yahoo.co.uk)

Introduction

Xanthogranulomatous cholecystitis (XGC) is a rare variant of chronic cholecystitis that closely mimics gallbladder cancer (GBC) and is a challenge to differentiate it preoperatively. Differentiation is important because it dictates the extent of surgical resection needed for cure. MRI is currently the gold standard for diagnosing gallbladder malignancy. We are examining the value of diffusion weighted images in the differentiation of XGC from GBC.

Methods

All of the patients presenting to Hamad Medical Corporation from January 2011 to January 2016 with histopathologically proven diagnosis of GBC or XGC and available MRI with DWI were included in the study. The b800 and b0 values were calculated from the DWI sequences and compared.

Results

During the study period 6 patients were diagnosed with histopathology with XGC and 14 patients with GBC and had DWI MRI available. The mean b800/b0 was 0.43 (±0.1) for the XGC, and 0.48 (±0.4) for the GBC cases. There is an observational difference between the two groups that is limited by the sample size; however, such difference was not significant (Wilcoxon Rank sum test: p= 0.62).

Conclusion

XGC is difficult to differentiate from GBC on imaging due to the locally infiltrative behavior of this inflammatory lesion. Tissue diagnosis remains of high value in setting the difference, however limited by its negative predictive values preoperatively. The use of DWI and the b800/b0 ratio as a new parameter showed an observational difference with high values in GBC that perhaps may be significant in larger sample size.

### P29 The value of diffusion weighted imaging in diagnosing gallbladder malignancy: The performance of new parameter

#### S Mohamed^1,2^, I Sulieman^3^, A Elaffandi^3,4^, W Elmoghazy^3,5^, H Khalaf^3^

##### ^1^Department of Clinical Imaging, Hamad Medical Corporation, Doha, Qatar; ^2^Department of Clinical Imaging, National Cancer Institute, Cairo University, Egypt; ^3^Department of Surgery, Organ Transplant & Liver Unit, Hamad General Hospital, Doha, Qatar; ^4^Department of Surgical Oncology, National Cancer Institute, Cairo University, Egypt; ^5^Department of Surgery, Sohag University, Sohag, Egypt

###### **Correspondence:** A Elaffandi (saffandy2000@yahoo.co.uk)

Introduction

Diffusion weighted imaging (DWI) is a relatively recent technique in MRI imaging that examines the freedom vs restriction of motion of water molecules in tissues and helps to diagnose malignancy. In this study, we aim to study the value of b800/b0 in addition to ADC values.

Methods

Patients presenting with suspicious gallbladder lesions to Hamad Medical Corporation between January 2011 and December 2016 were identified. Those with MRI with DWI sequences and histopathologic diagnosis of the Gallbladder lesions were included.

Results

32 patients were identified, including: 24 (75%) Males and 8 (25%) females. This included 18 cases with benign liver lesions (Chronic cholecystitis: 9; Acute cholecystitis: 1; Xanthogranulomatous cholecystitis: 4; Adenomas: 3; Low grade dysplasia: 1), and 14 malignant (Adenocarcinoma: 12; NET: 1; Adeno-squamous carcinoma: 1). The mean ADC value for the malignant cases was 1.62 (±0.57) x 10-3 mm2/s and the benign cases was 1.27 (±0.39) 10-3 mm2/s. The difference was not significant (p=0.0773). The mean b800/b0 ratio for benign cases was 0.31 (+-0.19) and for malignant cases was 0.48 (+-0.13) with a significant difference (p=0.007). The ROC curve for the b800/b0 had an AUC of 0.782 (95% CI: 0.616 – 0.947). At a cutoff point of 0.33, the sensitivity is 85.7% and the specificity is 72.2%.

Conclusion

The b800/b0 ratio in diffusion weighted imaging could help to differentiate benign from malignant gallbladder lesions, and it may be more reliable than the ADC values in the quantitative assessment of the DWI.

### P30 Relation between risk of malignancy and location in thyroide nodules: Our experience and outcomes

#### P. Sidelski, M. Grana, N. Florenzano, L. Tisser, A. Monjes, M. Fernandez, E. Eyheramendy

##### **Correspondence:** A. Monjes (alejandramonjes@hotmail.com)

Aim

To analise the distribution of malignant thyroid nodules detected by sonography routine tests, focusing in their anatomical location.

To compare the results with recent publications that suggest a higher frequency of malignancy in upper pole nodules.

Methods

We performed a retrospective study on the ultrasound characteristics of thyroid nodules from 123 patients from June 2010 to June 2016. We analyzed polarity of the biopsied nodules with confirmed malignancy: upper pole versus middle pole versus lower pole lobe location, depending on the region that contains more than 50% of it

Results: In total, 56% (n=69) of all thyroid nodules were found to be malignant. There were 19 males and 50 females, aged between 11 and 74 year old.

Malignant nodules were more frequent in the lobes (95%) than in the isthmus (5%) but no significant difference between the two lobes.

We found a higher prevalence in the middle pole (41%), followed by the lower pole (29%) and isthmus (4%).

Conclusions

Some nodule characteristics have a well-established association with malignancy. However, there is less information regarding the association between the likelihood of malignancy and the location of the nodules.

In our study, analysis of the distribution of malignant thyroid nodules showed a higher prevalence of lesions in the inferior pole, with no differences between the two lobes. Furthermore, prospective studies are needed to confirm these results and demonstrate an association between thyroid nodule location and the likelihood of thyroid nodule malignancy.

### P31 Day-case radiologically inserted gastrostomy (RIG): a safe and viable option

#### D Kotecha, Neel Raja, Michael Adeleye, B Billimoria

##### Kettering General Hospitals NHSFT, Kettering, United Kingdom

###### **Correspondence:** D Kotecha (deevia.kotecha@gmail.com)

Background

In 2018 our institution started a service for insertion of direct percutaneous radiologically inserted gastrostomy (RIG) as a day case procedure. Patients are admitted from home to our Radiology Day Case unit. A RIG insertion is performed under sedation and the patients are discharged home to Community Enteral Nutrition Nurse Support with dietetic input 4 hours post procedure.

Aims

To evaluate the efficacy and safety of RIG insertion as a day case procedure.

Methods

Data was collected retrospectively for radiologically inserted gastrostomies performed between June 2018 to March 2019. Information was gathered utilising procedure notes and any available subsequent admission discharge summaries.

Results

A total of 15 radiologically inserted gastrostomy procedures were performed. Technical success was 100%. No immediate complications (<24 hours post-procedure) occurred post-RIG insertion. All patients were discharged 4 hours after the procedure. No patients were re-admitted with complications within 72 hours post procedure.

3/15 (20%) patients developed complications within 30 days post-procedure. Minor complications included: tube displacement (n=1), leakage around the tube (n=1). A replacement tube was required in one patient (6.6%). The 30-day all-cause mortality rate was 13.3% (n=2). There were no gastrostomy-related deaths.

Conclusion

Our early experiences show that day case radiologically inserted gastrostomy procedures and can be safely performed as a day case procedure with careful case selection and close patient follow up. Procedures were associated with a low complication rate and were economically advantageous.

### P32 Pseudolesions in fatty liver of oncologic patients. MR utility

#### S. De Luca, M. Nazar, E. Casalini Vañek, P. Ramos, F. Pomés, G. Rodriguez, E. Eyheremendy

##### Hospital Alemán, Buenos Aires, Argentina

###### **Correspondence:** S. De Luca (sdeluca@hospitalaleman.com)

Teaching points

To emphasise on the value of MR specially, in phase and out of phase, for lesions assessment and in fatty liver.

To describe imaging findings suggestive of pseudolesions in fatty liver and rule out true masses.

To demonstrate the importance of an adequate oncological follow-up algorithm in patients with fatty liver with special focus in oncologic patients who underwent chemotherapy treatments.

Contents organisation

Fatty liver is characterised histologically by triglyceride accumulation within the hepatocytes.

Particularly in oncologic patients is a very common scenario after chemotherapy treatments.

MR evaluation plays a key role in the analysis of these patients. The selection and application of the appropriate MRI protocol, can define the correct diagnosis avoiding invasive diagnostic methods.

We consider that these points are the most useful to characterise the fatty liver lesions and pseudolesions are: determining fat content with adequate sequences (out and in phase), location in characteristic areas of fat deposition or sparing, absence of a mass effect on vessels and other liver structures, geographic configuration, poorly delineated margins and similar enhancement to healthy parenchyma.

Misinterpretation of this normal areas can lead to invasive studies and unnecessary biopsies, modifying the appropriate management in these patients.

Conclusion

In patients with cancer, it is common to find post-chemotherapy fatty liver, in these cases the use of MRI is the indicated method since it is more sensitive in the detection of focal lesions.

### P33 Pitfalls and limitations in lung cancer staging

#### C. Carrera, S. De Luca, C. Barale, F. Pomés, M. Escolar, C. Benitez, A. Ramirez, E. Eyheremendy

##### Hospital Alemán, Buenos Aires, Argentina

###### **Correspondence:** C. Carrera (ccarrera@hospitalaleman.com)

Teaching points

- Review possible errors in the lung cancer staging images.
- Mention the clinical implications related to these errors.

Content organisation

Lung cancer is one of the leading oncologic cause of mortality worldwide. Clinical practice guidelines for lung cancer largely rely on staging models, which are used not only for predicting disease prognosis, but also to guide treatment. The 8th Tumour, Node, Metastasis (TNM) staging system derived from validation of the TNM system for guiding lung cancer treatment in multidisciplinary centers.

Making mistakes in a correct staging by the TNM can lead to unnecessary procedures and treatments or underestimate the disease. For this reason, it is important to take into account which are the most frequent errors to avoid erroneous diagnoses.

T: Special care must be taken with the correct measurement of tumour size, taking into account the presence of spicules that do not oversize the actual size of the tumour, since this leads to changes in the management of surgery or surgery and chemotherapy.

N: Take into account that size is not a reliable parameter. There may be false positives (due to inflammation) or false negatives (microscopic tumour).

M: have a special care with false negatives that can be metastasis with low metabolic activity or false positives (fractures).

Conclusion

Review the frequent errors of the classification by images of the TNM, which allows to improve the perfomance of the staging and treatment.

### P34 Baseline quantitative texture features predict tumour PD-L1 status in advanced non-small cell lung cancer

#### Kathleen Ruchalski^1^, Grace Hyun Kim^1^, Antonio Gutierrez^1^, Andrew Tucker^2^, Aaron Lisberg^2^, Joshua Lai^1^, Matthew Brown^1^, Jonathan Goldin^1^, Jonathan Goldman^2^, Denise R Aberle^1^ and Edward Garon^2^

##### ^1^UCLA Department of Radiological Sciences, Los Angeles, CA USA; ^2^UCLA Department of Medicine, Division of Hematology/Oncology

###### **Correspondence:** Kathleen Ruchalski (kruchalski@mednet.ucla.edu)

Aim

Tumour programmed death receptor-ligand 1 (PD-L1) status contributes to treatment selection and prognosis in patients with advanced non-small cell lung cancer (NSCLC). Our aim is to create a multivariate logistic model using baseline CT texture features that can predict tumour PD-L1 positivity.

Materials and Methods

This retrospective analysis included 97 patients with NSCLC from our institution enrolled in the KEYNOTE-001 clinical trial of pembrolizumab from May 2012-September 2014. Patient characteristics and tumour PD-L1 binary score (PD-L1 positive, ≥1% membranous staining) were obtained. Target lesions (TL) were identified on baseline CT of the chest, abdomen and pelvis and TL volumes were manually contoured on a quantitative imaging workstation. Of 262 texture features extracted 22 were selected using the backward feature selection with Akaike information criteria.

Results

Of the 97 patients reviewed, 27 patients were excluded due to missing data or no identifiable TL by irRC. Cohort characteristics included: mean age of 63, 52% male & 54% current/ever smokers. PD-L1 status was positive in 74 patients, negative in 11 patients, and unknown in 12 patients. There were 225 TLs in the following locations: 103 lung, 56 lymph node, 28 liver, 12 adrenal, 20 other. Our texture feature model correctly classified PD-L1 status 88.2% of the time (AUC=0.92; sensitivity 89%; specificity 82%; PPV 96%; NPV 59%).

Conclusions

A multivariate logistic model using baseline CT texture features can predict tumour PD-L1 status. While these findings require further validation, this technique may provide a noninvasive approach in guiding treatment selection and patient prognosis.

### P35 PET/CT in lung cancer staging: limitations and pitfalls

#### Meghan Jardon, Kelsey L Pomykala, Rohit Dewan, Antonio Gutierrez, Martin Auerbach, Pawan Gupta, Kathleen Ruchalski

##### University of California, Los Angeles. Los Angeles, California, USA

###### **Correspondence:** Meghan Jardon (mjardon@mednet.ucla.edu)

Learning Objectives

The purpose of the exhibit is to:

- Discuss the diagnostic utility and indications of PET/CT in the initial evaluation and staging of non-small cell lung cancer (NSCLC)
- Provide a pictorial review of limitations and possible pitfalls of PET/CT in the current TNM (primary tumour, lymph node, metastases) staging guidelines for NSCLC

Content Organisation

- A brief review of the clinical indications of PET/CT for the initial diagnosis and evaluation of NSCLC
- A discussion of possible pitfalls of the use of PET/CT in initial diagnosis and staging of NSCLC primary lung tumours: including small size, sub-solid lesions and mucinous adenocarcinoma histology
- An evidence based review of the benefits and limitations of PET/CT in assessing for pathologic regional lymph nodes
- A discussion of the few limitations of PET/CT in evaluation for distant metastatic disease, including occasional adrenal and brain metastases

Conclusion

PET/CT is a valuable tool in the diagnosis and staging of NSCLC. However, there are possible pitfalls when assigning a TNM stage that the oncologic imager should consider. By being familiar with these limitations and considering alternate clinical explanations for these PET/CT findings, oncologic imagers can provide more accurate staging classification.

### P36 Abdominal toxicities from Immune Checkpoint Inhibitors: What the radiologist needs to know

#### M Braschi-Amirfarzan, R Thomas, F Fennessy

##### Beth Israel Lahey Health, Brigham and Women’s Hospital, Dana Farber Cancer Institute, Boston, USA

###### **Correspondence:** M Braschi-Amirfarzan (martabraschi@yahoo.it)

Learning Objectives:

After viewing this exhibit, the participant will be able to identify and correctly interpret abdominal immune-related adverse events (irAE) from immune checkpoint inhibitors therapy, including anti CTLA-4, PD-1 and PD-L1 inhibitors and understand their clinical implications.

Content Organisation:

In the last 10 years checkpoint inhibitors have changed the way oncologists approach and treat most of the solid and hematological malignancies. Hence, it is important for the radiologist to keep abreast of the irAEs. A comprehensive review of the abdominal immune checkpoint inhibitors’ toxicities will be presented in a case base format, including:

- colitis
- hepatitis
- pancreatitis
- cystitis
- myositis

Conclusion:

Since check point inhibitors are increasingly used for cancer treatment, it is important for the radiologist to promptly identify irAE to guide the clinician in the best next step in management.

### P37 Challenges in applying RECIST 1.1 in cholangiocarcinoma

#### Meghan Jardon, Kelsey L Pomykala, Michael Douek, Jonathan Goldin, Kathleen Ruchalski

##### University of California, Los Angeles. Los Angeles, California, USA

###### **Correspondence:** Meghan Jardon (mjardon@mednet.ucla.edu)

Learning Objectives

The purpose of the exhibit is to:

- Highlight the challenges of defining Measurable Disease, per RECIST 1.1 guidelines, required for clinical trial eligibility determination
- Identify the site and nature of lesions resulting in the determinations of Progression Free Survival per RECIST 1.1 guidelines
- Provide guidelines for optimizing RECIST 1.1 in the setting of cholangiocarcinoma including: accuracy and reproducibility of size assessment in infiltrative tumour as well as alterations in tumour appearance in relation to phase of intravenous contrast

Content Organisation

- A brief review of RECIST 1.1, including guidelines in baseline lesion selection for objective response and post treatment categorical tumour response evaluation
- A pictorial description of the challenges observed when evaluating treatment response of cholangiocarcinoma with RECIST 1.1
- A case based review of typical and atypical patterns of extrahepatic metastatic disease in cholangiocarcinoma

Conclusion

RECIST 1.1 has been widely adopted as a quantitative measure of response assessment of solid tumours, both in standard treatment and clinical trials. Due to the infiltrative nature of the disease and unique imaging characteristics, cholangiocarcinoma can be a challenging disease for the application of RECIST 1.1 criteria. When assessing response to therapy, oncologic imagers should be aware of these cholangiocarcinoma-specific pitfalls to ensure an accurate assessment of a patient’s disease burden.

### P38 Tunnelled Central Venous Lines: A review of our practice

#### M Adeleye, N Raja, D Kotecha, B Bilimoria, W Tan

##### Kettering General Hospital NHSFT, Kettering, United Kingdom

###### **Correspondence:** M Adeleye (ma813@leicester.ac.uk)

Aims

To evaluate the complication rates for tunnelled central venous catheters that were inserted for the use of chemotherapy in our service delivered by a Consultant Interventional Radiologists from 2016-2017.

Method

A retrospective study of patients who had tunnelled central venous catheters (TCVC), for chemotherapy, inserted between 2016 and 2017 was performed. The events and outcomes were determined by reviewing all electronic patient results and pathways.

Results

69 consecutive patients (mean age 59.5; 24 - 80 years) underwent a TCVC placement by a Consultant Interventional Radiologist, for chemotherapy administration, in our institution from January 2016 to December 2017.

For 6/69 (8.7%) the outcomes could not be determined, and these patients were excluded.

In the remaining cohort of 63 patients, there were 70 insertions of TCVC (27 Hickman and 43 Groshong lines).

The lines had a mean duration of insertion of 156 days.

The complication rate was 14.3% - 10/70 lines being removed due to confirmed complications. The reasons for removal were line displacement (n=3), infections (n=6) and bleeding at the line site (n=1).

The patients were followed up until January 2019, with a maximum follow up time of 3 years and a minimum of 13 months.

Conclusions

Our findings show that our service of Consultant Interventional Radiologists, as the primary operator for TCVC insertion is a safe service with a low risk of complication (14.3%).

### P39 Total metabolic volume value in PET/CT non-measurable disease

#### S. De Luca, C. Carrera, E. Casalini Vañek, C. Benitez, M. Fernandez, F. Pomés, E. Eyheremendy

##### Hospital Alemán, Buenos Aires, Argentina

###### **Correspondence:** S. De Luca (sdeluca@hospitalaleman.com)

Learning objectives

Consider the importance of metabolic disease beyond the morphological response in patients under cancer treatment.

Know the importance of metabolism in PET / CT non-measurable disease.

Describe our experience in the daily practice of RECIST (Response Evaluation Criteria In Solid Tumours) and PERCIST (PET response criteria in solid tumours) criteria.

Content organisation

The RECIST and PERCIST criteria are standard methods to evaluate the response to treatment in oncologic patients. In this way, it is possible to determine the existence of four types of possible stage in the evolution of the disease: complete response (CR), partial response (PR), progressive disease (PD) and stable disease (SD). However, it should be mentioned that changes induced by treatments, modify the biology and tumour behavior, and this could cause a discordance between metabolic and morphologic changes.

The interpretation of the therapeutic response in a morphologically non-measurable hypermetabolic lesion is sometimes conflictive, such as in bone lesions, ascites, pleural effusion, lymphangitis carcinomatous, diffuse peritoneal carcinomatosis, etc.

Likewise there are hypermetabolic lesions that modify their metabolic activity before their morphological changes are evidenced.

We propose the possibility of using the variation of the Total Metabolic Volume (TMV), independently of the morphological changes as a predictor of early response to treatment in non-measurable disease.

Conclusions

The TMV takes on a relevant role if the lesion is not measurable and hypermetabolic, constituting a fundamental tool in oncological monitoring in daily practice.

### P40 MR assessment of clinically significant prostate cancer: current standards and future directions

#### F. Fennessy (ffennessy@bwh.harvard.edu)

##### Dana Farber Cancer Institute, Brigham Health, Boston, USA

Prostate cancer is a heterogenous disease with varied biological aggressiveness. The aim of this case-based presentation is to explain the role of qualitative and quantitative multiparametric MRI (mpMRI) assessment in the detection of clinically significant prostate cancer, and to present the current limitations of mpMRI in assessing clinically significant disease (with respect to the final pathology at prostatectomy). Through histopathological correlation, this presentation will demonstrate where mpMRI does well in tumour detection (e.g. in detection of dense tumours) and where mpMRI does not perform well (e.g. in detection of sparse tumour and small tumours) and will explain why this is so. We will discuss the benefits of qualitative (PIRADS v2) vs. quantitative mpMRI (quantitative diffusion and dynamic contrast imaging) in assessment of prostate cancer. We will also review novel approaches (e.g. multidimensional diffusion MRI) being investigated for overcoming current limitations in standard mpMRI of the prostate for detection of clinically relevant disease.

### P41 Thirteen years of Intraductal Papillary Mucinous Neoplasm (IPMN) follow-up: a preliminary study

#### F. Maio^1^, V. Pasqualino^2^, S. Rossi^2^, V. Cantoni^1^, G. Morana^3^

##### ^1^Federico II University Naples Italy; ^2^Padua University Padua Italy; ^3^Ca’ Foncello Hospital Treviso Italy

###### **Correspondence:** F. Maio (francescamaio9@gmail.com)

Aim

Intraductal papillary mucinous neoplasm (IPMN) of the pancreas is cystic lesion, with potential ability to evolve. Lifelong follow-up is recommended in patients who are fit for surgery. This single-center study analysed thirteen years of IPMN follow-up.

Materials and methods

733 pz with a known IPMN lesion (>3mm) and at least two abdominal MRI were selected from our radiological database. Two readers compared the first and the last MRI exam for each patient. They indicated type of IPMN (BD/MD/mixed), localisation, number and size of lesions. The mean follow-up time was 49,2±38,4 months. Univariable and multivariable linear regression analyses were performed to examine the relationship between the size value of the lesion at last control with age, sex of patient, type, number and localisation of lesions and their size at first control. We considered for the multivariable analysis only variables statistically significant at univariable analysis.

Results

}At univariate regression analysis considering as dependent variable IPMN size values, the age of patient, the uncinate process lesion localisation, the lesion size at the first control, the mixed IPMN type and multiple lesions are directly related (p<0.05) with the growth of lesion. At the multivariable analysis the lesion size value at first control is associated with the lesion size values at last control. Furthermore, the length of follow-up showed a positive correlation with the lesion size value at last control only for lesions <10mm.

Conclusions

Our results show an important role of the IPMN size at first control, especially for lesions <10mm whose follow-up is recommended.

### P42 Sonographic differentiation between benign and malignant ovarian cysts

#### Shoubhi Bhatnagar (shoubhimd@gmail.com)

##### QEQM Hospital, EKHUFT, London, UK

Learning objectives

To review spectrum of imaging features of benign and malignant ovarian cysts.

Content organisation

Ovarian cysts are routinely detected on sonography. Many times these cysts are functional and need no further evaluation. In such cases, repeat ultrasound usually proves the diagnosis.

Identifying key sonography findings that suggest possible malignancy is essential for not only early detection of cancer, but also prevents unnecessary investigations in benign lesions thus reducing workload in already burdened imaging department.

I will review various features that suggest functional/ benign or malignant nature of ovarian cysts.

Conclusion

A basic approach to sonographic characterisation of ovarian cysts is necessary for both sonologists and radiologists to ensure timely detection of potential cancer and preventing further investigation of benign lesions.

### P43 Peritoneal spread in gynaecological malignancies

#### Shoubhi Bhatnagar (shoubhimd@gmail.com)

##### QEQM Hospital, EKHUFT, London, UK

Learning objective

To review various patterns of peritoneal spread in gynaecological cancer.

Content organisation

CT evaluation in cases of suspected or known gynaecological malignancies is commonly undertaken for detection of metastatic spread of disease. Extensive peritoneal involvement is often found in advanced cases. This becomes key in making decision regarding patient treatment.

The sites of peritoneal involvement can be examined systematically, avoiding incorrect diagnosis in cases of localised or limited spread of disease.

I will review various patterns of peritoneal involvement and discuss in brief about standard approach in these cases.

Conclusion

Metastatic peritoneal spread of gynaecological malignancies is a common occurrence that effects patient treatment. A step by step review of peritoneal sites, prone to metastases, helps in quick and efficient diagnosis.

### P44 MRI evaluation in endometriosis

#### Shoubhi Bhatnagar (shoubhimd@gmail.com)

##### QEQM Hospital, EKHUFT, London, UK

Learning objective

To review MRI findings in cases of endometriosis.

Content organisation

Endometriosis is a known cause of intractable abdominal pain and infertility with variable presentation in young females. The diagnosis is often suggested by recurrent sonographic examinations that fail to reveal cause of lower abdominal pain or by unresolved ovarian cysts.

Further evaluation with MRI reveals additional findings like adhesions, endometriotic plaques or thickening of pelvic ligaments. I will review these additional features and list areas of interest in pelvis.

Conclusion

MRI evaluation in cases of suspected or known endometriosis reveals additional findings compared to sonography. Reviewing areas of interest helps in comprehensive diagnosis.

### P45 Pulmonary manifestations in advanced breast cancer

#### Shoubhi Bhatnagar (shoubhimd@gmail.com)

##### QEQM hospital, EKHUFT, London, UK

Learning objective

To review various forms of metastatic spread of breast cancer in lungs.

Content organisation

Breast cancer is one of the leading cause of mortality in the world. We often find advanced cases of breast malignancy with initial or subsequent presentation of lung metastases.

The spread in lungs varies from metastatic nodules to lymphangitic spread. Knowledge of the same is essential in identifying these changes.

I will review various forms of pulmonary manifestations in advanced breast cancer and discuss in brief about mode of spread.

Conclusion

Identifying various modes of pulmonary metastases in advanced breast cancer is key in correct diagnosis and alerts the radiologist about potential errors in subtle cases.

### P46 Computed tomography colonography in elderly patients, a safe and accurate colonic examination

#### N Raja, M Adeleye, D Kotecha, A Verma, B Billimoria

##### Kettering General Hospital NHSFT, Kettering, United Kingdom

###### **Correspondence:** N Raja (neelraja@doctors.org.uk)

Aim

CT colonograms can be performed for patients who fail colonoscopy for detection of colorectal cancer (CRC). In this study we explore the use of CTCs, in an elderly population who may not be suitable for further investigation/intervention.

Materials and Methods

We reviewed 1479 patients who had undergone a CTC between October 2015 and October 2018. We focused on patients aged ≥80 at the time of scanning. CTC reports were categorised into those with positive, indeterminate and no significant findings. All patients ≥80 years old were followed up (via their electronic records) to observe their outcomes.

Results

454 patients were aged ≥80 years old (30.7%) – mean & median age 84, range 80-97. 69 patients had positive colonic findings (15.2%). Of which, 31 had CRC reported, 22 had polyps reported and 16 had indeterminate findings. At follow up (range 8 – 44 months), none of the 385 patients, with nil significant colonic findings on CTC, have been diagnosed with CRC.

Conclusions

The yield of diagnosing colorectal cancer was 6.9% (31/454). CTC that reported negatively for colonic findings seems to protect patients for up to 44 months. The reports are generally accurate regarding significant colonic findings, especially when diagnosing CRC. This study confirms the safety and efficacy of CTC suggesting that it is an appropriate colonic investigation for elderly patients, first or second line.

### P47 Sclerotic bone lesions. Evaluation of response to therapy

#### S. De Luca, C. Carrera, C. Barale, P. Ramos, C. Benitez, F. Pomés, M. Saborido, E. Eyheremendy

##### Hospital Alemán. Buenos Aires. Argentina

###### **Correspondence:** S. De Luca (sdeluca@hospitalaleman.com)

Learning objectives

- Understand the concept of pseudoprogression of bone metastases.
- Understand the concept of osteoblastic reaction as criteria of response to treatment when there are other clinical and imaging findings that join the response to treatment.

Content organisation

In patients with malignancies that potentially metastasise to bone, the early diagnosis of bone metastasis is crucial to determine the prognosis and to define therapy.

The purpose of imaging is to identify early bone metastasis and to monitor response to therapy.

Tc 99m MDP based skeletal scintigraphy has been the standard method for the initial staging of the bone tumours. However it detects bone metastases at a relatively advanced stage of tumour infiltration, only after osteoblastic host reaction to tumour deposits has begun. More recently, F18 FDG-PET/CT, imaging which has various oncological applications, has been recommended as an important complementary tool in the detection of bone metastases and evaluation of therapy response.

Conclusions

A new sclerotic bone lesion in a patient treated for metastatic disease may reflect healing by sclerosis of previously hidden bone metastasis (pseudoprogression)

The correlation with the disease in other places, the humoral markers and studies as Tc 99m MDP based skeletal scintigraphy and F18 FDG-PET/CT can be useful in the distinction between these two possibilities.

### P48 Applications of Dual Energy Computed Tomography in Oncologic Imaging

#### Nils Grosse Hokamp, Simon Lennartz, David Maintz, Thorsten Persigehl

##### University of Cologne, Faculty of Medicine and University Hospital Cologne, Department of Diagnostic and Interventional Radiology, Cologne, Germany

###### **Correspondence:** Nils Grosse Hokamp (nils.grosse-hokamp@uk-koeln.de)

Learning Objectives

The aim of this educational exhibit is to review the basic principles of dual energy computed tomography (DECT) and the available technological approaches to DECT-imaging. Further, available reconstructions from DECT will be discussed with a particular focus towards their application in oncological imaging.

Content organisation

The fundamental concepts behind DECT imaging will be reviewed briefly. This information is prerequisite for the understanding of different technological approaches to DECT. This first introductory part will include several graphs and illustrations to neatly illustrate the findings. Available reconstruction will be discussed case based. Here, studies from our hospitals in- and outpatient practices will be used to neatly illustrate the potential of DECT imaging in these patients. As any new technology has its limitations or drawbacks, possible obstacles for technological and diagnostic workflow will be referred. Last, a brief review on the most recent studies will try to give an outlook on current trends in DECT-applications in oncologic patients.

Conclusions

DECT is a widespread available technology that allows for reconstruction of so-called spectral reconstructions in addition to conventional CT images. A knowledge of physics and technology-basics is important to understand the variety of available (spectral) reconstructions and to evaluate their possible applications in oncologic imaging.

### P49 Dual-Energy-CT to improve visualisation of hepatic metastasis: Proof-of-concept in a 3D-printed phantom and patient validation

#### Nils Grosse Hokamp, Stefan Haneder, Simon Lennartz, David Maintz, Thorsten Persigehl

##### University of Cologne, Faculty of Medicine and University Hospital Cologne, Department of Diagnostic and Interventional Radiology, Cologne, Germany

###### **Correspondence:** Nils Grosse Hokamp (nils.grosse-hokamp@uk-koeln.de)

Aim

There is a well-known boost of iodine associated-attenuation in low keV virtual monoenergetic images (VMI_low) which is frequently used to improve visualisation of lesions and structures that take up contrast media. This study aimed to evaluate this contrast vice versa: Does increased attenuation of the liver parenchyma allow for improved visualisation of little or none-enhancing lesions?

Methods

A 3D-printed phantom mimicking the shape of a human liver exhibiting a lesion in its center was designed and printed. Both, parenchyma- and lesion-mimic were filled with iodine-solutions of different concentrations exhibiting an attenuation of 80, 100, 120 HU for parenchyma- and 0, 40, 60 HU for lesion-mimics.

Further, a total of 75 patients with MRI or follow-up proven cysts and/or hepatic metastases was included. Imaging was performed on a spectral detector CT scanner (SDCT) and VMI of 40-120 keV as well as conventional images (CI) were reconstructed. Regions of interest were placed in lesion and parenchyma on CI and transferred to VMI. Signal- and contrast-to-noise ratio were calculated.

Results

In phantoms, Using 40keV images, mildly hypodense lesions in poorly attenuating liver parenchyma exhibited a similar CNR as compared to cysts in conventional images (5.8±0.9 vs 6.4±0.8; p≤0.05). The same tendency was observed in patients, again cysts in CI yielded similar values as metastases in VMI_low (4.4±1.2 and 3.9±1.8, respectively, p≤0.05).

Conclusion

The improved attenuation of the liver outweighs increasing in attenuation of the lesion itself. Hence, VMI_low from SDCT allow for an improved visualisation of hypodense focal liver lesions.

### P50 Iodine overlays from spectral detector computed tomography can improve assessment of peritoneal carcinomatosis

#### S Lennartz, D Zopfs, N Abdullayev, K Slebocki, M Le Blanc, C Wybranski, D Maintz, T Persigehl

##### University of Cologne, Faculty of Medicine and University Hospital Cologne, Department of Diagnostic and Interventional Radiology, Cologne, Germany

###### **Correspondence:** S Lennartz (Simon.Lennartz@uk-koeln.de)

Aim

Peritoneal carcinomatosis (PC) is prognostically relevant for oncologic patients. In computed tomography, it may be hard to differentiate from postoperative peritoneal changes, particularly in early stages. Our aim was to determine whether PC could be diagnosed more accurately when combining spectral detector CT (SDCT)-derived iodine overlays (IO) and conventional images (CI) compared to CI only.

Methods

60 oncologic patients, 30 with histopathologically proven PC and 30 with non-malignant peritoneal alterations confirmed by follow-up/PET-CT who received portal-venous phase abdominal SDCT were retrospectively identified. Two experienced and two less experienced radiologists evaluated presence of PC and rated conspicuity/diagnostic certainty for up to 5 lesions per patient using 5-point Likert scales. Patients were randomised and assessed in a session comprising solely CI and a second one which additionally included IO, between which a 6-week latency was interposed to minimise recognition bias.

Results

For less experienced reviewers, IO led to an increased sensitivity/specificity (CI: 0.78/0.83 vs. CI+IO:0.82/0.88) for presence of PC. Experienced radiologists showed a higher specificity when employing IO as well. However, this was associated with a lower sensitivity (Sensitivity/Specificity: CI: 0.92/0.80 vs. CI+IO:0.73/0.82). Pertaining to patients who had undergone abdominal surgery, the rise in specificity averaged over all readers was highest (CI:0.78 vs. CI+IO:0.91). While diagnostic certainty was comparable, ratings for lesion conspicuity were significantly higher for the combination of CI/IO (4(3-5)) compared to CI only (3(3-4);p<0.05).

Conclusion

IO can improve visual differentiation between benign and metastatic peritoneal lesions, particularly in patients who underwent abdominal surgery and for less experienced radiologists.

### P51 Differentiation of benign lung nodules and metastases: combination of texture analysis and iodine maps

#### S Lennartz^1^, A Mager^1^, N Große Hokamp^1^, S Schäfer^2^, D Maintz^1^, T Persigehl^1^

##### ^1^University of Cologne, Faculty of Medicine and University Hospital Cologne, Department of Diagnostic and Interventional Radiology, Cologne, Germany; ^2^Mint medical GmbH, Heidelberg, Germany

###### **Correspondence:** S Lennartz (Simon.Lennartz@uk-koeln.de)

Purpose

Differentiation between benign and metastatic lung nodules in oncologic patients is an important clinical issue. Dual-energy CT-derived iodine maps and texture analysis have been previously investigated to this regard. The purpose of this study was to analyze if the combination of these two techniques could be beneficial. Hence, we investigated the accuracy with which first order texture features derived from conventional images and iodine maps could differentiate benign from metastatic lung nodules when being applied to a machine learning classifier.

Methods and Materials

We retrospectively identified 184 oncologic patients who received spectral detector CT (SDCT) of the chest (IQon, Philips): 85 patients with 170 benign lung nodules confirmed by prior/follow-up CT (constant in size for ≥6 months) or histopathology and 99 patients with 425 lung metastases verified by histopathology, 18F-FDG-PET-CT or unequivocal change during treatment. All lesions were segmented semi-automatically and volumetric attenuation/iodine concentration as well as referring texture features (entropy, kurtosis, mean of the positive pixels, skewness, uniformity of the positive pixels) were acquired. Features were tested individually, and the most powerful features were transferred to a K-nearest neighbor classifier.

Results

K-nearest neighbor classification with leave one out cross-validation yielded a sensitivity/specificity/accuracy of 0.94/0.68/0.87 for conventional features and 0.95/0.65/0.87 for conventional features in combination with iodine entropy which was the most powerful iodine-derived feature.

Conclusion

Based on first-order texture features derived from conventional images, machine-learning facilitated accurate differentiation of benign lung nodules and pulmonary metastases. Iodine entropy only slightly improved sensitivity with comparable diagnostic accuracy.

### P52 Decision making and acceptability in use of Whole Body MRI in asymptomatic subjects: pilot study

#### D. Busacchio, K. Mazzocco, P. Pricolo, F. Zugni, P. Summers, M. Masiero, G. Pravettoni, G. Petralia

##### Applied Research Division for Cognitive and Psychological Science European Institute of Oncology Milan Italy, Department of Oncology and Hemato-Oncology University of Milan Milan Italy, Department of Radiology European Institute of Oncology Milan Italy, Department of Biomedical and Clinical Sciences University of Milan Italy, Post graduation School in Radiodiagnostics University of Milan Italy

###### **Correspondence:** D. Busacchio (derna.busacchio@ieo.it)

Aim

To investigate acceptability and decision making process of Whole Body Magnetic Resonance Imaging (WB-MRI) for cancer screening in asymptomatic subjects.

Materials and methods

Sixty-five asymptomatic subjects (mean age =51; F=64%) scheduled for a WB-MRI, filled psychological measures to assess WB-MRI acceptability. Perceived usefulness and discomfort before and after examination were scored by subjects using a ordinal scale to one to five. A decision-tree methodology was used to identify factors affecting the decision to under go a WB-MRI, that were grouped into five categories: certainty of diagnosis, psychological wellbeing, safety of procedure, validity of the test, cost. A cluster was performed for the study subject.

Results

Subjects reported high levels of WB-MRI perceived usefulness with an increase after the examination (respectively, M=3.94; M=3.48; p<.01). Reported discomfort was associated mainly with feelings of confinement and duration of the examination. A significant difference was found between the expectation of the discomfort imagined before WB-MRI and the actually experienced discomfort (respectively, M=3.71 and M=3.06; p<0.05). The cluster analysis identified five groups of subjects. Certainty of diagnosis was the most important factor in all clusters. In only two clusters psychological well-being was a driver for WB-MRI. Safety of the procedure was crucial in only other two clusters. The test validity was central only in one cluster.

Conclusions

WB-MRI was perceived as useful because it was considered able to provide certainty of diagnosis. The discomfort expectated before the examination, related to the feeling of confinement and duration, should be addressed with better comunication.

### P53 Radiologic imaging characteristics and PET CT in determining the pathologic grade of hepatocellular carcinoma

#### Dr. Yashasvini K, Dr. Shivakumar Swamy S, Dr. Sudhakar S, Dr. Indiresh Desai, Dr. Mahesh A, Dr. Avinash Kesari, Dr. Nikita Jain, Dr. Kumar Kallur, Dr. Ajai Kumar

##### Health Care Global Enterprises, Bangalore, Karnataka, India

###### **Correspondence:** Shivakumar Swamy S (yashukswamy@gmail.com)

Aim

Our study aimed at evaluating the various imaging appearance of HCC on PETCT and sensitivity in predicting the histopathologic grade of HCC accurately.

Materials and methods

62 histologically proven cases of HCC underwent retrospective analysis. The mean tumour volume, multicentricity, enhancement pattern, ancillary findings including vascular thrombosis and distant metastases were evaluated along with SUV max of the hepatic lesions.

Results

Out of 30 -well-differentiated, 15 -moderately differentiated and 17 - poorly differentiated cases of hepatocellular carcinoma, multicentricity and ill-defined lesions were in 82% poorly differentiated carcinomas and 40% of moderately differentiated carcinomas. 58% of poorly differentiated carcinomas and 20% of moderately differentiated carcinomas had atypical enhancement patterns. 60% of well differentiated carcinomas were unicentric and well defined with classical enhancement in 70% of cases. The range of SUVmax was from 7-25 in poorly differentiated tumours, 5 -12 in moderately differentiated carcinomas and 3-11 in well differentiated tumours. Vascular thrombosis, necrosis, distant skeletal and pulmonary metastases was a predominant feature in poorly differentiated carcinomas.

Conclusion

In conclusion ill-defined multicentric lesions with vascular thrombosis, distant metastases, atypical enhancement and higher SUVmax are usually poorly differentiated hepatocellular carcinomas on histopathology. PETCT is a sensitive modality to predict tumour grade and distant metastases.

### P54 Intra-individual variability of iodine quantification of the intravascular and renal blood pool enabled by spectral detector CT

#### S Lennartz, N Abdullayev, D Zopfs, V Neuhaus, D Maintz, T Persigehl, S Haneder, N Große Hokamp

##### University of Cologne, Faculty of Medicine and University Hospital Cologne, Department of Diagnostic and Interventional Radiology, Cologne, Germany

###### **Correspondence:** S Lennartz (Simon.Lennartz@uk-koeln.de)

Aim

Iodine maps provided by dual-energy CT have been frequently investigated for various oncologic imaging applications. Yet, data on reproducibility of iodine measurements in vivo which is paramount for clinical application in oncologic follow-up, are sparse. The aim was to examine the intra-individual variability of iodine quantification in patients who received multiple abdominal spectral detector CT (SDCT) exams.

Materials and Methods

79 patients with 2 (n=53) or 3 (n=26) clinical, biphasic (arterial/venous) abdominal SDCT scans were retrospectively included. Quantitative values for attenuation [HU] and iodine concentration [mg/ml] were measured by placing two regions of interest in the aorta, inferior caval vein and renal cortices, respectively. Modified variation coefficients (MVC) were calculated to investigate intra-individual consistency of iodine and HU measurements.

Results

Consistency of attenuation and iodine concentration was significantly lower in arterial phase than in venous phase images (p ≤ 0.05). Regarding arterial phase attenuation, median MVC was -1.8 (-20.5-21.3) % within the aorta and -6.5 (-44.0 – 48.7) % within the renal cortex while in the portal venous phase it was 0.62 (-11.1-11.7) % and -1.6 (-16.2-10.6) %, respectively. With regards to iodine quantification, arterial phase MVC was -2.5 (-22.9-28.4) % within the aorta and -5.8 (-55.9 – 29.6) % within the renal cortex. Corresponding portal venous phase MVCs were -0.7 (-17.9-16.9) % and -2.6 (-17.6-12.5) %.

Conclusion

Iodine quantification of the intravascular and renal blood pool shows highest intra-individual consistency in venous-phase images (overall MVC: ±15 %) whereas arterial phase measurements are subject to greater variability; this should be regarded when applying this technique for oncologic follow-up.

### O1 Pretreatment intravoxel incoherent motion diffusion weighted imaging predicts treatment outcome in nasopharyngeal carcinoma

#### Qamar S^1^, King AD^1^, Ai QY^1^, Poon DMC^2^, Wang YX^1^, Chen W^1^

##### ^1^Department of Imaging and Interventional Radiology, The Chinese University of Hong Kong, Prince of Wales Hospital, Shatin, Hong Kong S.A.R., China; ^2^Department of Clinical Oncology, State Key Laboratory of Translational Oncology, The Chinese University of Hong Kong, Prince of Wales Hospital, Shatin, Hong Kong S.A.R., China

###### **Correspondence:** King AD (king2015@cuhk.edu.hk)

Aim:

To identify pretreatment intravoxel incoherent motion diffusion weighted imaging (IVIM-DWI) parameters for the prediction of outcome in nasopharyngeal carcinoma (NPC).

Materials and Methods:

Pretreatment IVIM-DWI was performed on 102 NPC patients (mean age± standard deviation, 49.4±10.7 years). The mean values of the pure diffusion coefficient (D), pseudo-diffusion coefficient (D*) and perfusion fraction (f) of the primary tumour were calculated by a biexponential fit. The associations of age, sex, treatment, stage and IVIM-DWI functional parameters with locoregional failure-free survival (LRFS), distant metastasis failure-free survival (DMFS), and progression-free survival (PFS) were assessed by Cox proportional hazards analysis.

Results:

Failure occurred at the locoregional and distant sites in 22% and 16%, and disease progression in 31% of cases (median follow-up time of 54 months). At univariate analysis, the mean D was associated with LRFS (hazard ratio [HR], 1.044; 95% confidence interval [CI]: 1.004- 1.087; P value= 0.032) but D* and f were not associated with survival endpoints (p >0.05). D remained an independent predictor of LRFS (HR, 1.055; 95% CI: 1.007-1.105; P=0.025 after adjustment for treatment as a confounding factor.

Conclusion:

Pretreatment IVIM-DWI at initial staging has the potential to be a predictor of outcome in patients with NPC.

### O2 Pretreatment ADC differences among human papillomavirus positive and negative oropharyngeal squamous cell carcinoma

#### Deschuymer S, De Keyzer F, Nuyts S, Vandecaveye V

##### KU Leuven – University of Leuven; University Hospitals Leuven; Leuven; Belgium

###### **Correspondence:** Deschuymer S (sarah.deschuymer@uzleuven.be)

Aim:

Human papillomavirus (HPV) positive and negative oropharyngeal squamous cell carcinoma (OPC) are two distinct disease entities with different biology, molecular profile, treatment response and outcome. This study aimed to identify their radiological differences based on quantitative diffusion-weighted (DW) MRI.

Methods:

One hundred sixty-three patients with histologically proven OPC were prospectively analysed. OPC were considered HPV positive if more than 70% diffuse nuclear and cytoplasmic p16 immunohistochemistry staining was present. 1.5 T or 3 T MRI with echo-planar DW sequences at 6 b-values (0, 50, 100, 500, 750 and 1000 s/mm²) were acquired before chemoradiotherapy treatment.

The region of interest (ROI) encompassing the entire primary tumour volume, was manually drawn on the apparent diffusion coefficient (ADC) map by an experienced head and neck radiologist.

Various first-order parameters, ADC mean, ADC min, ADC max, were extracted from the ROI and were compared between HPV positive and HPV negative OPC with the Mann-Whitney-U test. The significance threshold was set at a p-value of <0.05.

Results:

Fifty-seven (35%) tumours were HPV positive. Between HPV negative and HPV positive OPC, there was a significant difference in ROI-based ADC mean (median 1.05x10-3mm²/s vs. 0.92 x10-3mm²/s, p<0.0001) and ADC min (median 0.45x10-3mm²/s vs. 0.38 x10-3mm²/s, p=0.02). ADC max only showed a trend towards lower values for HPV positive OPC (p=0.07).

Conclusion:

Primary tumour ADC mean and ADC min values were significantly lower in HPV positive OPC. To our knowledge, this is the largest cohort comparing quantitative DW-MRI parameters with HPV status. NCT01829646

### O3 Deep-learning automatic delineation of primary tumour volume in nasopharyngeal carcinoma on T2W fat-suppressed MR images

#### Lun M. Wong, Qiyong Ai, Lin Shi, Ann D. King

##### The Chinese University of Hong Kong, Prince of Wales Hospital, Hong Kong SAR, PRC

###### **Correspondence:** Lin Shi (fromosia@link.cuhk.edu.hk)

Aim

Accurate determination of primary tumour volume (PTV) in nasopharyngeal carcinoma (NPC) on magnetic resonance (MR) images is important for research, staging and clinical management. However, manual delineation is laborious with high inter-observer variability. The aim of this study is to introduce artificial-intelligent (AI) to delineate the primary tumour on contrast-free images.

Materials and methods

We have designed a convolutional-neural-network architecture, modified based on Attention U-Net, to compute pixel-wise probability map of tumour presence from MR input images. We incorporated textural information, computed from local binary pattern and local neighborhood difference pattern, with the attention layers to weight features extracted by the network.

The study was performed in 404 patients with NPC who had undergone MR imaging with a standard protocol which included T1W, T2W- fat-suppressed (FS), contrast-enhanced-T1W (CE-T1W) and CE-T1W-FS sequences.

The network was trained with contours delineated manually on the T2W-FS images by an expert, referencing all MR sequences for local tumour extent. The trained network received T2W-FS images only during validation. Four-fold cross-validation was performed to evaluate the network performance using Dice similarity coefficient (DSC). Intra-class correlation coefficient (ICC) between the PTV of AI-generated and manually drawn contours was also evaluated.

Results

The DSC medians of each fold were 0.807, 0.792, 0.811 and 0.794. Overall DSC median is 0.800 with an inter-quartile-range of 0.088. Strong and significant correlation between AI and manually calculated PTV was observed (ICC=0.952, p < 0.001).

Conclusions

We have proposed a robust, automatic, deep-learning-based delineation method on contrast-free MR sequence (T2W-FS) for NPC.

### O4 Differentiation of solid hepatic lesions with intravoxel incoherent motion diffusion-weighted imaging using a volumetric approach

#### M Puglia^1^, MA Bali^2^, S Picchia^3^, M Orton^4^, S Doran^4^, T Feiweier^5^, DM Koh^6^, G Morana^7^

##### ^1^Department of Radiology, Santa Maria delle Grazie Hospital, Pozzuoli (Naples Italy); ^2^Department of Radiology, Erasme Hospital, Université Libre de Bruxelles (Belgium); ^3^Department of Radiology, University “La Sapienza” I.C.O.T Hospital, (Latina Italy); ^4^CRUK Cancer Imaging Centre, Institute of Cancer Research (Sutton UK); ^5^Siemens AG, (Erlangen Germany); ^6^The Royal Marsden Hospital Department of Radiology (Sutton UK); ^7^Department of Radiology Ca’Foncello Regional Hospital (Treviso, Italy)

###### **Correspondence:** M Puglia (martapuglia@alice.it)

Aim:

To evaluate the role of IVIM model and ADC value in the characterisation of solid focal liver lesions (FLLs) using a volumetric approach among four groups: Benign VS Malignant, FNH VS Adenoma, Hypervascular Benign VS Hypervascular Malignant, Hypervascular Malignant VS Hypovascualr Malignant.

Methods:

100 patients with 104 FLLs (77 malignant-27 benign) underwent liver 1.5-T MRI for routine examination sequences using IVIM diffusion-weighted imaging with 11 b values (0-800 s/mm2). Apparent diffusion coefficient (ADC) and IVIM-derived parameters, such as pure diffusion coefficient (D), pseudodiffusion coefficient (Ds), perfusionfraction (f) and their product (fDs) were calculated using a volumetric approach and compared among the different groups. A receiver operating characteristic curve analysis was performed to assess their diagnostic value.

Results:

ADC and D were significantly lower in the malignant group than in the benign group (ADC mean: 1.38± 0.36 VS 1.60 ± 0.58; D mean 0.88 ± 0.29 VS 1.05 ± 0.30) × 10−3 mm2/s, and in the hypervascular malignant than hypervascular benign (ADC mean 1.38± 0.33 VS 1.60 ± 0.72; D mean: 0.90 ± 0.21 VS:1.05 ± 0.29) × 10−3 mm2/s. Ds did not show statistical differences. FDs was significantly lower in the hypovascular malignant than in the hypervascular malignant group (mean 9.96 ± 1.03 VS 12.7 ± 8.5)× 10−3 mm2/s. F(skweness and kurtosis) was also lower in the hypovascular malignant than in the hypervascular malignant group

Conclusion:

Compared with ADC, the IVIM model values using a volumetric approach improves the characterisation of FLLs.

This abstract has been previously published.

### O5 Pancreatic screening in high risk patients: is fast non-contrast MR protocol feasible? A proposal

#### F. Maio^1^, V. Pasqualino^2^, L. Bertana^3^, S. Venturini^3^, V. Cantoni^1^, M. Fusaro^3^, G. Morana^3^

##### ^1^Federico II University Naples, Italy; ^2^Padova University Padova, Italy; ^3^Ca’ Foncello Hospital Treviso, Italy

###### **Correspondence:** F. Maio (francescamaio9@gmail.com)

Aim:

To validate a non-contrast fast MRI protocol for high risk patients as a screening tool to detect pancreatic cancer (PC) in its earliest phase, compatible with an R0 resection.

Materials and Methods:

200 patients (>40yo) were selected from our radiological database. 100 were negative for pancreatic lesions, 50 were positive for cystic lesions and 50 were positive for solid lesions; all lesions were smaller than 28mm. Three readers with a high, medium and low experience analysed selected MRI sequences (single-shot T2w breath-hold on axial and coronal plans, GE T1w FS on axial plan, DWI and 2D/3D MRCP) independently, randomly and anonymously. Readers identified or excluded the presence of pancreatic lesion. Results of reading session were compared with the final diagnosis and divided into five different classes of lesion: cystic, solid (all), adenocarcinoma, PNET and solid excluding PNET; Mcnemar’s test was used to compare. Inter-observer agreement was determined according to the kappa statistic.

Results:

All readers showed high sensitivity and NPV in the identification of ADK (R1 100%-100%, R2 89%-98% and R3 83%-97%), with a good agreement to detect pancreatic lesions (k=0.52), especially ADK (k=0.82), PNET (>10mm) (k=0.70) and cystic lesions(k=0.87).

Conclusion:

A non-contrast fast MRI protocol can be proposed as a screening tool in high risk patients for PC, reducing the time lapse between the controls, giving more chances for an early diagnosis with a better outcome.

This abstract has been previously published.

### O6 Sclerosing Angiomatoid Nodular Transformation of the Spleen – Radiological/Pathological correlation and clinical features

#### Murphy G^1^, Miller B^1^, Paneesha S^2^, Kishore B^2^, Rudzi Z^3^

##### ^1^Departments of Radiology; ^2^Haematology; ^3^Pathology, University Hospitals Birmingham (Heartlands Hospital), Birmingham, UK

###### **Correspondence:** Murphy G (grainne.murphy@heartofengland.nhs.uk)

Aim:

The presentation aims to provide an overview of the imaging features of sclerosing angiomatoid nodular transformation of the spleen (SANT), with reference to clinical features and pathological correlation

Materials and Methods:

A series of 3 patients investigated at our institution for splenic lesions who were pathologically proven to have SANT will be presented and the imaging features discussed.

SANT is an uncommon benign splenic vascular lesion comprising angiomatoid nodules surrounded by dense fibrous tissue. It can provide a diagnostic dilemma when discovered on imaging as it can be mistaken for lymphoproliferative or metastatic disease. While most cases are discovered incidentally, up to 20% of patients are reported to have synchronous or metachronous malignancies and it may sometimes be associated with abdominal pain, anaemia or an elevated erythrocyte sedimentation rate.

Results:

Our presentation will demonstrate the imaging findings of SANT, with pathological correlation.

Teaching points:

SANT is an uncommon benign vascular splenic lesion which may be mistaken for primary or seondary malignancy

The usual imaging finding is a rounded splenic mass, which may be lobulated

Pathological findings are pathognomonic.

### O7 Accuracy of WB-DWI/MRI in diagnosis, staging and follow-up of gastric cancer

#### S. De Vuysere^1,2^, V. Vandecaveye^2^, R. Dresen^2^

##### ^1^Imelda Hospital Bonheiden Belgium; ^2^University Hospitals Leuven Belgium

###### **Correspondence:** S. De Vuysere (sofie.devuysere@gmail.com)

Aim:

Gastric cancer staging often includes both CT of abdomen and thorax and laparoscopy. However, a one-stop noninvasive technique might be a more valuable option. Therefore, we aimed to evaluate the accuracy of whole-body diffusion-weighted magnetic resonance imaging (WB-DWI/MRI) for diagnosis, staging and follow-up of patients with a suspicion of gastric cancer (recurrence).

Methods:

From November 2015 until April 2019, 33 patients with a suspicion of gastric cancer (recurrence) who underwent WB-DWI/MRI at 1.5T were retrospectively analyzed. Examinations consisted of axial DWI (b=50, 1000), coronal T2 images and axial contrast-enhanced T1 images and were evaluated by two experienced abdominal radiologists in consensus. Reference standard was histopathology, laparoscopy or a follow-up of >1 year.

Results:

Of the 33 patients, 2 patients had histopathologically confirmed gastritis. MRI was correct in 1 patient and wrongly diagnosed 1 patient with operable gastric cancer (accuracy: 32/33: 96.7%).

Nineteen patients were diagnosed with gastric cancer (16 primary cancers and 3 recurrences). All recurrences turned out to be inoperable. Of the 16 primary cancers, 8 were inoperable (2 confirmed by laparoscopy and 6 patients showed progressive disease during follow-up). The other 8 patients were able to undergo a curative surgery. WB-DWI/MRI was correct in all these patients.

The last 12 patients had a history of gastrectomy. WB-DWI/MRI showed no disease recurrence. This was confirmed by a negative follow-up for at least 1 year.

Conclusion:

WB-DWI/MRI is highly accurate for diagnosis, staging and follow-up of patients with suspected gastric cancer and might replace laparoscopy in many cases.

### O8 T2w, ADC and PET histogram analysis of rectal cancer after pCRT: correlation with TRG

#### F Crimì^1^, V Aldegheri^1^, G Spolverato^1^, C Lacognata^2^, P Zucchetta^2^, A Barison^1^, L Albertoni^2^, C Campi^1^, R Stramare^1^, E Quaia^1^

##### ^1^University of Padova, Padova, Italy; ^2^Azienda Ospedaliera di Padova, Padova, Italy

###### **Correspondence:** F Crimì (filippo.crimi@uniroma1.it)

Purpose:

To investigate the correlation among T2w images, ADC maps, 18 F-FDG PET images histograms in the volume of interest (VOI) of the primary lesion and the TRG in patients affected by LARC after pCRT.

Materials and Methods:

22 patients (17M) affected by LARC were prospectively enrolled and underwent 18 F-FDG PET/MRI for restaging after pCRT. The MRI protocol included an oblique-axial T2w-sequence and an axial DWI-sequence. ADC maps and PET images of the pelvis were re-sliced and re-oriented with a specific software (PMOD) in order to perfectly match to the T2w images. A region of interest (ROI) was manually drawn along the boundaries of each slice on the T2w images including the rectal cancer, obtaining a VOI; each ROI was then copied on the corresponding PET and ADC data-sets. Voxel-based SUVs, ADC values and T2w-intensity values were collected from the entire VOI and mean, skewness and kurtosis were calculated. Spearman’s correlation coefficient was applied to evaluate the correlation among the variables and TRG (according to Mandard).

Results:

Seven patients showed a complete regression (TRG1). A significant positive correlation was found between SUVs mean values (ρ=0.480; p=0.037) and TRG. No significant correlation was detected between other SUV, T2w and ADC parameters and TRG.

Conclusion:

The preliminary results of our study showed that post-pCRT histogram analysis of SUV values are predictor of TRG in LARC. Further studies on a larger sample are necessary to assess the role of ADC and T2w intensity signal histogram analysis parameters.

This abstract has been previously published.

### O9 The added value of delayed contrast-enhanced MRI in restaging of rectal cancer after neoadjuvant radiochemotherapy

#### R. C. Dresen, G. Servaes, F. De Keyzer, V. Vandecaveye

##### University Hospitals Leuven, Leuven, Belgium

###### **Correspondence:** R. C. Dresen (elleke.dresen@uzleuven.be)

Aim:

Standard rectal MRI protocol consists of multiplanar T2-weighted images and diffusion-weighted images and plays a major role in local staging of rectal cancer. Despite this advanced technique, adequate staging can still be challenging, mainly due to inherent limitations of the aforementioned sequences. This study evaluated the value of adding a delayed-enhancement T1-sequence (DE-MRI) to the standard protocol for accuracy of local staging as well as the prediction of clinical complete response after neoadjuvant radiochemotherapy (RCT).

Methods:

Between February 2016 and September 2017, 41 consecutive patients underwent delayed Gadovist-enhanced rectal MRI after RCT. All standard MRIs were retrospectively reviewed by a radiologist with 2 years’ experience. After 3 weeks, they were reviewed by the same radiologist in a random order with the added DE-MRI. Both staging methods were compared with each other and the reference standard (histopathology or follow-up) using McNemar’s test. Survival differences were calculated using log-rank testing.

Results:

Twenty-eight patients underwent total mesorectal excision, 13 patients were enrolled in a “watch-and-wait”-based follow-up. Significantly better results were found for T-staging with the DE-MRI (P=0.0016) and for prediction of clinical complete response (P=0.0047). Evaluation of nodal stage showed no significant improvement (P=0.1573).

Patients without lymph node metastases had a significantly better disease free survival (P=0.0416). Distant metastases free survival was significantly better in patients with ypT0-2 (versus ypT3-4, P=0.0028) and ypN0 (versus ypN1-2, P=0.0040).

Conclusions:

Adding a delayed-enhancement T1-weighted sequence provided a more accurate T-staging and a better prediction of clinical complete response compared to the standard MRI protocol.

### O10 Determinants of ADC in the bone marrow of healthy individuals: effects of sex and age

#### A. Colombo, L. Bombelli, P. Summers, M. Bellomi, G. Petralia

##### Istituto Europeo di Oncologia, Milano, Italia

###### **Correspondence:** A. Colombo (alberto.colombo@ieo.it)

Aim:

Of increasing interest is the possibility of obtaining quantitative information from WB-MRI images, including the Apparent Diffusion Coefficient (ADC) derived from the diffusion weighted images. It is known that the ADC measured in the bone metastases may differ from that of normal bone marrow and it changes following therapy, but there is little evidence of the actual value of normal bone marrow as well as of the effects of physiological factors. The aim of this study is to evaluate how sex and age affect the ADC values measured in the bone marrow of healthy individuals.

Materials and Methods:

We have processed the diffusion weighted images of WB-MRI examinations in 75 healthy individuals, 39 men and 36 women aged 30 to 78 years. The mean value of ADC in bone marrow (ADCbm) was estimated by fitting the histogram extracted from the images with a semi-automatic segmentation technique. Gender differences were assessed with the Mann-Whitney U test, while age correlation was assessed with the Spearman correlation coefficient (ρ).

Results:

Overall ADCbm was 450.8±12.4 μm/s (95% IC) and no significant correlation with age was observed (ρ=-0.2, p=0.09). The values of ADCbm were significantly higher (p<0.01) in women, 477.3±17.7 μm/s (95% IC), than in men, 426.3±14.0 μm/s (95% IC). Moreover, in women there was a significant negative correlation between ADCbm and age (ρ=-0.42, p<0.05), which was missing in men (ρ=0.02, p=0.92).

Conclusions:

There was a significant difference in the ADCbm values between men and women. In addition, we observed a negative correlation between ADCbm and age in women.

### O11 Quantitative FDG PET/CT parameters in non-small cell lung cancer patients: pathology characteristics and prognosis

#### Mehrdad Bakhshayesh-karam^1^, Abtin Doroudinia^2^, Seyedeh Marzieh Mohaghegh^3^, Mohammad behgam shadmehr^4^, Adnan khosravi^2^, Azizollah abbasi dezfouli^4^; Abbas Yousefikoma^3^; Payam Mehrian^5^; Habib Emami^6^

##### ^1^Pediatric Respiratory Diseases Research Center, National Research Institute of Tuberculosis and Lung Diseases (NRITLD), Shahid Beheshti University of Medical Sciences, Tehran, Iran; ^2^Chronic Respiratory Diseases Research Center, National Research Institute of Tuberculosis and Lung Diseases (NRITLD), Shahid Beheshti University of Medical Sciences, Tehran-Iran; ^3^Lung Transplantation Research Center, National Research Institute of Tuberculosis and Lung Diseases (NRITLD), Shahid Beheshti University of Medical Sciences, Tehran, Iran; ^4^Tracheal Diseases Research Center, National Research Institute of Tuberculosis and Lung Diseases (NRITLD), Shahid Beheshti University of Medical Sciences, Tehran, Iran; ^5^Telemedicine Research Center, National Research Institute of Tuberculosis and Lung Diseases (NRITLD), Shahid Beheshti University of Medical Sciences, Tehran, Iran; ^6^Tobacco prevention and control Research Center, National Research Institute of Tuberculosis and Lung Diseases (NRITLD), Shahid Beheshti University of Medical Sciences, Tehran, Iran

###### **Correspondence:** Abtin Doroudinia (abtin1354@gmail.com)

Aims:

The aim of this study was to investigate correlation of 18F- fluorodeoxyglucose (FDG) Positron Emission Tomography/Computed Tomography (PET/CT) findings in non-small cell lung cancer (NSCLC) patients with primary tumour pathology characteristics and patient’s prognosis.

Methods:

We included 125 NSCLC patients referred for initial staging FDG PET/CT scan. The primary tumour (T), regional lymph node metastases (N) and distant metastases (M) were evaluated on FDG PET/CT images. Standard Uptake Value (SUV) max, SUV mean, Metabolic Tumour Volume (MTV) and Total Lesion Glycolysis (TLG) parameters were calculated separately for each T, N, M lesion and also for whole body. Statistical analysis including Student t-test, Kruskal-Wallis test, Mann-Withny test and Kaplan–Meier curves used to evaluate correlation of PET/CT quantitative parameters with tumour pathology and prognosis.

Results:

The patients followed for 19.28(±11.42) months. There was significant correlation between quantitative FDG PET/CT parameters of primary lung cancer (p=0.00), metastases (p=0.014), whole body MTV (p=0.045) and whole body TLG (p=0.002) with tumour pathology. There was also significant correlation between quantitative parameters of primary tumour (p=0.00) and regional lymph node metastases (p=0.048) with tumour initial stage. There was significant prognostic value for whole body TLG (p=0.01) and the cut point of 568 was reached to differentiate better survival outcome for patients with lower whole body TLG values.

Conclusion:

We demonstrated statistically significant correlation between FDG PET/CT quantitative parameters and primary NSCLC pathology and initial stage characteristics, as well as patient’s prognosis. We recommend incorporating PET/CT quantitative parameters, more specifically TLG values into clinical PET/CT reports.

Financial disclosure

Authors of this manuscript acknowledge that they have all contributed to this work significantly.

### O12 Imaging features of fumarate-hydratase deficient renal cancers: case series and review of literature

#### Nikolovski I, Vargas HA

##### Memorial Sloan Kettering Cancer Center, New York, USA

###### **Correspondence:** Nikolovski I (ines.nikolovski@gmail.com)

Aim:

To evaluate the imaging features of Fumarate-Hydratase (FH) Deficient Renal Cancers on computed tomography (CT), magnetic resonance imaging (MRI) and positron emission tomography (PET).

Methods:

Single-site retrospective analysis of histologically confirmed FH-deficient renal cancer, or patients with renal cancer and Germline FH mutation. Sixteen adults (mean age 45 years, range 20-73 years) with CT (n=13), MRI (n=12) and PET (n=3) performed before histologic evaluation and genetic testing were included. Additionally, a systematic literature review of FH-deficient renal cancer and reported imaging findings was conducted (search criteria “fumarate hydratase, renal cancer, CT, PET, MRI” date range: 01/01/2000-05/01/2019) and summarised.

Results:

Renal tumours were unifocal in 88% of cases, multifocal 12%, infiltrative 63% and circumscribed 37%. Predominantly cystic masses were seen in 44%. Tumours invaded the renal sinus in 81%, and renal vein tumour thrombus was seen in 38%. All tumours were heterogenously enhancing, heterogenous in T2 signal, and had diffusion restriction. Of the 3 patients imaged with PET, all were hypermetabolic (mean SUVmax 16.4, range 9.6-21.9). Retroperitoneal nodal metastases were present in 69% and distant metastases in 75%.

Conclusion:

FH-deficient renal cancers usually present at an advanced stage and in a younger population than non FH-deficient renal cancers. In our case series most tumours also invaded the renal sinus, were heterogenous in T2 signal and had diffusion restriction. The small number of cases that had PET imaging showed high metabolic activity, which may differentiate them from some non-FH deficient renal cancers, though larger numbers are needed to explore this further.

